# A spatially-dependent fragmentation process

**DOI:** 10.1007/s00440-024-01325-w

**Published:** 2024-10-18

**Authors:** Alice Callegaro, Matthew I. Roberts

**Affiliations:** 1 Department of Mathematics, Technical University of Munich, Boltzmannstr. 3, 85748 Garching, Germany; 2 Department of Mathematical Sciences, University of Bath, Bath, BA2 7AY UK

**Keywords:** Fragmentation, Branching random walk, Multitype, Growth rate, Primary 60J80, Secondary 60J25

## Abstract

We define a fragmentation process which involves rectangles breaking up into progressively smaller pieces at rates that depend on their shape. Long, thin rectangles are more likely to break quickly, whereas squares break more slowly. Each rectangle is also more likely to split along its longest side. We are interested in how the system evolves over time: how many fragments are there of different shapes and sizes, and how did they reach that state? Using a standard transformation this fragmentation process with shape-dependent rates is equivalent to a two-dimensional branching random walk in continuous time in which the branching rate and the direction of each jump depend on the particles’ position. Our main theorem gives an almost sure growth rate along paths for the number of particles in the branching random walk, which in turn gives the number of fragments with a fixed shape as the solution to an optimisation problem. This is a result of interest in the context of spatial branching systems and provides an example of a multitype branching process with a continuum of types.

## Introduction

### Fragmenting rectangles

A fragmentation process describes the breaking up of a structure into pieces, and occurs naturally in many situations. Mathematically, fragmentation processes have been a subject of active research in probability for at least 20 years, incorporating several varieties, including homogeneous fragmentations [9], self-similar fragmentations [10], and growth fragmentations [13]. The textbook of Bertoin [11] gives an excellent introduction to this rich mathematical theory. It begins by listing some real-world examples of phenomena that might be considered fragmentation processes, including “stellar fragments in astrophysics, fractures and earthquakes in geophysics, breaking of crystals in crystallography, degradation of large polymer chains in chemistry, DNA fragmentation in biology, fission of atoms in nuclear physics, fragmentation of a hard drive in computer science,” and particularly valid from a mathematical point of view, “evolution of blocks of mineral in a crusher.”

However, the classical mathematical definition of a fragmentation process insists that (again quoting Bertoin [11]) “each fragment can be characterized by a real number that should be thought of as its size. This stops us from considering the spatial position of a fragment or further geometrical properties like its shape; physicists call such models mean field.” In [12], Bertoin does analyse a multitype model where fragments can take finitely many types, but in applications there is often a continuum of possible shapes, e.g. [5, 19, 21]. More details on the related literature are postponed to Sect. 1.9.

**Fig. 1:**
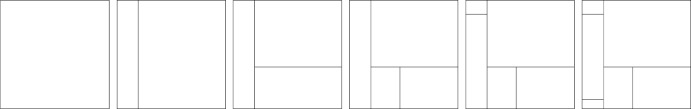
We begin with a square, which splits vertically into two rectangles. One of these then splits horizontally, and the process continues. Thinner rectangles are more likely to split first

**Fig. 2:**
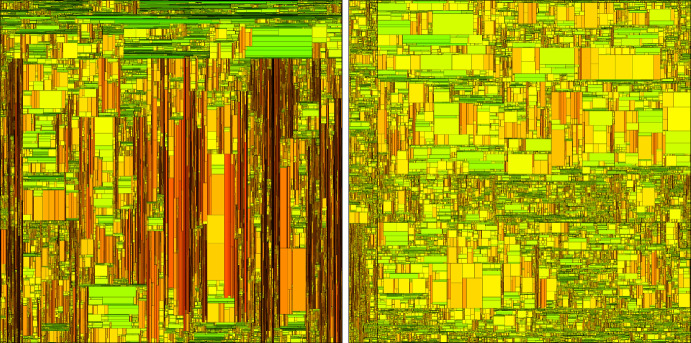
On the left: a homogeneous model, where every rectangle splits at rate 1 and splits horizontally or vertically with probability 1/2 each. Some sketch proofs for this model, which can be decomposed into two independent copies of a one-dimensional branching random walk, were provided in [20]. On the right: our model where long, thin rectangles split faster, and are more likely to split along their longest side. Tall rectangles are coloured red, fat rectangles are coloured green, and squares are coloured yellow

We consider a shape-dependent fragmentation process as follows. Begin with a square of side length 1. After a random time, the square breaks into two rectangular pieces, uniformly at random. Each of these pieces then repeats this behaviour independently, except that long, thin rectangles break more quickly, and are more likely to break along their longest side. This model is designed to mimic a physical crushing process, where long, thin pieces of rock are likely to break more easily than more evenly-proportioned pieces. See Figs. 1 and 2.

We work in two dimensions to keep notation manageable, but our proofs should in principle work in three or more dimensions. We also make a particular choice for the splitting rule—that is, the functions that decide how a fragment’s shape affects its splitting rate and the direction in which it breaks—but our methods should be adaptable to a variety of shape-dependent fragmentation models. We give some guidance in Sect. 1.8 as to what to expect for other splitting rules, both in terms of results and the difficulties arising in the proofs.

A key observation in the study of (mathematical) fragmentation processes is that they satisfy the branching property, in that the future evolution of one fragment, given its current state, does not depend on the other fragments. This enables us to use branching tools in the analysis of fragmentation processes. For example, if we consider the negative logarithm of the sizes of the fragments of a homogeneous fragmentation, then we obtain a continuous-time branching random walk. Bertoin’s multitype fragmentation, under the same transformation, becomes a multitype branching random walk. In the same way, our system of fragmenting rectangles can also be thought of as a multitype branching random walk, but one with uncountably many types.

Understanding spatially-dependent branching systems is an important problem in its own right, since almost any real-world application of branching tools—from nuclear reactors [28, 30] to the spread of disease [22, 26]—involves spatial inhomogeneity. One purpose of this paper is to contribute new techniques to the rigorous mathematical investigation of spatially-dependent branching structures more generally. In particular, we introduce novel methods for controlling the growth of the population at small times, and a delicate coupling to bound the spatially-dependent branching rate from above and below while keeping errors small.

Analysing branching systems with uncountably many types is notoriously difficult. Even multitype Galton–Watson processes with countably many types are beyond the scope of standard tools [29], hence the restriction to finitely many types in most papers on multitype branching systems, including [12]. Our model includes not just a continuum of types, but a two-dimensional set of possibilities. To add to the difficulty, since we split our rectangles using uniform random variables and then take negative logarithms, and $-\log U$ is exponentially distributed, the jump distribution of our multitype branching random walk does not satisfy the strong Cramér condition, and even when rescaled the sample paths include macroscopic jumps. Our analysis is therefore highly technically challenging.

### The model

As mentioned above, we work in continuous time, and begin with a square of side-length 1. At any time, each rectangle of base *b* and height *h* independently splits at rate *r*(*b*, *h*) into two smaller rectangles. The probability that it splits vertically is *p*(*b*, *h*), and if so then it splits at a uniform point along its base; otherwise it splits horizontally at a uniform point along its height. The functions *r* and *p* are given by

$$\begin{aligned} r(b,h) = \Big (\frac{1-\log b}{1-\log h}\Big ) \vee \Big (\frac{1-\log h}{1-\log b}\Big ) \end{aligned}$$

and

$$\begin{aligned} p(b,h) = \frac{1-\log h}{2(1-\log b)} \mathbbm {1}_{\{ b \le h \}} + \left( 1-\frac{1-\log b}{2(1-\log h)} \right) \mathbbm {1}_{\{ b > h\}}. \end{aligned}$$

It is easy to see that rectangles with either large base relative to their height, or large height relative to their base, split faster, and are more likely to split along their longer side. The appearance of $1-\log b$ and $1-\log h$, rather than *b* and *h*, is because splitting events have a multiplicative effect (e.g. a rectangle of height *h* that splits horizontally has two children with heights *Uh* and $(1-U)h$), and therefore taking logs of the dimensions gives us quantities that should behave roughly linearly over time. On the other hand, we stress again that our choices of *r* and *p* are far from the only possible or interesting ones, and our methods appear to be fairly robust: we give some guidance on how to adapt them to other sensible splitting rules in Sect. 1.8.

For a rectangle *v*, we denote its base by $B_v$ and its height by $H_v$. We let $X_v = -\log B_v$ and $Y_v = -\log H_v$. This is a standard transformation, used for example in [11], since rectangles’ sizes will decay exponentially with time and therefore $X_v$ and $Y_v$ are more useful parameterisations of size than $B_v$ and $H_v$. Under this transformation, our system has the following alternative description.

Begin with one particle at $(0,0)\in [0,\infty )^2$. Each particle, when at position (*x*, *y*) with $x,y \ge 0$, branches at rate

1 $$\begin{aligned} R(x,y)= \frac{x+1}{y+1} \mathbbm {1}_{\{ x \ge y\}} + \frac{y+1}{x+1} \mathbbm {1}_{\{ x < y\}}. \end{aligned}$$

At a branching event, the particle is replaced by two children: letting $\mathcal {U}$ be a uniform random variable on (0, 1), independent of everything else, then with probability

$$\begin{aligned} P(x,y) = \frac{y+1}{2(x+1)} \mathbbm {1}_{\{x \ge y\}} + \left( 1-\frac{x+1}{2(y+1)} \right) \mathbbm {1}_{\{x < y \}} \end{aligned}$$

the two children have positions $(x - \log \mathcal {U}, y)$ and $(x-\log (1-\mathcal {U}), y)$, and with probability $1-P(x,y)$ they have positions $(x, y - \log \mathcal {U})$ and $(x, y-\log (1-\mathcal {U}))$.

We let $R_X(x,y)=R(x,y)P(x,y)$ and $R_Y(x,y)=R(x,y)(1-P(x,y))$. Then $R_X$ and $R_Y$ denote the rates at which a particle at position (*x*, *y*) moves in the first spatial dimension, or the second, respectively. Note that $R_X(x,y)=1/2$ if $x\ge y$ and $R_X(x,y)=(y+1)/(x+1)-1/2$ if $x<y$, reflecting the shape-dependent behaviour of the fragments in the branching random walk, in the sense that the closer one of its components gets to zero, the faster a particle has a (positive) jump in that component, thereby moving away from the axis.

From now on, we mostly use the second description, and refer to particles and their positions, rather than rectangles and their sizes. As seen above, the two descriptions are entirely equivalent.

### Main theorem

Let *E* be the set of non-decreasing càdlàg functions $f:[0,1]\rightarrow \mathbb {R}$ with $f(0)=0$. For each $f\in E$, since *f* is non-decreasing, $f'(s)$ exists almost everywhere. Set $f'(s)= \infty$ if *f* is not differentiable at the point $s\in [0,1]$. For $t\in [0,1]$, write ${\tilde{f}}(t) = \int _0^t f'(s) \,\text {d}s$ and $\hat{f}(t) = f(t)-{\tilde{f}}(t)$.

Our main theorem aims to quantify how many particles have paths which, when rescaled appropriately, fall within a given subset of $E^2$. It is written in the style of a large deviations result, and uses large deviations techniques, although it is not actually a large deviations result since it is concerned with the almost-sure behaviour of the system rather than events of small probability.

Since we are interested in rescaled paths for large times, *R* and *P* are essentially governed by the ratios *x*/*y* and *y*/*x*. We define the functions $R^*:[0,\infty )^2\rightarrow [0,\infty ]$ and $P^*:[0,\infty )^2\rightarrow [0,1]$ by

$$\begin{aligned} R^*(x,y):= {\left\{ \begin{array}{ll} \frac{x}{y} \mathbbm {1}_{\{ x \ge y\}}+ \frac{y}{x} \mathbbm {1}_{\{ x<y\}} & \text { if } x>0 \text { or } y>0\\ 1 & \text { if } x=y=0\end{array}\right. } \end{aligned}$$

and

$$\begin{aligned} P^*(x,y):= {\left\{ \begin{array}{ll} \frac{y}{2 x}\mathbbm {1}_{ \{x \ge y\}} + \big (1-\frac{x}{2 y}\big ) \mathbbm {1}_{\{x < y\}} & \text { if } x>0 \text { or } y>0 \\ 1/2 & \text { if } x=y=0.\end{array}\right. } \end{aligned}$$

Although our splitting rule is described by the functions *R* and *P* (or equivalently *r* and *p*), which are continuous at 0, at large times the constant terms in those functions become insignificant and the behaviour when the system is rescaled appropriately is captured instead by $R^*$ and $P^*$. We let

$$\begin{aligned} R^*_X(x,y):= {\left\{ \begin{array}{ll} R^*(x,y)P^*(x,y) & \text { if } y>0 \\ 1/2 & \text { if } y=0\end{array}\right. } \end{aligned}$$

and

$$\begin{aligned} R^*_Y(x,y):= {\left\{ \begin{array}{ll} R^*(x,y)(1-P^*(x,y)) & \text { if } x>0 \\ 1/2 & \text { if } x=0. \end{array}\right. } \end{aligned}$$

Suppose that $f=(f_X,f_Y) \in E^2$ and $0\le a\le b\le 1$. We define several functionals of the path *f*. First,

$$\begin{aligned} I(f,a,b)= \int \limits _a^b \Big ( \sqrt{2 R^*_X(f(s))} - \sqrt{f_X'(s)} \Big )^2 \,\text {d}s + \int \limits _a^b \Big ( \sqrt{2 R^*_Y(f(s))} - \sqrt{f_Y'(s)} \Big )^2 \,\text {d}s \end{aligned}$$

represents the cost of following *f* if *f* is absolutely continuous; each of the two integrands corresponds to a Fenchel–Legendre transform of a Poisson number of independent exponential random variables (see also Sect. 1.5). Incorporating the cost of discontinuities in *f*, we define

$$\begin{aligned} J(f,a,b) = I(f,a,b) + {\hat{f}}_X(b) - {\hat{f}}_X(a) + {\hat{f}}_Y(b) - {\hat{f}}_Y(a), \end{aligned}$$

and then our expected growth rate is

$$\begin{aligned} K(f,a,b) = {\left\{ \begin{array}{ll} \int \limits _a^b R^*(f(s)) ds - J(f,a,b) &  \text { if } J(f,a,b)<\infty ;\\ -\infty &  \text { otherwise,}\end{array}\right. } \end{aligned}$$

which includes the growth due to the splitting rate along *f*, minus the cost of following *f*.

As with *f*, if *K*(*f*, 0, *t*) is not differentiable at *t* then write $\frac{d}{dt} K(f,0,t) = -\infty$. Define

$$\begin{aligned} K^+(f) = {\left\{ \begin{array}{ll} K(f,0,1) &  \text { if }\, K(f,0,s)\ge 0 \,\,\,\, \forall s\in [0,1];\\ -\infty &  \text { otherwise.}\end{array}\right. } \end{aligned}$$

and

$$\begin{aligned} K^-(f) = {\left\{ \begin{array}{ll} K(f,0,1) &  \text { if }\, K(f,0,s)> 0 \,\,\,\, \forall s\in [0,1] \text { and } \frac{d}{dt} K(f,0,t)|_{t=0} > 0;\\ -\infty &  \text { otherwise.}\end{array}\right. }. \end{aligned}$$

The functional *K*(*f*, 0, 1) will be our expected growth rate, in that the expected number of particles at time *T* whose paths, when rescaled by a factor *T*, are “near” *f* should look something like $\exp (K(f,0,1)T)$. However, the actual number of particles behaving in this way will only look like $\exp (K(f,0,1)T)$ if $K(f,0,s)\ge 0$ for all $s\in [0,1]$. If there exists $\theta \in [0,1]$ such that $K(f,0,\theta )<0$ then (with high probability) there will be no particles whose *T*-rescaled paths look like *f*, essentially because this point on *f* acts as a bottleneck; at time $\theta$, the expected number of rescaled particles near *f* looks like $\exp (K(f,0,\theta )T) \ll 1$, so by Markov’s inequality the actual number of particles must be zero with high probability, i.e. no particles follow near *f* up to time $\theta$, let alone up to time 1.

When $K(f,0,s) \ge 0$ for all $s\in [0,1]$ but $K(f,0,\theta )=0$ for some $\theta \in (0,1]$, the situation is delicate. In this case intuition would tell us that the (rescaled) branching process seen near *f* is effectively critical at time $\theta$. Similar logic holds at time 0 if $\frac{d}{dt} K(f,0,t)|_{t=0} = 0$. Our main theorem will therefore give an upper bound in terms of $K^+$ and a lower bound in terms of $K^-$.

In order to make this discussion precise we need to specify a topology on our space of functions $E^2$. Since the jumps in our process are exponentially distributed, it is possible for particles to make macroscopic jumps in the sense that their rescaled paths will not be continuous. In fact, it is possible for particles to make two (or more) macroscopic jumps in quick succession. This means that the usual Skorohod topology—the $J_1$ topology, generated by the Skorohod metric—is not suitable, as the rescaled set of paths that our particles take will not be compact in this topology. Instead we use the Lévy metric [31] on the set of increasing functions on [0, 1], which generates Skorohod’s $M_2$ topology. We will recall the definition shortly, but first point out that the Lévy metric has been extended to a metric on the set of càdlàg functions, known as the graph metric or Borovkov metric, which again generates the $M_2$ topology on this larger space. This metric was introduced in [15], and used for example in [32] and [36] to give a large deviations result for compound Poisson processes under only a weak moment Cramér condition, because of similar incompatibility with the $J_1$ topology.

The Lévy metric on *E* is defined by

2 $$\begin{aligned} d(f,g) = \inf \big \{r>0: f(x-r)-r< g(x) < f(x+r)+r \hspace{2mm} \forall x\in [-r,1+r]\big \}\nonumber \\ \end{aligned}$$

where *f*(*x*) is interpreted to equal *f*(0) for $x<0$ and *f*(1) for $x>1$, and similarly for *g*. The metric space (*E*, *d*) is complete and separable. In an abuse of notation, we will also write *d* to mean the product metric on $E^2$ defined by $d((f_X,f_Y),(g_X,g_Y))=\max \{d(f_X,g_X),d(f_Y,g_Y)\}$.

Take $T\ge 0$ and let $\mathcal {N}_T$ be the set of particles that are alive at time *T*. For $u\in \mathcal {N}_T$ and $t\le T$, let $Z_u(t) = (X_u(t),Y_u(t))$ be the position of the unique ancestor of *u* in $\mathcal {N}_t$. For $u\in \mathcal {N}_T$ and $s\in [0,1]$, write

$$\begin{aligned} Z_u^T(s) = Z_u(sT)/T; \end{aligned}$$

we call $(Z_u^T(s),\, s\in [0,1])$ the *T*-rescaled path of *u*. For $F\subset E^2$, define

$$\begin{aligned} N_T(F) = \#\{u\in \mathcal {N}_{T}: Z_u^T \in F\}, \end{aligned}$$

the number of particles at time *T* whose *T*-rescaled paths have remained within *F*. Throughout the article we use the convention that $\inf \emptyset = + \infty$ and $\sup \emptyset = - \infty$.

#### Theorem 1.1

If $F\subset E^2$ is closed, then

$$\begin{aligned} \limsup _{T\rightarrow \infty }\frac{1}{T}\log N_T(F) \le \sup _{f\in F} K^+(f) \,\,\,\, \text {almost surely,} \end{aligned}$$

and if $F\subset E^2$ is open, then

$$\begin{aligned} \liminf _{T\rightarrow \infty }\frac{1}{T}\log N_T(F) \ge \sup _{f\in F} K^-(f)\,\,\,\,\text {almost surely.} \end{aligned}$$

Although Theorem 1.1 gives an upper bound in terms of $K^+$ and a lower bound in terms of $K^-$, in practice we conjecture that any open ball around a function *f* such that $K^+(f)>0$ but $K^-(f)=-\infty$ must for any $\varepsilon >0$ contain *g* with $K^-(g)>K^+(f)-\varepsilon$. This would imply that for any open set $F\subset E^2$, we have $\sup _{f\in F} K^-(f) = \sup _{f\in F}K^+(f)$, and therefore the upper and lower bounds in our theorem match. Proving this is a lengthy exercise in analysis of no independent interest. This is exactly how the critical case was dealt with in [8], where the simpler form of the growth rate *K* made the analysis relatively straightforward.

### Heuristics

At a basic level, our theorem says that the number of particles whose *T*-rescaled paths remain close to a function *f* is roughly $\exp (K^+(f)T)$. The growth rate $K^+(f)$ consists of two parts: the growth of the population along the path, which is simply $\int _0^1 R^*(f(s)) ds$, and the cost of a typical particle following the path, which is *J*(*f*, 0, 1). However, if the cumulative cost is ever larger than the cumulative growth at any point along the path—that is, if *K*(*f*, 0, *s*) is ever negative—then particles are unable to follow *f* and therefore $K^+(f)=-\infty$.

The main strategy for the proof is to break time up into small intervals. On each small interval, we know roughly the location and gradient of *f* and the rate *R*(*f*(*s*)), so we can control both the growth and the cost of following *f*. We bound the largest and smallest values that *R*(*z*) can take when *z* is within a small ball around *f*(*s*), and use a coupling to trap a typical particle in our process between two compound Poisson processes that have jump rates corresponding to these maximum and minimum values of *R*(*z*). First and second moment bounds then allow us to translate the behaviour of this typical particle into estimates for the whole branching system.

As mentioned in the introduction, this simple explanation disguises a highly technically demanding proof. For example, the reason that we have to carry out our coupling on small time intervals is so that we can “reset” the distance between our three coupled processes at the end of each interval. In this way we can keep errors sufficiently small.

One of the difficulties that does not usually appear in work on branching structures is the behaviour at early times. A standard approach would be to let the system evolve freely for some time so that there is a large number of particles alive, and then treat each of these particles as a blank canvas, essentially starting its own copy of the original process, using the independence of these copies to improve the accuracy of initial bounds on a single population. We cannot do this, since our rate function *R* is not only spatially dependent, but once scaled by *T*, it becomes discontinuous at 0 (reflected in the discontinuity of $R^*$ at 0). Instead we are forced to use a discrete-time moment bound to show that there are many particles near one particular path—a straight line corresponding to rectangles that are roughly square—at small times, and then show that this collection of particles can “feed” a population at later times that is easier to control.

Another non-standard element in our proof is the appearance of the Lévy metric. As mentioned in Sect. 1.3, since our particles take jumps whose sizes are exponentially distributed, there are (many) particles whose *T*-rescaled paths are not continuous. Indeed, every particle branches at rate at least 1, so at time *tT* there are at least of order $e^{tT}$ particles, and the probability that one particle performs a jump larger than *aT*—which corresponds to size *a* in the rescaled picture—is $e^{-aT}$. Thus we expect to see many such jumps in the rescaled paths when $t>a$ (and since particles can branch faster than rate 1, we will in fact see such jumps significantly earlier). In order to bound the total number of particles from above, we therefore need to control particles whose paths are discontinuous; hence the appearance of the Lévy metric.

### Sample path large deviations

With our chosen scaling of the trajectories, the cost of a typical particle following a given path is a question about large deviations and the quantity *J*(*f*, 0, 1) plays the role of the rate function. We explain here how the analytical expression of *J*(*f*, 0, 1) can be related to the rate function for processes with constant rate.

Take a random variable *V* and let $(X(t), \ t \ge 0)$ be a compound Poisson process with rate *r* and jump distribution $-\log V$, satisfying the weak Cramér condition that

$$\begin{aligned} \lambda _0&= \inf \big \{\lambda : \mathbb {E}[e^{-\lambda \log V}]<\infty \big \} \in [-\infty , 0), \\ \lambda ^0&= \sup \big \{\lambda : \mathbb {E}[e^{-\lambda \log V}]<\infty \big \} \in (0,+\infty ]. \end{aligned}$$

This in particular implies that $\mathbb {E}[e^{-\lambda \log V}]<\infty$ for every $\lambda \in (\lambda _0, \lambda ^0)$. Define

$$\begin{aligned} \Lambda (r,x) = \sup _{\theta \in \mathbb {R}} \big \{\theta x + r - r\mathbb {E}[e^{-\theta \log V}]\big \}, \end{aligned}$$

which is the Fenchel–Legendre transform corresponding to a sum of Poisson(*r*) independent copies of the random variable $-\log V$.

Denote by *D* the set of càdlàg functions $f:[0,1]\rightarrow \mathbb {R}$ with $f(0)=0$. For $f \in D$, let ${\tilde{f}}$ be the its absolutely continuous part, and ${\hat{f}}$ its singular part. Write ${\hat{f}} = \hat{f}^+ - \hat{f}^-$ to be the unique representation of ${\hat{f}}$ as the difference of two non-decreasing functions. Then for $0 \le a < b \le 1$ let

$$\begin{aligned} I_r(f,a,b)= \int \limits _a^b\Lambda (r,{\tilde{f}}'(s)) ds \end{aligned}$$

and

$$\begin{aligned} J_r(f,a,b)= I_r(f,a,b) + \lambda ^0 ({\hat{f}}^+(b)-{\hat{f}}^+(a)) - \lambda _0 ({\hat{f}}^-(b)-{\hat{f}}^-(a)), \end{aligned}$$

with the convention that $0\cdot \infty = 0$. Mogul’skii [36], improving on a previous result by Lynch and Sethuraman [32], shows that the family of rescaled paths $\{X^T, \, T \ge 0 \}$, where $X^T(t)=X(tT)/T$, $t \in [0,1]$, satisfies a large deviation principle in $(D, M_2)$, with rate function $J_r$.

when the jump distribution $-\log V$ is exponentially distributed with mean 1, it is easy to check that

3 $$\begin{aligned} \Lambda (r,x) = (\sqrt{r}-\sqrt{x})^2 \end{aligned}$$

and (since ${\hat{f}} \equiv \hat{f}^+$ and $\lambda ^0=1$)

4 $$\begin{aligned} J_r(f,a,b) = \int \limits _a^b \left( \sqrt{r}-\sqrt{f'(s)} \right) ^2 ds +{\hat{f}}(b)-{\hat{f}}(a). \end{aligned}$$

Thus we can interpret the “cost term" *J*(*f*, *a*, *b*), $f \in E^2$ in Theorem 1.1 as the sum of the functionals $J_r(f_X,a,b)$ and $J_r(f_Y,a,b)$ for each one of the two components of *f*, where the rate *r* is replaced by the instantaneous rates $2R_X^*(f(s))$ and $2R_Y^*(f(s))$, respectively. The appearance of the factor 2 is due to the fact that a uniformly chosen particle at any time *t* will have split at roughly twice the usual rate; this phenomenon appears whenever one considers a continuous-time branching structure, and manifests through the change of measure introduced as part of the many-to-one formula, Lemma 2.2.

One might hope that our heuristic from Sect. 1.4 could be carried out by using the work of Lynch and Sethuraman [32] or Mogul’skii [36] directly, *without* needing to split time into small intervals. Given a path *f*, one could use a suitable coupling to bound the instantaneous jump rate for any particle following near *f* at each time *t* from above and below, and apply Mogul’skii’s large deviations result [36] to bound the probability that a particle follows such a path. First and second moment methods would then complete the proof. However this does not work; since our jump rate depends on our position, the accumulation of small errors is exacerbated and we quickly lose sufficient control of the upper and lower bounds. In order to keep the errors small enough, it is therefore necessary to work on short time intervals, allowing us to “reset” the error in our coupling to zero at the start of each interval. We also require specific error bounds; although the estimates we use require only standard methods, we were not able to find the specific forms that we need in standard references for either large deviations or Poisson processes. We therefore give standalone proofs of suitable error bounds on compound Poisson processes in Lemmas 6.1 and 6.2, using well-known large deviations methods.

### Growth rate in expectation

A relatively minor modification of our proof of Theorem 1.1 would yield that the growth rate in expectation is given by *K*(*f*, 0, 1) (see discussion after the definition of $K^+(f)$); to be more precise, if $F\subset E^2$ is closed then

5 $$\begin{aligned} \limsup _{T\rightarrow \infty }\frac{1}{T}\log \mathbb {E}[N_T(F)] \le \sup _{f\in F} K(f,0,1) \end{aligned}$$

and if $F\subset E^2$ is open then

6 $$\begin{aligned} \liminf _{T\rightarrow \infty }\frac{1}{T}\log \mathbb {E}[N_T(F)] \ge \sup _{f\in F} K(f,0,1). \end{aligned}$$

In particular one may note that there are many sets *F* such that the expected number of particles whose rescaled paths fall within *F* is exponentially large, since $\sup _{f\in F^\circ }K(f,0,1)>0$, and yet almost surely no particles have rescaled paths that fall within *F*, since $\sup _{f\in {\bar{F}}} K^+(f) = -\infty$.

In order to keep this article to a manageable length, we do not include full proofs of (5) and (6) here, although they are significantly simpler than the proofs of the upper and lower bounds in Theorem 1.1. We will sketch the main points of the arguments in Sects. 2.3 and 3, shortly after the respective proofs of the upper and lower bounds in Theorem 1.1.

### Optimal paths for growth rates at given positions

Theorem 1.1 tells us how many particles fall within a given set of sample paths at large times. If we instead want to know how many particles there are at a certain position at a large time (corresponding to the number of rectangles with a certain shape), we need to optimise the functional *K*(*f*, 0, 1) over all paths *f* with a given endpoint. This is a purely analytic problem, and we have no reason to suppose that a closed-form solution exists; a similar problem in a significantly simpler one-dimensional setting in [8] was already non-trivial. By symmetry we may assume without loss of generality that $f_Y(s)\ge f_X(s)$ for all *s*, and one may also show that any optimal paths are continuous. It is straightforward to derive a pair of Euler-Lagrange equations that any smooth function *f* optimising *K*(*f*, 0, 1), and satisfying $f_Y(s)\ge f_X(s)$ for all *s*, must satisfy away from 0:

$$\begin{aligned} \frac{f''_Y(s)}{2f'_Y(s)^{3/2}} - \frac{1}{f_X(s)} + \frac{\sqrt{2f'_X(s)}}{f_X(s)(f_Y(s)/f_X(s)-1/2)^{1/2}} = 0 \end{aligned}$$

and

$$\begin{aligned}  &   f_Y(s)\sqrt{2f'_X(s)}\left( \frac{f_Y(s)}{f_X(s)}-\frac{1}{2}\right) ^{1/2} + \frac{f_X(s)^2 f''_X(s)}{f'_X(s)}\left( \frac{f_Y(s)}{f_X(s)}-\frac{1}{2}\right) \\  &   - f_X(s)f'_Y(s) - f'_X(s)f_Y(s)=0. \end{aligned}$$

If the desired endpoint $z=(x,y)$ (with $y\ge x$) satisfies $y = 2x^2 + x/2$ for $x\ge 1/4$, then the function satisfying the above two equations is the straight line, $f(s) = (xs, ys)$, and

$$\begin{aligned} K(f,0,s) = \left( \frac{x}{2}-2x^2-\frac{1}{2} + 2x^{1/2}(2x+1/2)^{1/2}\right) s. \end{aligned}$$

In this case $K(f,0,s)>0$ for all $s\in (0,1]$ whenever the above function of *x* is strictly positive, corresponding roughly to $x<1.6149$; and *K*(*f*, 0, 1) is maximised (amongst this reduced class of straight lines) when $x\approx 0.83814$, in which case $K(f,0,1)\approx 1.21524$. One may calculate these values exactly, but the approximate forms are more easily stated.

These are the only cases for which the optimal paths are straight lines. Indeed, one can check—by writing *f* as a formal power series—that any function *f* satisfying the above equations must satisfy $f'_Y(0)=2f'_X(0)^2 + f'_X(0)/2$.

**Fig. 3:**
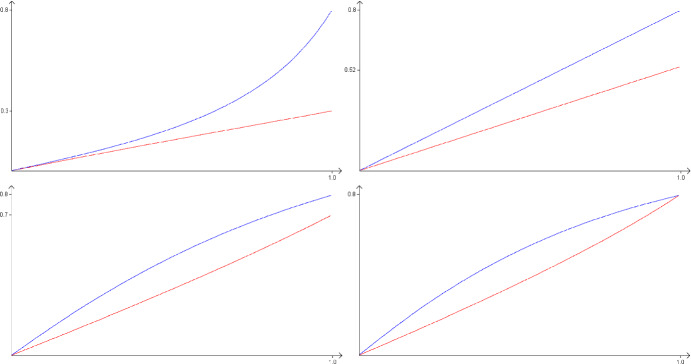
Different shapes of the optimal function pairs for different endpoints *z*. The top (blue) curve shows $f_Y(s)$ and the lower (red) curve is $f_X(s)$. The corresponding approximate values of *K*(*f*, 0, 1) are 0.616, 1.00, 1.11 and 1.12, for $x=0.3$, 0.52, 0.7 and 0.8 respectively, with $y=0.8$. In all cases $K(f,0,s)>0$ for all $s\in (0,1]$

**Fig. 4:**
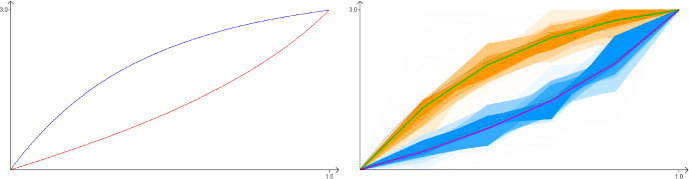
When $z=(3,3)$, the *f* given by solving the Euler-Lagrange equations (shown on the left) satisfies $K(f,0,s)<0$ for some $s\in (0,1)$, and therefore this cannot be the optimal function since $K^+(f)=-\infty$, even though $K(f,0,1)\approx 0.959$. In the right-hand image we did a Metropolis-Hastings search for the optimal function among piecewise linear functions with 5 equal timesteps, which suggested curves that are less steep for small values of *s*, giving $K(f,0,1)\approx 0.935$. A brute force search amongst polynomials of degree at most 3 gave a similar result, with $K(f,0,1)\approx 0.937$ when $f_X(s) = 1.634s + 0.2s^2 + 1.166s^3$ and $f_Y(s) = 6s-3.7s^2+0.7s^3$

The optimal paths will not always satisfy the above Euler-Lagrange equations, since we need to optimise *K*(*f*, 0, 1) over the restricted class of functions satisfying $K(f,0,s)\ge 0$ for all $s\in (0,1]$. For example, in [8], naively solving the Euler-Lagrange equations gave paths that showed negative initial growth, whereas the actual optimal paths initially followed the trajectory of the maximal particle—which showed zero growth—before breaking off at some time $t_z\in (0,1)$, depending on the desired endpoint *z*, and then following the Euler-Lagrange equations on the smaller time interval $(t_z,1]$. Numerical methods suggest (see Figs. 3 and 4) that for many values of *z*, the Euler-Lagrange equations do give the optimal function *f*, but there do exist values of *z* for which this is not the case.

Numerical methods also indicate that the optimal value of *z*, corresponding to the most abundant size and shape of rectangle at large times, is $z\approx (1.48,2.14)$, with $K(f,0,1)\approx 1.44$.

### Generalising our results to incorporate other splitting behaviour

As mentioned in Sect. 1.1, we believe that our methods should be adaptable to a variety of other splitting rules. We attempt here to give some further details on how this would work.

There are three main parameters in our model: the splitting rate *R*(*z*) and the directional probability *P*(*z*), each defined for any $z\in [0,\infty )^2$ as functions of the negative logarithm of the base and height of a rectangle, and the splitting rule, which in our case is uniform across the side length in the chosen direction.

**Scaling regime**

We assume the existence of rescaled functions $R^*$ and $P^*$ such that $R(Tz)\rightarrow R^*(z)$ and $P(Tz)\rightarrow P^*(z)$ as $T\rightarrow \infty$ for any $z\in [0,\infty )^2$. The rescaled functions are then necessarily scale-invariant, i.e. $R^*(az)=R^*(z)$ and $P^*(az)=P^*(z)$ for any $z\in [0,\infty )^2$ and $a>0$, which facilitates our use of large deviations techniques.

It would be interesting to consider other scaling regimes. For example, another natural question would be to consider the case $r(b,h) = \frac{b}{h}\vee \frac{h}{b}$, without taking logs, in which case we would have $R(x,y) = e^{x-y}\vee e^{y-x}$. In this case, we would observe a much smaller range of shapes, with all rectangles forced to stay much closer to square in shape; it would be interesting to investigate how large the fluctuations from square could be. Another possibility would be to incorporate the area of rectangles into *r*(*b*, *h*), so that smaller rectangles branch faster or slower. This would lead to very different behaviour; see e.g. Bertoin’s work on self-similar fragmentations [10], but also the work of Cesana and Hambly [21] and of Broutin, Neininger and Sulzbach [19].

**Symmetry of the directional probability** *P*

In Lemma 4.1, we use that $P(x,1)\rightarrow 0$ as $x\rightarrow \infty$ and that $P(1,y)\rightarrow 1$ as $y\rightarrow \infty$; in other words, that long, thin rectangles (in either co-ordinate) are overwhelmingly likely to split along their longest side. Then the key ingredient in Lemma 5.1 is that rectangles are always more likely to split along their longer side than their shorter side, i.e. that $P(x,y)\le 1/2$ for $x\ge y$ and $P(x,y)\ge 1/2$ for $x\le y$. Neither of these assumptions should be entirely necessary, but without them a substantially different approach to that taken in Sect. 5 would be required to ensure the growth of the population at small times.

**Properties of the splitting rate** *R*

- Although our splitting rate is unbounded, towards the end of the proof of Lemma 4.1 we use the fact that if *x* and *y* are within a fixed ratio of each other, then *R*(*x*, *y*) is bounded above. More precisely, we use that for any $M>0$, there exists a finite constant *C* such that $R(x,y)\le C$ whenever $x\le My$ and $y\le Mx$.
- We also use that *R*(*z*) is bounded away from zero in Lemma 4.1. This is not necessary; the proof of Lemma 4.1 could easily be changed to accommodate a condition similar to the upper bound on *R* mentioned above, namely that for any $M>0$, there exists a constant $c>0$ such that $R(x,y)\ge c$ whenever $x\le My$ and $y\le Mx$.
- In Lemma 5.3 we use that $R^*$ is continuous at (1, 1). Again this does not appear to be entirely necessary, but relaxing it would require some extra work.

**Uniform splitting**

There is no reason that rectangles must split uniformly along whichever side is chosen. In fact, the uniform split leads to exponentially distributed jumps, and the exponential distribution does not satisfy the strong Cramér condition, leading to the possibility of macroscopic jumps in the rescaled picture, which make our model somewhat more technically demanding to study. If we more generally take *V* to be a random variable taking values in (0, 1) and representing the distribution of the split along a side of length 1, we must assume that $-\log V$ satisfies the weak Cramér condition (see Sect. 1.5). In Lemma 5.1 we use the second, fourth and sixth moments of the exponential distribution, but this could easily be changed to accommodate other distributions with finite sixth moment.

**Behaviour at small times**

The main barrier preventing us from significantly generalising our theorem is the need to demonstrate that there are many particles at small times moving in all “positive growth” directions. We use Proposition 3.4 to do this, by virtue of a hands-on construction of paths leading from the lead diagonal to lines of other gradients in Sect. 5.2. We used specific properties of the function $\kappa (\lambda ,\mu )$ for $\lambda ,\mu \in (0,\infty )$, which in a more general picture would become

$$\begin{aligned} \kappa (\lambda ,\mu ) = R^*(\lambda ,\mu ) - \Lambda (2R^*_X(\lambda ,\mu ),\lambda ) - \Lambda (2R^*_Y(\lambda ,\mu ),\mu ), \end{aligned}$$

where as usual $R^*_X(z) = R^*(z)P^*(z)$ and $R^*_Y(z) = R^*(z)(1-P^*(z))$. We expect that similar calculations could be carried out for other choices of parameters *R*, *P* and *V*, but a general argument that works for a broad collection of choices of *R* and *P* appears difficult. One could instead impose an abstract condition along the lines of “the set of $(\lambda ,\mu )$ such that $\kappa (\lambda ,\mu )>0$ must be simply connected and totally bounded”.

**A more general form of Theorem** 1.1

We expect that, subject to conditions as outlined above, one could prove a more general form of Theorem 1.1 for other choices of *R*, *P* and the splitting rule. The statement of Theorem 1.1 itself would remain unchanged, but would feature altered functionals

$$\begin{aligned} I(f,a,b) = \int \limits _a^b \Lambda \Big (2R^*_X(f(s)),f'_X(s)\Big )\,\text {d}s + \int \limits _a^b \Lambda \Big (2R^*_Y(f(s)),f'_Y(s)\Big )\,\text {d}s \end{aligned}$$

and

$$\begin{aligned} J(f,a,b) = I(f,a,b) + \left( {\hat{f}}_X(b) - {\hat{f}}_X(a) + {\hat{f}}_Y(b) - {\hat{f}}_Y(a)\right) \lambda ^0, \end{aligned}$$

where $\lambda ^0 = \sup \{\lambda : \mathbb {E}[e^{-\lambda \log V}]<\infty \} > 0$ and the rate function $\Lambda$ were defined in Sect. 1.5.

### Related work

A similar model to ours has been considered by Cesana and Hambly [21], and Ball, Cesana and Hambly [5], motivated by applications to a martensitic phase transition observed in a class of elastic crystals. They consider different splitting rule variants, and work in both two and three dimensions; but rectangles always split at rates that depend only on their area, with a constant probability *p* (or $1-p$) of splitting horizontally (or vertically), ensuring that their models, suitably transformed, fit into the framework of generalised branching random walks. This allows them to apply a slightly modified version of a theorem of Biggins [14]. They give almost sure growth rates for the number of fragments of different shapes, and, motivated by predictions from the physics literature, they study the lengths of the horizontal “interfaces” between fragments, obtaining that in certain cases the total number of interfaces larger than *x* behaves like a random variable multiplied by an explicit power of *x*. In summary, although their model is on the surface similar to our own, the regime that they consider leads to dramatically different behaviour, and they are interested in measuring different quantities. Their techniques are therefore completely distinct from our own. Our results could not be obtained by their methods; while perhaps some of their results—e.g. [21, Theorem 1.2]—could be obtained using methods similar to ours, this would be a very inefficient approach, and other results, e.g. the power law obtained in [21, Theorem 1.1], certainly require different techniques.

Another model of spatial fragmentation is the work on *quadtrees*, e.g. by Broutin, Neininger and Sulzbach [19], in which a sequence of points $p_1,p_2,\ldots \in [0,1]^2$ are used to split the unit square as follows. The unit square is divided into four regions with the first point $p_1$ at their intersection; $p_2$ lies within one of those regions, which is then divided further into four sub-regions with $p_2$ at their intersection; and so on. They assume that the $p_1,p_2,\ldots$ form an independent sequence of uniform random variables on $[0,1]^2$, and investigate the number of regions after *n* splits that intersect a vertical line at location $s\in [0,1]$, interpreted as the cost of a partial match query.

Our methods are related to those of Berestycki et al. [8] on a branching Brownian motion (BBM) with inhomogeneous breeding potential. In that paper, the authors considered a BBM in $\mathbb {R}$ where a particle at position *z* branched at infinitesimal rate $|z|^p$, for $p\in [0,2)$. Their main result was roughly analogous to ours, giving almost sure growth rates along paths, and they also gave growth rates in expectation analogous to (5) and (6). They analysed their growth rates in some detail, giving implicit equations for the optimal paths and the location of the bulk of the population (which became explicit in the cases $p=0$ and $p=1$). This was a difficult analytic task even for the relatively simple, monotone growth rate seen in [8]. Our growth rate $K^+$ is much more complex and the optimal paths more difficult to study analytically; we give some partial results in Sect. 1.7.

There are three main difficulties in our model relative to that in [8]. Firstly, in the BBM, all particles move as standard Brownian motions, independent of their location and their branching rate, whereas in our model particles jump and branch simultaneously. Indeed, it is worth noting that if the branching Brownian motion in [8] were replaced by an analogous branching random walk, then if we started with one particle at 0, the initial particle would never branch or move; whereas if we started with a particle at any other site, then even with bounded jump sizes, the collection of particles would colonise space dramatically faster than the BBM (subject to the initial population not returning to 0 quickly), since a particle branching at rate $|z|^p$ would also be moving at rate $|z|^p$. This highlights the challenge of controlling the dependencies between particles’ positions and the growth of the population in our model.

On top of this initial difference, our branching rate *R*(*z*) is much more difficult to control than the smooth, symmetric, monotone (on each half-space) function $|z|^p$. And thirdly, our particles are able to make large jumps, meaning that standard large deviations apparatus is more difficult to apply, and we must use a non-standard topology.

Branching processes with time-inhomogeneous potential have also yielded interesting results, for example the model of Fang and Zeitouni [24, 25], with further work by Maillard and Zeitouni [33] and Nolen, Roquejoffre and Ryzhik [37], and generalisations by Fang [23] and Mallein [34]. See also work of Addario-Berry and Maillard [1] on continuous random energy models.

Roberts and Schweinsberg [38] also consider branching Brownian motion in an inhomogeneous potential, this time with a biological application in mind, where the position of a particle represents its fitness and fitter individuals branch more quickly. They used tools from [8] to give a heuristic explanation of some of their results, but used a more precise truncation argument for their proofs, based on techniques from [6] and [7].

For homogeneous spatial branching processes, obtaining a full picture of the spread of the population has been a subject of interest for more than 45 years. To give just a few highlights, the position of the extremal particle in BBM was studied by McKean [35] and Bramson [16, 17], with more detailed recent studies on the behaviour near the extremal particle by Aïdékon *et. al.* [3] and Arguin, Bovier and Kistler [4]. For non-lattice branching random walks, Aïdékon [2] proved convergence in law for the re-centered position of the extremal particle under fairly weak conditions. Bramson, Ding and Zeitouni [18] gave a shorter proof using a second moment method and indicated that it should be possible to adapt their proof to branching random walks that take values on a lattice.

### Layout of the article

We begin, in Sects. 2 and 3, with outlines of the proofs of the upper and lower bounds in Theorem 1.1 respectively. In these sections we state several results that are needed for the proof of the main theorem without proving them. The proofs of these intermediate results are then given in later sections.

In Sect. 4, we give a full construction of our system in terms of a marked binary tree. This discrete setting is useful for decoupling some of the dependency structure between the jump times and jump sizes, and allows us to show that particles remain within some compact set with high probability, which will be an important ingredient, especially for the upper bound in Theorem 1.1.

In Sect. 5 we aim to control the system at small times, which is a difficult task partly due to the discontinuity of $R^*$ at 0. We again use the discrete setup described in Sect. 4, and use moment estimates that take advantage of the fact that our particles prefer to split along their longest edge. This work is used for the proof of the lower bound in Theorem 1.1.

One of the main tools in our proof is a coupling between compound Poisson processes, which we describe in Sect. 6 and then apply to give upper and lower bounds on the probability that a typical particle remains near a given function.

In Sect. 7 we put many of the previous results together, move from lattice times to continuous time, and complete the final details of the proof of the upper bound in Theorem 1.1.

In Appendix A we give deterministic bounds that relate the maximum and minimum of *R* on small balls to the value of $R^*$ at the centre of the ball, and therefore allow us to link the probabilistic estimates obtained in Sect. 6 to our growth rate *K*.

An elementary but somewhat intricate bound on compound Poisson processes is required in Sect. 6, and we prove this in Appendix B.

Finally, in Appendix C we carry out some technical work, ensuring that our state space and our growth rate behave sensibly.

## Proof outline for the upper bound in Theorem 1.1

Since the proof of Theorem 1.1 is rather long, we break it into upper and lower bounds. In this section we state a series of results that together enable us to complete the upper bound. We will then prove those results in later sections.

### Three probabilistic ingredients

The first step in our proof of the upper bound in Theorem 1.1 is to rule out certain paths that it is difficult for particles to follow, thereby reducing the paths of interest to a compact set. We define, for $M>1$,

$$\begin{aligned} G_M = \left\{ f \in E: s/M \le f(s) \le Ms \,\,\forall s\in [0,1]\right\} \subset E. \end{aligned}$$

If $f\in G_M^2$ then we say that *f* is “*M*-good”. We note that if *f* is *M*-good then $R^*_X(f(s))\le M^2$ for all $s\in [0,1]$ and similarly for $R^*_Y$.

We would like to say that the rescaled paths of all particles fall within $G_M^2$ for sufficiently large *M*, but there is a complication near $s=0$ in that particles will not jump immediately and therefore their paths will fall, however briefly, outside $G_M^2$. Expanding $G_M^2$ by any fixed distance $\varepsilon >0$ would not allow us to control the jump rate sufficiently well, and we instead define, for $M>0$ and $T>1$,

$$\begin{aligned} G_{M,T}:= \left\{ f \in E: s/M - 2T^{-2/3} \le f(s) \le M(s+2T^{-2/3}) \,\,\forall s\in [0,1]\right\} . \end{aligned}$$

If $f\in G_{M,T}^2$ then we say that *f* is “(*M*, *T*)-good”. We can then show that for large *M* all particles are (*M*, *T*)-good with high probability as $T\rightarrow \infty$. We note here that the choice of $-2/3$ is not essential; we could choose any power of *T* in $(-1,-1/2)$.

#### Lemma 2.1

There exist $M_0>1$ and $\delta _0>0$ such that for any sufficiently large *T*,

$$\begin{aligned} \mathbb {P}\big (\exists v\in \mathcal {N}_T: Z_v^T \not \in G_{M_0,T}^2\big ) \le e^{-\delta _0 T^{1/3}}. \end{aligned}$$

We will prove this lemma in Sect. 4.

Next we give a version of the many-to-one formula, which translates expectations over all particles in our system into calculations involving just one particle. For $z_0\in [0,\infty )^2$, write $\mathbb {Q}_{z_0}$ for a probability measure under which $\xi _t$ is a Markov process living in $\mathbb {R}^2$, such that

- $$\xi _0=z_0$$;
- when the process is in state *z*, jumps occur at rate 2*R*(*z*);
- when a jump occurs from state *z*, it is of the form $(\mathbbm {e},0)$ with probability *P*(*z*) and $(0,\mathbbm {e})$ with probability $1-P(z)$, where $\mathbbm {e}$ is an independent exponentially-distributed random variable with parameter 1.

In other words, the process under $\mathbb {Q}_{z_0}$ behaves like a single particle under $\mathbb {P}_{z_0}$ except that it jumps at twice the rate. We write $\mathbb {Q}_{z_0}$ both for the measure and for its corresponding expectation operator. We will often take $z_0=0$, and in this case we sometimes write $\mathbb {Q}$ rather than $\mathbb {Q}_0$.

The measure $\mathbb {Q}_{z_0}$ described above is precisely the measure $\mathbb {Q}_{z_0}^1$ that appears in [27]. The following result is [27, Lemma 1] in the case of our model when $k=1$.

#### Lemma 2.2

(Many-to-one, Lemma 1 of [27] with $k=1$) Suppose that $z\in \mathbb {R}^2$ and $t\ge 0$. For any measurable function $f:\mathbb {R}^2\rightarrow \mathbb {R}$,

$$\begin{aligned} \mathbb {E}_z \left[ \sum _{u \in {\mathcal {N}}_t} f(Z_u(t)) \right] = \mathbb {Q}_z \left[ f(\xi _t) e^{\int \limits _0^t R(\xi _s) ds} \right] . \end{aligned}$$

This, combined with Markov’s inequality, allows us to give upper bounds on the number of particles whose paths fall within a particular set *F* simply by bounding *R*(*f*) over all $f\in F$ and then estimating the probability that $\xi$ falls within *F*. Estimating this probability will be our next task, but our estimates will not be exactly in terms of the quantities $R_X^*$ and $R_Y^*$ seen in Theorem 1.1. Instead they will involve taking the worst and best possible values of $R_X$ and $R_Y$ over small balls about appropriately chosen functions, during a small time interval. We will need several definitions. The reader may like to think of $F=B(f,\varepsilon )$ for some suitably nice function *f* and small $\varepsilon >0$.

For a non-empty interval $I\subset [0,1]$, $F\subset E^2$ and $T\ge 1$, define

$$\begin{aligned} R_X^-(I,F,T) = \inf \big \{R_X(Tg(s)): s\in I,\, g\in F\big \} \end{aligned}$$

and

$$\begin{aligned} R_X^+(I,F,T) = \sup \big \{R_X(Tg(s)): s\in I,\, g\in F\big \}, \end{aligned}$$

and similarly for $R_Y^-(I,F,T)$ and $R_Y^+(I,F,T)$. These correspond to the maximal and minimal possible jump rates over the interval *I* for particles whose *T*-rescaled paths fall within *F*. For $s\in [0,1]$, we also let

$$\begin{aligned} x^-(s,F) = \inf \{g_X(s): g\in F\}, \hspace{4mm} x^+(s,F) = \sup \{g_X(s): g\in F\}, \end{aligned}$$

and similarly for $y^-(s,F)$ and $y^+(s,F)$.

Writing |*I*| for the length of *I* and $I^-$ and $I^+$ for the infimum and supremum of *I* respectively, say that we are in the “$X-$ case” if $2R^-_X(I,F,T)|I| > x^+(I^+,F) - x^-(I^-,F)$; and in the “$X+$ case” if $x^-(I^+,F) - x^+(I^-,F) > 2R^+_X(I,F,T)|I|$. Note that these two cases are mutually exclusive, and roughly correspond to whether the drift of the process on the interval *I* multiplied by the length of the interval is larger or smaller than the distance we would like it to travel. Note also that it is possible to be in neither case. Define

$$\begin{aligned}  &   {\mathcal {E}}^+_X(I,F,T) \\  &   \quad = {\left\{ \begin{array}{ll} \Big (\sqrt{2R^-_X(I,F,T)|I|}-\sqrt{x^+(I^+,F) - x^-(I^-,F)}\Big )^2 &  \text {in the } X- \text { case;}\\ \Big (\sqrt{2R^+_X(I,F,T)|I|}-\sqrt{x^-(I^+,F) - x^+(I^-,F)}\Big )^2 &  \text {in the } X+ \text { case;}\\ 0 &  \text {otherwise.}\end{array}\right. } \end{aligned}$$

Similarly define ${\mathcal {E}}^+_Y(I,F,T)$. We note that for one function $f\in E^2$, the quantity ${\mathcal {E}}^+_X([a,b],\{f\},T) + {\mathcal {E}}^+_Y([a,b],\{f\},T)$ should be an approximation to—but a little bit bigger than—the functional *I*(*f*, *a*, *b*) seen in Sect. 1. We similarly define a quantity that should be an approximation to *I*(*f*, *a*, *b*) from below, namely

$$\begin{aligned}  &   {\mathcal {E}}^-_X(I,F,T) \\  &   \quad = {\left\{ \begin{array}{ll} \Big (\sqrt{2R^+_X(I,F,T)|I|}-\sqrt{x^-(I^+,F) - x^+(I^-,F)}\Big )^2 &  \text {in the } X- \text { case;}\\ \Big (\sqrt{2R^-_X(I,F,T)|I|}-\sqrt{x^+(I^+,F) - x^-(I^-,F)}\Big )^2 &  \text {in the } X+ \text { case;}\\ 0 &  \text {otherwise.}\end{array}\right. } \end{aligned}$$

Write $\Vert z_1-z_2\Vert = \max \{|x_1-x_2|,|y_1-y_2|\}$ when $z_i = (x_i,y_i)\in \mathbb {R}^2$ for $i=1,2$. To help us to break the time interval [0, 1] into smaller chunks, for $n\in \mathbb {N}$ we define a new metric $\Delta _n$ on $E^2$ by

$$\begin{aligned} \Delta _{n}(f,g):= \max \left\{ \Vert f(i/n)- g(i/n)\Vert : i=0,\dots , n\right\} . \end{aligned}$$

For $T>1$, $n\in \mathbb {N}$, $M>1$ and $f\in E^2$, we let

$$\begin{aligned} \Gamma _{M,T}(f,n) = B_{\Delta _n}(f,1/n^2)\cap B_d(f,1/n)\cap G_{M,T}^2 \end{aligned}$$

and for $j\in \{0,1,\ldots ,n-1\}$, let $I_j=[j/n,(j+1)/n]$.

We also need to extend our rescaling notation to $\xi$ in the natural way. Write $\xi ^T$ for the rescaled process $(\xi (sT)/T,\,s\in [0,1])$, and for $I\subset [0,1]$ write $\xi ^T|_{I}$ for the restriction to *I*, $(\xi (sT)/T,\,s\in I)$. If $F\subset E^2$ and a function *f* is defined on a subinterval *I* of [0, 1]—for example $\xi ^T |_{[0,\theta ]}$ with $I=[0,\theta ]$—then say that $f\in F|_I$ if there exists $g\in F$ such that $f(s)=g(s)$ for all $s\in I$.

#### Proposition 2.3

Suppose that $f\in E^2$, $n\in \mathbb {N}$, $T>1$ and $M>1$. Then for any $\theta \in (0,1]$, $i\in \{0,1,\ldots ,\lfloor \theta n\rfloor -1\}$, and *z* such that $\Vert z-f(i/n)\Vert <1/n^2$,

$$\begin{aligned}  &   \mathbb {Q}\big (\xi ^T|_{[i/n,\theta ]}\in \Gamma _{M,T}(f,n)\big |_{[i/n,\theta ]}\,\big |\,\xi ^T_{i/n}=z\big )\\  &   \le \exp \bigg (-T\sum _{j=i}^{\lfloor \theta n\rfloor -1} \big (\mathcal E^+_X(I_j,\Gamma _{M,T}(f,n),T) + \mathcal E^+_Y(I_j,\Gamma _{M,T}(f,n),T)\big )\bigg ). \end{aligned}$$

The proof of Proposition 2.3 will be the most interesting part of this paper, and involves coupling the process $\xi$ with two other processes, which—as long as $\xi$ remains within $\Gamma _{M,T}(f,n)$—will stay above and below $\xi$ respectively. We carry out this part of the argument in Sect. 6.

### Deterministic bounds

The three results Lemmas 2.1, 2.2 and Proposition 2.3 form the main part of our argument, and contain all of the probability required for the upper bound in Theorem 1.1.

Our next task is to translate the quantities ${\mathcal {E}}^+_X$ and ${\mathcal {E}}^+_Y$ into the more palatable rate functions seen in our main theorem. The deterministic arguments required are not particularly interesting. It will sometimes be useful to note that if $\int _a^b R^*(f(s)) ds<\infty$, then *K*(*f*, *a*, *b*) has the following alternative representation:

7 $$\begin{aligned} K(f,a,b)  &   = -\int \limits _a^b R^*(f(s)) ds + 2\sqrt{2}\int \limits _a^b \sqrt{R^*_X(f(s))f'_X(s)} ds \nonumber \\  &   \quad + 2\sqrt{2}\int \limits _a^b \sqrt{R^*_Y(f(s))f'_Y(s)} ds - f_X(b) + f_X(a) - f_Y(b) + f_Y(a).\nonumber \\ \end{aligned}$$

This can be seen by expanding out the quadratic terms in the definition of *I*(*f*, *a*, *b*) and simplifying.

Let ${{\,\textrm{PL}\,}}_n$ be the subset of functions in *E* that are linear on each interval $[i/n,(i+1)/n]$ for all $i=0,\ldots ,n-1$ and continuous on [0, 1].

#### Proposition 2.4

Suppose that $\theta \in (0,1]$, $M>1$, $n\ge 2\,M$ and $f\in {{\,\textrm{PL}\,}}_n^2\cap G_M^2$. Then for any $k\in \{\lceil \sqrt{n}\rceil ,\ldots ,\lfloor \theta n\rfloor -1\}$,

$$\begin{aligned}  &   \sum _{j=k}^{\lfloor \theta n\rfloor -1} \mathcal E^+_X(I_j,\Gamma _{M,T}(f,n),T)\\  &   \ge \int \limits _{k/n}^{\lfloor \theta n\rfloor /n} \Big (\sqrt{2R_X^*(f(s))} - \sqrt{f'_X(s)}\Big )^2 ds - O\Big (\frac{M^4}{n^{1/4}}+\frac{M^3n}{T^{1/2}}\Big ). \end{aligned}$$

We do not aim to give best possible bounds on the error term. Similarly for the sum on the left-hand side, small values of *j* give rise to larger errors, so there should be some cut-off, but the choice of $\lceil \sqrt{n}\rceil$ is convenient rather than optimal. We will prove Proposition 2.4 in Appendix A.1.

We will also need the following bound to control the $\exp (\int _0^t R(\xi _s) ds)$ term seen in Lemma 2.2.

#### Lemma 2.5

Suppose that $\theta \in (0,1]$, $M>1$, $n\ge 2M$, $T^{2/3}\ge 3Mn^{1/2}$, $f\in G_M^2$ and $g\in \Gamma _{M,T}(f,n)$. Then

$$\begin{aligned} \int \limits _0^\theta R(Tg(s)) ds \le \int \limits _0^{\lfloor \theta n\rfloor /n} R^*(f(s))ds + \eta (M,n,T) \end{aligned}$$

and for any $k\in \{\lceil \sqrt{n}\rceil , \lceil \sqrt{n}\rceil +1, \ldots , \lfloor \theta n\rfloor \}$,

$$\begin{aligned} \int \limits _{k/n}^{\lfloor \theta n\rfloor /n} R^*(f(s))ds - \eta (M,n,T)&\le \int \limits _{k/n}^\theta R(Tg(s)) ds \\&\le \int \limits _{k/n}^{\lfloor \theta n\rfloor /n} R^*(f(s))ds + \eta (M,n,T) \end{aligned}$$

where

$$\begin{aligned} \eta (M,n,T) = O\Big (\frac{M^4}{n^{1/2}} + \frac{M^3n}{T^{1/3}}\Big ). \end{aligned}$$

This result will be proved in Appendix A.2. Again we make little effort to make $\eta (M,n,T)$ the best possible bound.

### Completing the proof of the upper bound in Theorem 1.1

Recall that if $F\subset E^2$, and $g:[0,\theta ]\rightarrow \mathbb {R}^2$, we say that $g\in F|_{[0,\theta ]}$ if there exists a function $h\in F$ such that $h(u)=g(u)$ for all $u\in [0,\theta ]$. We also generalise our rescaling notation slightly: for $t\in [0,T]$, $v\in \mathcal {N}_{t}$ and $s\in [0,t/T]$, write

$$\begin{aligned} Z_v^T(s) = Z_v(sT)/T; \end{aligned}$$

again we call $(Z_v^T(s), s\in [0,t/T])$ the *T*-rescaled path of *v* (previously this was only defined when $t=T$). We can then define

$$\begin{aligned} N_T(F,\theta ) = \#\{v\in \mathcal {N}_{\theta T}: Z_v^T\in F|_{[0,\theta ]}\}, \end{aligned}$$

the number of particles at time $\theta T$ whose *T*-rescaled paths have remained within *F* up to time $\theta$.

#### Proposition 2.6

Suppose that $M>1$, $\theta \in (0,1]$, $n\ge 2\,M$ and $T\ge 6\,M^{3/2}n^{3/4}$. Then for any $g\in G_M^2\cap {{\,\textrm{PL}\,}}_n^2$ and $\kappa >0$,

$$\begin{aligned} \mathbb {P}\big (N_T(\Gamma _{M,T}(g,n),\theta )\ge \kappa \big ) \le \frac{1}{\kappa }\exp \bigg (T K\Big (g,0,\frac{\lfloor \theta n\rfloor }{n}\Big ) + O\Big (\frac{M^4T}{n^{1/4}} + M^3 n T^{2/3}\Big )\bigg ). \end{aligned}$$

We will prove Proposition 2.6, which forms the heart of the argument to prove the upper bound in Theorem 1.1, in Sect. 7.2.

Our next result applies Proposition 2.6 to show that for $F\subset E^2$, at any large time *T*, the number of particles whose *T*-rescaled paths fall within *F* is unlikely to be much larger than *K*(*f*, 0, 1). Recall the definition of $M_0$ and $\delta _0$ from Lemma 2.1.

#### Proposition 2.7

Suppose that $F\subset E^2$ is closed and $M\ge 4M_0$. Then for any $\varepsilon >0$,

$$\begin{aligned} \lim _{T\rightarrow \infty } \frac{1}{T^{1/3}}\log \mathbb {P}\bigg ( N_T(F) \ge \exp \Big (T\sup _{f\in F\cap G_M^2} K(f,0,1) + T\varepsilon \Big )\bigg ) \le -\delta _0. \end{aligned}$$

The proof of this result will use Lemma 2.1 together with some technical lemmas to ensure that we can cover *F* with finitely many small balls around piecewise linear functions, and then apply Proposition 2.6. The proof is also in Sect. 7.2.

There are many paths *f* that satisfy $K^+(f)=-\infty$ but $K(f,0,1)>0$. These are paths where there exists $\theta \in (0,1)$ such that $K(f,0,\theta )<0$, and therefore the population of particles whose rescaled paths are near *f* becomes extinct around time $\theta T$. Since a population cannot recover once it becomes extinct, no particles follow such paths up to time *T* even though the expected growth by the end of the path, *K*(*f*, 0, 1), can be positive. For sets *F* that only contain such paths, Proposition 2.7 does not provide a useful bound, and we therefore need a slightly different approach.

#### Lemma 2.8

If $F\subset E^2$ is closed and $\sup _{f\in F} K^+(f) = -\infty$, then

$$\begin{aligned} \lim _{T\rightarrow \infty } \frac{1}{T^{1/3}}\log \mathbb {P}\big ( N_T(F) \ge 1\big ) \le -\delta _0. \end{aligned}$$

The proof of Lemma 2.8 is in Sect. 7.3. We can then upgrade Proposition 2.7 and Lemma 2.8, which are both statements about a particular large time *T*, to get the same result at *all* large times simultaneously.

#### Proposition 2.9

Suppose that $F\subset E^2$ is closed and $M\ge 4M_0$. Then

$$\begin{aligned} \limsup _{T\rightarrow \infty }\frac{1}{T}\log N_T(F)\le \sup _{f\in F\cap G_M^2} K(f,0,1) \end{aligned}$$

almost surely. If moreover $\sup _{f\in F} K^+(f) = -\infty$, then $\limsup _{T\rightarrow \infty } N_T(F) = 0$ almost surely.

The proof of this result will appear in Sect. 7.4. The final ingredient in our proof of the upper bound in our main theorem is a form of upper-semicontinuity for *K*.

#### Proposition 2.10

Suppose that $M\in (1,\infty )$. Then $G_M$ is compact. If $F\subset E^2$ is closed, then

$$\begin{aligned} \lim _{\varepsilon \rightarrow 0} \sup _{f\in B_d(F,\varepsilon ) \cap G_M^2} K(f,0,1) = \sup _{f\in F \cap G_M^2} K(f,0,1), \end{aligned}$$

and if $f\in E^2$ is continuous at $\theta \in (0,1]$, then

$$\begin{aligned} \lim _{\varepsilon \rightarrow 0} \sup _{g\in B_d(f,\varepsilon ) \cap G_M^2} K(g,0,\theta ) = K(f,0,\theta ). \end{aligned}$$

We will prove this in Appendix C.3. We can now complete the proof of the upper bound in our main theorem.

#### Proof of Theorem 1.1: upper bound

For each $$f\in F\cap G_M^2$$:

- If $K^+(f)=-\infty$ then take $\theta _f\in (0,1]$ such that $K(f,0,\theta _f)<0$ and *f* is continuous at $\theta _f$ (this is possible since $K(f,0,\cdot )$ has only downward jumps and *f* is continuous almost everywhere). Then take $\varepsilon _f>0$ such that $\sup _{g\in B_d(f,\varepsilon _f)\cap G_M^2} K(g,0,\theta _f)<0$, which is possible by the second part of Proposition 2.10.
- If $K^+(f)\ge 0$ then take $\varepsilon _f$ such that $\sup _{g\in B_d(f,\varepsilon _f)\cap G_M^2}K(g,0,1)\le K^+(f)+\delta$, which is possible by the first part of Proposition 2.10.

Now, since $G_M^2$ is compact (by Proposition 2.10) and *F* is closed, $F\cap G_M^2$ is compact and we may take a finite subcover of $\{B(f,\varepsilon _f/2): f\in F\cap G_M^2\}$. Say the corresponding functions are $f_1,\ldots ,f_j$, and let $B_i = \overline{B(f_i,\varepsilon _{f_i}/2)}\cap G_M^2$ for each *i*. Then for each *i*:

- If $K^+(f_i)=-\infty$ then $\sup _{g\in B_i} K^+(g) = -\infty$, so by the second part of Proposition 2.9, $\limsup _{T\rightarrow \infty } N_T(B_i) = 0$ almost surely.
- If $K^+(f_i)\ge 0$ then by the first part of Proposition 2.9,
  $$\begin{aligned} \limsup _{T\rightarrow \infty }\frac{1}{T}\log N_T(B_i) \le \sup _{g\in B_i} K(g,0,1) \le K^+(f_i)+\delta . \end{aligned}$$

Since $N_T(F)\le \sum _{i=1}^j N_T(B_i) + N_T((G_M^2)^c)$ and Proposition 2.9 implies that

$$\begin{aligned} \limsup _{T\rightarrow \infty }\frac{1}{T}\log N_T((G_M^2)^c) = -\infty , \end{aligned}$$

the result follows.

#### Sketch proof of (5)

The upper bound in expectation (5) follows more or less directly from estimates derived above. In particular, much of the proof of Proposition 2.6 involves bounding $\mathbb {E}[N_T(F)]$ from above when *F* is a small ball around a suitably nice function. From there it is a relatively simple task, similarly to the proof of Proposition 2.7, to apply Lemma 2.1 to reduce *F* to a compact set, Lemma 7.1 to cover this set with finitely many balls around suitably nice functions, and Proposition 2.10 to check that the resulting bound does not significantly overshoot (5).$\square$

## Proof outline for the lower bound in Theorem 1.1

Let $\rho$ be the metric defined by

$$\begin{aligned} \rho (f,g) = \sup _{s\in [0,1]} \Vert f(s)-g(s)\Vert = \sup _{s\in [0,1]}\big \{|f_X(s)-g_X(s)|\vee |f_Y(s)-g_Y(s)|\big \}. \end{aligned}$$

Rather than the set $\Gamma _{M,T}(f,n)$ seen in the proof of the upper bound, for the lower bound we will instead often use the set

$$\begin{aligned} \Lambda _{M,T}(f,n) = B_\rho (f,1/n^2)\cap G_{M,T}^2. \end{aligned}$$

For $F\subset E^2$ and $T>0$, recall that

$$\begin{aligned} N_T(F) = \#\{u\in \mathcal {N}_{T}: Z_u^T \in F\}, \end{aligned}$$

and for $t\in [0,1]$ and $u\in \mathcal {N}_{tT}$, define

8 $$\begin{aligned} N_{t,T}^u(F) = \#\{v\in \mathcal {N}_{T}: u\le v,\, Z_v^T|_{[t,1]} \in F|_{[t,1]}\}. \end{aligned}$$

Also let $(\mathcal {F}_t, t\ge 0)$ be the natural filtration for the process.

The main part of our proof relies on a standard second moment argument, and Propositions 3.1 and 3.2 give the first and second moment bounds necessary to carry out that argument. However, this strategy on its own cannot give strong enough estimates to be able to prove an almost sure statement, as required for Theorem 1.1. We therefore give bounds conditionally given $\mathcal {F}_{kT/n}$ for $\sqrt{n} < k \ll n$, with the aim of using the branching structure at time *kT*/*n* to increase the accuracy of our estimates.

### Proposition 3.1

Suppose that $M>1$, $n\ge 6\,M$, $\sqrt{n}\le k\le n$, $T \ge 27\,M^{3/2}n^{9/2}$ and $f\in {{\,\textrm{PL}\,}}_n^2\cap G_M^2$. Suppose also that $u\in \mathcal {N}_{kT/n}$ satisfies $\Vert Z_u^T(k/n) - f(k/n)\Vert \le \frac{1}{2n^2}$. Then

$$\begin{aligned} \mathbb {E}\big [N_{k/n,T}^u(\Lambda _{3M,T}(f,n))\,\big |\,\mathcal {F}_{kT/n}\big ] \ge \exp \Big (T K\big (f,\tfrac{k}{n},1\big ) - O\Big (\frac{M^4T}{n^{1/4}} + M^3 nT^{2/3}\Big )\Big ). \end{aligned}$$

We will prove Proposition 3.1 in Sect. 3.1.

### Proposition 3.2

Suppose that $M>1$, $n\ge 6\,M$, $\sqrt{n}\le k\le n$, $T\ge 27\,M^{3/2} n^{9/2}$ and $f\in PL_n^2\cap G_M^2$. Suppose also that $u\in \mathcal {N}_{kT/n}$ satisfies $\Vert Z_u^T(k/n) - f(k/n)\Vert < \frac{1}{n^2}$. Then

$$\begin{aligned}  &   \mathbb {E}\big [N_{k/n,T}^u(\Lambda _{3M,T}(f,n))^2\,\big |\,\mathcal {F}_{kT/n}\big ]\\  &   \le \int \limits _{k T/n}^T \! \exp \big ( \! - T K \big (f,\tfrac{k}{n},\tfrac{t}{T} \big )\big ) dt \cdot 12M^2n\exp \bigg (2T K\big (f,\tfrac{k}{n},1\big ) + O\Big (\frac{M^4 T}{n^{1/4}}+ M^3 n T^{2/3}\Big )\bigg )\\  &   + \exp \bigg (T K\big (f,\tfrac{k}{n},1\big ) + O\Big (\frac{M^4 T}{n^{1/4}}+ M^3 n T^{2/3}\Big )\bigg ). \end{aligned}$$

We will prove Proposition 3.2 in Sect. 3.2. We now use a standard second moment method to turn Propositions 3.1 and 3.2 into a lower bound on the probability that the number of particles whose rescaled paths remain near *f* is roughly *K*(*f*, *k*/*n*, 1)*T*, again conditionally on $\mathcal {F}_{kT/n}$.

### Corollary 3.3

Suppose that $M>1$, $n\ge 6\,M$, $k\ge \sqrt{n}$, $T \ge 27\,M^{3/2}n^{9/2}$ and $f\in PL_n^2\cap G_M^2$. Suppose also that $u\in \mathcal {N}_{kT/n}$ satisfies $\Vert Z_u^T(k/n) - f(k/n)\Vert \le \frac{1}{2n^2}$, and that $K(f,k/n,t)\ge 0$ for all $t\ge k/n$. Then

$$\begin{aligned}  &   \mathbb {P}\big (N_{k/n,T}^u(\Lambda _{3M,T}(f,n)) \ge e^{T K(f,k/n,1) - O(M^4T/n^{1/4} + M^3 n T^{2/3})}\,\big |\,\mathcal {F}_{kT/n}\big ) \\  &   \ge e^{-O \big (\frac{M^4 T}{n^{1/4}}+ M^3 n T^{2/3} \big )}. \end{aligned}$$

### Proof

The Paley-Zygmund inequality says that, for any non-negative random variable *X* and $\theta \in [0,1]$,

$$\begin{aligned} P\big (X\ge \theta E[X]\big ) \ge (1-\theta )^2\frac{E[X]^2}{E[X^2]}. \end{aligned}$$

Taking then *P* to be the conditional probability measure $\mathbb {P}(\,\cdot \, |\,\mathcal {F}_{kT/n})$ with $X = N_{k/n,T}^u(\Lambda _{3\,M,T}(f,n))$ and $\theta =1/2$, we have

9 $$\begin{aligned}  &   \mathbb {P}\big (N_{k/n,T}^u(\Lambda _{3M,T}(f,n)) \ge (1/2)\mathbb {E}\big [N_{k/n,T}^u(\Lambda _{3M,T}(f,n))\,\big |\,\mathcal {F}_{kT/n}\big ]\,\big |\,\mathcal {F}_{kT/n}\big )\nonumber \\  &   \ge \frac{\mathbb {E}\big [N_{k/n,T}^u(\Lambda _{3M,T}(f,n))\,\big |\,\mathcal {F}_{kT/n}\big ]^2}{4\mathbb {E}\big [N_{k/n,T}^u (\Lambda _{3M,T}(f,n))^2\,\big |\,\mathcal {F}_{kT/n}\big ]}. \end{aligned}$$

Proposition 3.1 tells us that

$$\begin{aligned} \mathbb {E}\big [N_{k/n,T}^u(\Lambda _{3M,T}(f,n))\,\big |\,\mathcal {F}_{kT/n}\big ] \ge \exp \Big (T K\big (f,\tfrac{k}{n},1\big ) - O\Big (\frac{M^4T}{n^{1/4}} + M^3 nT^{2/3}\Big )\Big ) \end{aligned}$$

and Proposition 3.2 gives

$$\begin{aligned}  &   \mathbb {E}\big [N_{k/n,T}^u(\Lambda _{3M,T}(f,n))^2\,\big |\,\mathcal {F}_{kT/n}\big ]\\  &   \le \int \limits _{k T/n}^T \! \exp \big ( \! - T K\big (f,\tfrac{k}{n},\tfrac{t}{T} \big )\big ) dt \cdot 12M^2n\exp \bigg (2T K\big (f,\tfrac{k}{n},1\big ) + O\Big (\frac{M^4 T}{n^{1/4}}+ M^3 n T^{2/3}\Big )\bigg )\\  &   + \exp \bigg (T K\big (f,\tfrac{k}{n},1\big ) + O\Big (\frac{M^4 T}{n^{1/4}}+ M^3 n T^{2/3}\Big )\bigg ). \end{aligned}$$

and since $K(f,k/n,t)\ge 0$ for all $t\ge k/n$, this reduces to

$$\begin{aligned}&\mathbb {E}\big [N_{k/n,T}^u(\Lambda _{3M,T}(f,n))^2\,\big |\,\mathcal {F}_{kT/n}\big ] \\&\hspace{20mm}\le 12M^2 n T \exp \bigg (2T K\big (f,\tfrac{k}{n},1\big ) + O\Big (\frac{M^4 T}{n^{1/4}}+ M^3 n T^{2/3}\Big )\bigg ) \\&\hspace{20mm}= \exp \bigg (2T K\big (f,\tfrac{k}{n},1\big ) + O\Big (\frac{M^4 T}{n^{1/4}}+ M^3 n T^{2/3}\Big )\bigg ). \end{aligned}$$

Substituting these estimates into (9) gives the result. $\square$

By Corollary 3.3, each particle near *f*(*kT*/*n*) at time *kT*/*n* has a not-too-small probability of having roughly $\exp (K(f,k/n,1)T)$ descendants whose rescaled paths remain near *f* up to time 1. If we can ensure that there is a reasonably large number of particles near *f*(*kT*/*n*) at time *kT*/*n*, then subject to some technicalities (for example Corollary 3.3 assumes that *f* is piecewise linear, whereas there is no such condition in Theorem 1.1) we will be able to prove the lower bound in Theorem 1.1.

The discontinuity of $R^*$ at 0 makes controlling the growth of the system at small times difficult. The first few particles in the system can have wildly different values of *R* in different realisations of the process, and it is not *a priori* clear that this cannot have a large effect on the long-term evolution of the system. Our method for showing that particles do in fact spread out in a predictable way is the following. First we show that there are many particles near the line (*s*/2, *s*/2) at time *s*, for suitable values of *s*. The idea is that our jump distribution prefers to create “almost square” rectangles (since rectangles are more likely to break along their longest side) and therefore we should see many particles near (*s*/2, *s*/2). However, since particles away from this line branch and jump more quickly, we use a discrete-time argument to keep control of the dependence between the jump locations and the jump times. A rough estimate using moments in discrete time can then be translated back into continuous time, giving the following result.

### Proposition 3.4

Define

$$\begin{aligned} V'_{n,T} = \{u\in {\mathcal {N}}_{\lceil n^{7/8}\rceil T/n}: \Vert Z_u(s) - (s/2,s/2)\Vert \le {\textstyle {\frac{T}{2n^2}}} \,\,\forall s\le \lceil n^{7/8}\rceil T/n\}. \end{aligned}$$

There exists a finite constant *C* such that for any $T\ge Cn^{48}$,

$$\begin{aligned} \mathbb {P}\big (|V'_{n,T}| < 2^{T/n^{1/8} - 2T/n^2}\big ) \le 1/T^{3/2}. \end{aligned}$$

We will prove this result in Sect. 5.1. The choice of $\lceil n^{7/8}\rceil$ is somewhat arbitrary, but ensures that there are enough particles at time $\lceil n^{7/8}\rceil T/n$ to outweigh the error arising from Corollary 3.3. The bound of $1/T^{3/2}$ is not the best possible, but is enough to use a Borel-Cantelli argument at the end of the proof of Theorem 1.1. The requirement that $T\ge Cn^{48}$ is also certainly not optimal, but since we will take $T\rightarrow \infty$, it is sufficient for our needs.

Once we have shown that there are particles near (*s*/2, *s*/2) at small times *s*, then we need to show that these particles “feed” other directions $(\lambda s',\mu s')$ for suitable $\lambda$ and $\mu$ and $s'>s$. Given $f\in G_M^2$, we will construct a function *h* that begins by moving along the line (*s*/2, *s*/2), so that we can guarantee large numbers of particles near *h* at small times using Proposition 3.4, but which then gradually changes its gradient to be closer and closer to our given function *f*. At the same time we will ensure that *h* is piecewise linear, so that we can then use Corollary 3.3 to ensure appropriate growth of particles along the whole path *h*. We then show that for $k=\lceil n^{7/8}\rceil < nt$ we have $K(h,k/n,t) \approx K(f,k/n,t)$. This is part of Proposition 3.5 below, which will be proved in Sect. 5.2.

### Proposition 3.5

Suppose that $f\in G_M^2$ satisfies $\frac{d}{dt} K(f,0,t)|_{t=0} > 0$ and $K(f,0,t)>0$ for all $t\in (0,1]$. Then for any $\varepsilon >0$ and $n\in \mathbb {N}$, there exists $h_{f,n}\in E^2$ such that

10 $$\begin{aligned} h_{f,n}(s)=(s/2,s/2) \,\,\,\,\text { for all } s \le \lceil n^{7/8}\rceil /n \end{aligned}$$

and if *n* is sufficiently large,

11 $$\begin{aligned}  &   h_{f,n}\in {{\,\textrm{PL}\,}}_n^2\cap G_M^2\cap B(f,\varepsilon ), \end{aligned}$$

12 $$\begin{aligned}  &   K(h_{f,n},\lceil n^{7/8}\rceil /n,s)>0 \,\,\,\,\text { for all } s\in (\lceil n^{7/8}\rceil /n,1] \end{aligned}$$

and

13 $$\begin{aligned} K(h_{f,n},\lceil n^{7/8}\rceil /n,1)\ge K(f,0,1)-\varepsilon . \end{aligned}$$

We will prove this in Sect. 5.2. We are now able to finish the proof of our main result.

### Proof of Theorem 1.1: lower bound

Fix $\varepsilon >0$. Recall $M_0$ from Lemma 2.1. If $\sup _{f\in F} K^-(f) = -\infty$ then there is nothing to prove. We therefore suppose that $\sup _{f\in F} K^-(f)>0$, and choose $f \in F$ with $K^-(f) > \max \{\sup _{g\in F}K^-(g)-\varepsilon ,0\}$. We can then choose $M\ge M_0$ and $\varepsilon '>0$ such that $f\in G_M^2$ and $B(f,2\varepsilon ')\subset F$.

By Proposition 3.5, for all sufficiently large $n\in \mathbb {N}$ the function $h_{f,n}$ satisfies (11), (12) and (13) with $\min \{\varepsilon /2,\varepsilon '/2\}$ in place of $\varepsilon$.

Take $n\in \mathbb {N}$ and write $k=\lceil n^{7/8}\rceil$. From Proposition 3.4, if we define

$$\begin{aligned} V'_{n,T} = \big \{u\in {\mathcal {N}}_{kT/n}: \Vert Z_u(s) - (s/2,s/2)\Vert \le {\textstyle {\frac{T}{2n^2}}} \,\,\,\forall s\le kT/n\big \}, \end{aligned}$$

then for $T\ge Cn^{48}$ and *C* large, we have $\mathbb {P}(|V'_{n,T}| \ge 2^{T/n^{1/8}-2T/n^2})\ge 1- 1/T^{3/2}$.

Since $h_{f,n}$ satisfies (11), (12) and (13) with $\min \{\varepsilon /2,\varepsilon '/2\}$ in place of $\varepsilon$,

$$\begin{aligned}&\mathbb {P}\big (N_T(B(f,\varepsilon '))<e^{(K(f,0,1)-\varepsilon )T}\big ) \\&\qquad \le \mathbb {P}\big (N_T(B(h_{f,n},\varepsilon '/2))<e^{(K(h_{f,n},k/n,1)-\varepsilon /2)T}\big )\\&\qquad \le \mathbb {E}\Big [\mathbb {P}\Big (N_T(B(h_{f,n},\varepsilon '/2))<e^{(K(h_{f,n},k/n,1)-\varepsilon /2)T}\,\Big |\,\mathcal {F}_{kT/n}\Big )\Big ]. \end{aligned}$$

Recalling the notation (8), note that if $u\in V'_{n,T}$ and $N^u_{k/n,T}(\Lambda _{3\,M,T}(h_{f,n},n)) \ge r$, and *n* is sufficiently large, then $N_T(B(h_{f,n},\varepsilon '/2))\ge r$, for any $r\ge 0$. Indeed if $u \in V'_{n,T}$ and $u \le v$ is such that $Z_v^T|_{[k/n,1]} \in \Lambda _{3\,M,T}(h_{f,n},n)|_{[k/n,1]}$ then using (10)

$$\begin{aligned}  &   \sup _{s \in [0,1]} \big \Vert Z_v^T(s)-h_{f,n}(s) \big \Vert \\  &   \quad \le \sup _{s \in [0,k/n]} \big \Vert Z_u^T(s)-\big ( \tfrac{s}{2},\tfrac{s}{2} \big ) \big \Vert + \sup _{s \in [k/n,1]} \big \Vert Z_v^T(s)-h_{f,n}(s) \big \Vert \le \frac{1}{2n^2} + \frac{1}{n^2}, \end{aligned}$$

so $Z_v^T \in B(h_{f,n}, \varepsilon '/2)$ when *n* is large. Thus

14 $$\begin{aligned}  &   \mathbb {P}\big (N_T(B(f,\varepsilon '))<e^{(K(f,0,1)-\varepsilon )T}\big )\nonumber \\  &   \quad \le \mathbb {E}\Bigg [\prod _{u\in V'_{n,T}}\mathbb {P}\Big (N^u_{k/n,T}(\Lambda _{3M,T}(h_{f,n},n))<e^{(K(h_{f,n},k/n,1)-\varepsilon /2)T}\,\Big |\,\mathcal {F}_{kT/n}\Big )\Bigg ]. \nonumber \\ \end{aligned}$$

For *n* and *T* sufficiently large, we check that we may apply Corollary 3.3: indeed, by (12), we have $K(h_{f,n},k/n,t)\ge 0$ for all $t\ge k/n$, and for $u\in V'_{n,T}$ we have

$$\begin{aligned} \Vert Z_u^T(k/n) - h_{f,n}(k/n)\Vert = \frac{1}{T}\big \Vert Z_u(kT/n) - ({\textstyle {\frac{kT}{2n}}},{\textstyle {\frac{kT}{2n}}})\big \Vert \le \frac{1}{2n^2}. \end{aligned}$$

Thus, applying Corollary 3.3 to bound the conditional probability in (14) from above, we obtain that

$$\begin{aligned} \mathbb {P}\big (N_T(B(f,\varepsilon '))<e^{(K(f,0,1)-\varepsilon )T}\big ) \le \mathbb {E}\Bigg [\prod _{u\in V'_{n,T}}\Big (1-e^{-O(M^4 T/n^{1/4} + M^3 n T^{2/3})}\Big )\Bigg ]. \end{aligned}$$

Recalling that $|V'_{n,T}|\ge 2^{T/n^{1/8}-2T/n^2}$ with probability at least $1-1/T^{3/2}$, we get

$$\begin{aligned} \mathbb {P}\big (N_T(B(f,\varepsilon '))<e^{(K(f,0,1)-\varepsilon )T}\big ) \le \big (1-e^{-O(M^4 T/n^{1/4} + M^3 n T^{2/3})}\big )^{2^{T/n^{1/8}-2T/n^2}} + 1/T^{3/2} \end{aligned}$$

and using that $1-x\le e^{-x}$ for all *x*,

15 $$\begin{aligned}  &   \mathbb {P}\big (N_T(B(f,\varepsilon '))<e^{(K(f,0,1)-\varepsilon )T}\big ) \nonumber \\  &   \le \exp \big (-2^{T/n^{1/8}-2T/n^2}e^{-O(M^4 T/n^{1/4} + M^3 n T^{2/3})}\big ) + 1/T^{3/2}. \end{aligned}$$

By Lemma 7.4 with $s=T$, for $T\ge 3M$, whenever $t-1\le T\le t$ we have

$$\begin{aligned} N_T(B(f,\varepsilon ')\cap G_{M,T}^2) \le N_t(B(f,\varepsilon '+6M/t)) \end{aligned}$$

and therefore if $T\ge 6M/\varepsilon '$, then we have

$$\begin{aligned} N_T(B(f,\varepsilon ')\cap G_{M,T}^2) \le \inf _{t\in [T,T+1]} N_t(B(f,2\varepsilon ')). \end{aligned}$$

Thus

$$\begin{aligned}&\mathbb {P}\Big (\inf _{t\in [T,T+1]} N_t(B(f,2\varepsilon '))e^{-(K(f,0,1)-\varepsilon )t}<1\Big )\\&\hspace{20mm}\le \mathbb {P}\big (N_T(B(f,\varepsilon ')\cap G_{M,T}^2)<e^{(K(f,0,1)-\varepsilon )T}\big )\\&\hspace{20mm}\le \mathbb {P}\big (N_T(B(f,\varepsilon '))<e^{(K(f,0,1)-\varepsilon )T}\big ) + \mathbb {P}\big (N_T((G_{M,T}^2)^c) \ge 1\big ). \end{aligned}$$

By Lemma 2.1, since $M\ge M_0$, the last term is at most $e^{-\delta _0 T^{1/3}}$, and then applying (15), we obtain

$$\begin{aligned}  &   \mathbb {P}\Big (\inf _{t\in [T,T+1]} N_t(B(f,2\varepsilon '))e^{-(K(f,0,1)-\varepsilon )t} < 1\Big )\\  &   \le \exp (-2^{T/n^{1/8}}e^{-O(M^4 T/n^{1/4} + M^3 n T^{2/3})}) + \frac{1}{T^{3/2}} + e^{-\delta _0 T^{1/3}}. \end{aligned}$$

Taking *n* large enough that the $2^{T/n^{1/8}}$ term dominates the exponent when *T* is large, we see that this is summable in *T*, and therefore by the Borel–Cantelli lemma,

$$\begin{aligned} \mathbb {P}\Big (\liminf _{t\rightarrow \infty } N_t(B(f,2\varepsilon ')) e^{-(K(f,0,1)-\varepsilon )t} < 1 \Big ) = 0. \end{aligned}$$

Since $B(f,2\varepsilon ')\subset F$ and $K(f,0,1) = K^-(f) > \sup _{g\in F}K^-(g) - \varepsilon$, the statement of the theorem follows.$\square$

### Sketch proof of (6)

Proving the lower bound in expectation (6) involves slightly more work than the upper bound (5). Proposition 3.5 creates a function that approximates a given *f* for much of its path, but begins by following the lead diagonal (*s*/2, *s*/2) for a short period. Unfortunately it is designed to work for functions *f* that satisfy $\frac{d}{dt} K(f,0,t)|_{t=0} > 0$ and $K(f,0,s)>0$ for all $s\in (0,1]$. To prove (6) we cannot make these assumptions on *f*, but can instead take a simpler approach than Proposition 3.5. We define a function ${\hat{h}}_{f,n}$ that follows the lead diagonal (*s*/2, *s*/2) until time $\lceil \sqrt{n}\rceil /n$, then satisfies

$$\begin{aligned} {\hat{h}}_{f,n}(j/n) = \Big (\frac{\lceil \sqrt{n}\rceil }{2n},\frac{\lceil \sqrt{n}\rceil }{2n}\Big ) + f(j/n) - f(\lceil \sqrt{n}\rceil /n) \end{aligned}$$

for every $j\in \{\lceil \sqrt{n}\rceil , \ldots , n\}$, and interpolates linearly between these values. Following a similar proof to that of Proposition 5.9, one can show that

$$\begin{aligned} \liminf _{n\rightarrow \infty } K({\hat{h}}_{f,n},0,1) \ge K(f,0,1), \end{aligned}$$

and then combining Propositions 3.1 and 3.4 yields (6).$\square$

In the proofs of the results above, it will be useful several times to note that since, for any *f*, *n*, *M* and *T*,

16 $$\begin{aligned} \Lambda _{M,T}(f,n) \subset \Gamma _{M,T}(f,n), \end{aligned}$$

we have

17 $$\begin{aligned} R_X^-(I_j,\Gamma _{M,T}(f,n),T)&\le R_X^-(I_j,\Lambda _{M,T}(f,n),T) \nonumber \\&\le R_X^+(I_j,\Lambda _{M,T}(f,n),T) \le R_X^+(I_j,\Gamma _{M,T}(f,n),T) \end{aligned}$$

and therefore by (59), if $M,T>1$, $n\ge 2M$, $f\in G_M^2$, $j\ge n^{1/2}$ and $s\in I_j$,

18 $$\begin{aligned} R_X^+(I_j,\Lambda _{M,T}(f,n),T) - \delta _{M,T}(j,n)\le &   R_X^*(f(s))\nonumber \\\le &   R_X^-(I_j,\Lambda _{M,T}(f,n),T) + \delta _{M,T}(j,n).\nonumber \\ \end{aligned}$$

### Lower bound on the first moment: proof of Proposition 3.1

Our aim in this section is to outline a proof of Proposition 3.1. Fix *f* as in the statement of the proposition. Let ${\mathcal {Z}}_0 = \{(0,0)\}$ and, for $j\in \{1,\ldots ,n-1\}$, define

$$\begin{aligned} {\mathcal {Z}}_j = \{z\in [0,\infty )^2: \Vert z-f(j/n)\Vert \le {\textstyle {\frac{1}{2n^2}}}\}. \end{aligned}$$

Lemma 2.2 combined with Lemma 2.5 will reduce the problem to bounding

$$\begin{aligned} \mathbb {Q}\Big (\xi ^T|_{[k/n,1]}\in \Lambda _{M,T}(f,n)|_{[k/n,1]}\,\Big |\, \xi ^T(k/n) = w\Big ) \end{aligned}$$

for $w\in {\mathcal {Z}}_k$, so we concentrate on estimating this quantity.

Fix $n\in \mathbb {N}$ and $M,T>1$, and consider $f\in {{\,\textrm{PL}\,}}_n^2\cap G_M^2$ and $j\in \{0,\ldots ,n-1\}$. We will use the coupling mentioned in Sect. 2.1, with details given in Sect. 6. We will apply this coupling with $I=I_j$ and $F=\Lambda _{M,T}(f,n)$. Define

$$\begin{aligned}  &   q^X_{n,M,T}(z,j,f)\\  &   \quad = Q_z^{I_j,\Lambda _{M,T}(f,n),T} \Big ( \big | X_-(s)- f_X(s)\big | \le {\textstyle {\frac{1}{n^2}}} \,\,\forall s\in I_j, \\  &   \qquad \qquad \qquad \qquad \qquad \qquad \big |X_-\big ({\textstyle {\frac{j+1}{n}}}\big )-f_X\big ({\textstyle {\frac{j+1}{n}}}\big )\big | \le {\textstyle {\frac{1}{2n^2}}}, \ X_-|_{I_j}\in G_{M,T}|_{I_j} \Big ) \end{aligned}$$

and

$$\begin{aligned} {\hat{q}}^X_{n,M,T}(z,j,f) = Q_z^{I_j,\Lambda _{M,T}(f,n),T}\Big ( X_+({\textstyle {\frac{j+1}{n}}})-X_-({\textstyle {\frac{j+1}{n}}}) =0\Big ) \end{aligned}$$

and similarly for $q^Y_{n,M,T}(z,j,f)$ and ${\hat{q}}^Y_{n,M,T}(z,j,f)$.

#### Lemma 3.6

Suppose that $n\ge 3$, $f\in {{\,\textrm{PL}\,}}_n^2$ and $T>1$. Then for any $k\in \{0,\ldots ,n-1\}$ and $w\in {\mathcal {Z}}_k$,

$$\begin{aligned}  &   \mathbb {Q}\Big (\xi ^T|_{[k/n,1]}\in \Lambda _{M,T}(f,n)|_{[k/n,1]}\,\Big |\, \xi ^T(k/n) = w\Big )\\  &   \ge \prod _{j=k}^{n-1} \inf _{z\in {\mathcal {Z}}_j} q^X_{n,M,T}(z,j,f)\, {\hat{q}}^X_{n,M,T}(z,j,f)\, q^Y_{n,M,T}(z,j,f)\, \hat{q}^Y_{n,M,T}(z,j,f). \end{aligned}$$

We carry out the proof of Lemma 3.6, which consists of applying the properties of the coupling defined in Sect. 6.2. We then need to bound the terms on the right-hand side. Bounding the ${\hat{q}}$ terms is fairly straightforward.

#### Lemma 3.7

Suppose that $M>1$, $n\ge 2M$, $T>1$ and $f\in {{\,\textrm{PL}\,}}_n^2 \cap G_M^2$. Then for any $k\ge \lceil n^{1/2}\rceil$,

$$\begin{aligned} \prod _{j=k}^{n-1} \inf _{z\in {\mathcal {Z}}_j} {\hat{q}}^X_{n,M,T}(z,j,f) {\hat{q}}^Y_{n,M,T}(z,j,f) \ge \exp \Big (-O\Big (\frac{M^4T}{n^{1/2}} + M^3 n\Big )\Big ). \end{aligned}$$

Again we will prove Lemma 3.7 in Sect. 6.2. Bounding the *q* terms is much more delicate. In the following lemma, the precise form of $\Delta (j)$ is not important; we consider it a small term.

#### Lemma 3.8

Suppose that $M>1$, $n\ge 2M$, $T>8n^{9/2}M^{3/2}$ and $f\in {{\,\textrm{PL}\,}}_n^2 \cap G_M^2$. Then for any $j\in \{\lceil \sqrt{n}\rceil ,\ldots ,n-1\}$ and $z=(x,y)\in {\mathcal {Z}}_j$,

$$\begin{aligned} q^X_{n,3M,T}(z,j,f) \ge \exp \Big (-T\int \limits _{j/n}^{(j+1)/n} \Big (\sqrt{2R^*_X(f(s))}-\sqrt{f'_X(s)}\Big )^2 ds - T\Delta (j)\Big ) \end{aligned}$$

where

$$\begin{aligned} \Delta (j) = \frac{2(M+1)}{n^{3/2}} + \frac{2\delta _{M,T}(j,n)}{n} + \frac{1}{\sqrt{n}}\sqrt{2\delta _{M,T}(j,n)\big (f_X({\textstyle {\frac{j+1}{n}}})-f_X({\textstyle {\frac{j}{n}}})\big )} \end{aligned}$$

and $\delta _{M,T}(j,n)$ is defined in Lemma A.1.

We again delay the proof of Lemma 3.8 to Sect. 6.2. Putting the above ingredients together and bounding $\sum _{j=\lceil \sqrt{n}\rceil /n}^{n-1}\Delta (j)$ gives us our main bound, which we now state.

#### Proposition 3.9

Suppose that $M>1$, $n\ge 2M$, $T>8n^{9/2}M^{3/2}$ and $f\in {{\,\textrm{PL}\,}}_n^2\cap G_M^2$. Then for any $k\ge \lceil \sqrt{n}\rceil$ and $w\in {\mathcal {Z}}_{k}$,

$$\begin{aligned}  &   \mathbb {Q}\Big (\xi ^T|_{[k/n,1]}\in \Lambda _{3M,T}(f,n)|_{[k/n,1]}\,\Big |\, \xi ^T(k/n) = w\Big )\\  &   \ge \exp \bigg ( -TI(f,k/n,1) - O\Big (\frac{M^4T}{n^{1/4}} + M^3 n T^{1/2}\Big )\bigg ). \end{aligned}$$

#### Proof

Combining Lemmas 3.6, 3.7 and 3.8, we have

$$\begin{aligned}  &   \mathbb {Q}\Big (\xi ^T|_{[k/n,1]}\in \Lambda _{3M,T}(f,n)|_{[k/n,1]}\,\Big |\, \xi ^T(k/n) = w\Big )\\  &   \ge \exp \bigg ( -TI(f,k/n,1) - 2T\sum _{j=k}^{n-1}\Delta (j) - O\Big (\frac{M^4T}{n^{1/2}} + M^3 n\Big )\bigg ). \end{aligned}$$

Recall that

$$\begin{aligned} \Delta (j) = \frac{2(M+1)}{n^{3/2}} + \frac{2\delta _{M,T}(j,n)}{n} + \sqrt{\frac{2\delta _{M,T}(j,n)}{n}\big (f_X({\textstyle {\frac{j+1}{n}}})-f_X({\textstyle {\frac{j}{n}}})\big )}. \end{aligned}$$

By (60),

$$\begin{aligned} \sum _{j=\lceil \sqrt{n}\rceil }^{n-1}\frac{\delta _{M,T}(j,n)}{n} = O\Big (\frac{M^4}{n^{1/2}}+\frac{M^3 n}{T}\Big ). \end{aligned}$$

By Cauchy–Schwarz,

$$\begin{aligned}  &   \sum _{j=\lceil \sqrt{n}\rceil }^{n-1} \sqrt{\frac{2\delta _{M,T}(j,n)}{n}\big (f_X({\textstyle {\frac{j+1}{n}}})-f_X({\textstyle {\frac{j}{n}}})\big )}\\  &   \le \bigg (\sum _{j=\lceil \sqrt{n}\rceil }^{n-1} \frac{2\delta _{M,T}(j,n)}{n}\sum _{i=\lceil \sqrt{n}\rceil }^{n-1} \big (f_X({\textstyle {\frac{i+1}{n}}})-f_X({\textstyle {\frac{i}{n}}})\big )\bigg )^{1/2}. \end{aligned}$$

Using (60) again, together with the fact that $f\in G_M^2$, and that $\sqrt{a+b}\le \sqrt{a}+\sqrt{b}$ for $a,b\ge 0$, we have

$$\begin{aligned} \sum _{j=\lceil \sqrt{n}\rceil }^{n-1} \frac{1}{\sqrt{n}}\sqrt{2\delta _{M,T}(j,n)\big (f_X({\textstyle {\frac{j+1}{n}}})-f_X({\textstyle {\frac{j}{n}}})\big )}&= O\Big (\frac{M^2}{n^{1/4}} + \frac{M^{3/2}n^{1/2}}{T^{1/2}}\Big )M^{1/2} \\&= O\Big (\frac{M^{5/2}}{n^{1/4}} + \frac{M^2n^{1/2}}{T^{1/2}}\Big ). \end{aligned}$$

Therefore

$$\begin{aligned} \sum _{j=k}^{n-1} \Delta (j) \le \sum _{j=\lceil \sqrt{n}\rceil }^{n-1} \Delta (j) = O\Big (\frac{M}{n^{1/2}} + \frac{M^4}{n^{1/2}}+\frac{M^3 n}{T} + \frac{M^{5/2}}{n^{1/4}} + \frac{M^2n^{1/2}}{T^{1/2}}\Big ). \end{aligned}$$

Combining error terms gives the result. $\square$

As promised, we can now easily prove Proposition 3.1.

#### Proof of Proposition 3.1

For $u \in \mathcal {N}_{kT/n}$, let $\mathcal {N}_T^{(u)}$ be the set of descendants of *u* in $\mathcal {N}_T$. Since $u \in \mathcal {N}_{kT/n}$, by the Markov property and Lemma 2.2, for any $k\in \{0,\ldots ,n-1\}$,

$$\begin{aligned}  &   \mathbb {E}\Bigg [\sum _{v\in \mathcal {N}_T^{(u)}} \mathbbm {1}_{\{Z_v^T|_{[k/n,1]}\in \Lambda _{3M,T}(f,n)|_{[k/n,1]}\}}\,\Bigg |\,\mathcal {F}_{kT/n}\Bigg ]\\  &   = \mathbb {Q}\Big [\mathbbm {1}_{\{\xi ^T|_{[k/n,1]}\in \Lambda _{3M,T}(f,n)|_{[k/n,1]}\}}e^{\int \limits _{kT/n}^T R(\xi _s)ds} \,\Big |\, \xi ^T(k/n)=w \Big ]\Big |_{w=Z_u^T(k/n)}. \end{aligned}$$

Now, since $k\ge \lceil \sqrt{n}\rceil$ and $f\in G^2_M\subset G^2_{3\,M}$, by (16) and Lemma 2.5, if $\xi ^T|_{[k/n,1]}\in \Lambda _{3M,T}(f,n)|_{[k/n,1]}$, then

$$\begin{aligned} \int \limits _{kT/n}^T R(\xi _s)ds = T\int \limits _{k/n}^1 R(T\xi ^T(s)) ds \ge T\int \limits _{k/n}^1 R^*(f(s))ds - T\eta (3M,n,T), \end{aligned}$$

and therefore

$$\begin{aligned}  &   \mathbb {E}\Bigg [\sum _{v\in \mathcal {N}_T^{(u)}} \mathbbm {1}_{\{Z_v^T|_{[k/n,1]}\in \Lambda _{3M,T}(f,n)|_{[k/n,1]}\}}\,\Bigg |\,\mathcal {F}_{kT/n}\Bigg ]\\  &   \ge e^{T\int \limits _{k/n}^1 R^*(f(s))ds - T\eta (3M,n,T)}\\  &   \cdot \mathbb {Q}\Big (\xi ^T|_{[k/n,1]}\in \Lambda _{3M,T}(f,n)|_{[k/n,1]} \,\Big |\, \xi ^T(k/n)=w \Big )\Big |_{w=Z_u^T(k/n)}. \end{aligned}$$

We also know from Proposition 3.9 that if $w\in \mathcal Z_{k}$, then

$$\begin{aligned}  &   \mathbb {Q}\Big (\xi ^T|_{[k/n,1]}\in \Lambda _{3M,T}(f,n)|_{[k/n,1]}\,\Big |\, \xi ^T(k/n) = w\Big )\\  &   \quad \ge \exp \bigg ( -TI(f,k/n,1) - O\Big (\frac{M^4T}{n^{1/4}} + M^3 nT^{1/2}\Big )\bigg ). \end{aligned}$$

Combining these estimates and recalling that $\eta (3M,n,T)=O(M^4n^{-1/2} + M^3nT^{-1/3})$ gives the result.

### Upper bound on the second moment: proof of Proposition 3.2

For our first moment bounds we used the many-to-one lemma, Lemma 2.2, which gives a method for calculating expectations of sums over all the particles in our population at a fixed time. For our second moment bound, we will need an analogue for calculating expectations of squares of sums over particles. This will involve another measure $\mathbb {Q}^2$, whose description is again adapted from [27], this time in the case $k=2$.

Let $\mathbb {Q}^2$ be a probability measure under which $\xi ^1_t$ and $\xi ^2_t$ are Markov processes each living in $\mathbb {R}^2$ constructed in the following way:

- Take an exponential random variable $\mathbbm {e}$ of parameter 1.
- Let $(\chi _t,t\ge 0)$ be a pure jump Markov process in $\mathbb {R}^2$ independent of $\mathbbm {e}$ such that $\chi _0=0$ and when $\chi _t$ is in state *z*, jumps occur at rate 2*R*(*z*). When there is a jump from state *z*, it is of the form $(\mathcal {E},0)$ with probability *P*(*z*) and $(0,\mathcal {E})$ with probability $1-P(z)$, where $\mathcal {E}$ is an independent exponentially-distributed random variable with parameter 1.
- Let $$\tau = \inf \{t>0: \int _0^t 2R(\chi _s) ds > \mathbbm {e}\}$$.
- Let $$\xi ^1_t = \xi ^2_t = \chi _t$$ for $$t<\tau $$.
- Let $\xi ^1_\tau$ equal $\chi _\tau$ plus a jump of the form $(-\log \mathcal {U},0)$ with probability $P(\chi _\tau )$ and $(0,-\log \mathcal {U})$ with probability $1-P(\chi _\tau )$, where $\mathcal {U}$ is an independent uniformly distributed random variable on (0, 1); let $\xi ^2_\tau$ equal $\chi _\tau$ plus either $(-\log (1-\mathcal {U}),0)$ or $(0,-\log (1-\mathcal {U}))$ respectively.
- Conditionally on $\tau$, $(\xi _t^1)_{t\le \tau }$ and $(\xi _t^2)_{t\le \tau }$, the processes $(\xi _{\tau +t}^1, t\ge 0)$ and $(\xi _{\tau +t}^2, t\ge 0)$ behave independently as if under $\mathbb {Q}_{\xi _\tau ^1}$ and $\mathbb {Q}_{\xi _\tau ^2}$ respectively.

We write $\mathbb {Q}^2$ both for the measure and for its corresponding expectation operator.

#### Lemma 3.10

(Many-to-two, Lemma 1 of [27] with $k=2$) Suppose that $t\ge 0$. For any measurable function $f:(\mathbb {R}^2)^2\rightarrow \mathbb {R}$,

$$\begin{aligned} \mathbb {E}\Bigg [ \sum _{u_1, u_2 \in \mathcal {N}_t} \! f(Z_{u_1}(t), Z_{u_2}(t)) \Bigg ] \! =\! \mathbb {Q}^2 \! \left[ f(\xi _t^1, \xi _t^2) e^{3 \int \limits _0^{\tau \wedge t} R(\xi ^1_s) ds +\int \limits _{\tau \wedge t}^t R(\xi ^1_s) ds + \int \limits _{\tau \wedge t}^t R(\xi ^2_s) ds} \right] \!. \end{aligned}$$

In fact, by using the description of $\mathbb {Q}^2$ above, the key to the second moment bound will be to estimate terms of the form

$$\begin{aligned} \mathbb {Q}\big (\xi ^T|_{[a,b]}\in \Lambda _{M,T}(f,n)\big |_{[a,b]}\,\big |\,\xi ^T_a=z\big ) \end{aligned}$$

where $\mathbb {Q}=\mathbb {Q}_0$ is the measure seen in Sect. 2.1. The same coupling used for Proposition 2.3 will yield the following result.

#### Proposition 3.11

Suppose that $f\in E^2$, $n\in \mathbb {N}$, $T>1$ and $M>1$. Then for any $0\le a<b\le 1$ and *z* such that $\Vert z-f(a)\Vert <1/n^2$,

$$\begin{aligned}  &   \mathbb {Q}\big (\xi ^T|_{[a,b]}\in \Lambda _{M,T}(f,n)\big |_{[a,b]}\,\big |\,\xi ^T_a=z\big )\\  &   \le \exp \bigg (-T\sum _{j=\lfloor an\rfloor }^{\lceil bn\rceil -1} \big ({\mathcal {E}}^+_X(I_j\cap [a,b],\Lambda _{M,T}(f,n),T) + \mathcal E^+_Y(I_j\cap [a,b],\Lambda _{M,T}(f,n),T)\big )\bigg ). \end{aligned}$$

We postpone the details of the proof to Sect. 6. We then need to relate the right-hand side in Proposition 3.11 to our rate function, in the form of the following lemma.

#### Lemma 3.12

Suppose that $M,T>1$, $n\ge 2\,M$ and $f\in PL_n^2 \cap G_M^2$. Then for any *a*, *b* such that $\lceil \sqrt{n}\rceil /n\le a<b\le 1$,

$$\begin{aligned}  &   \sum _{j=\lfloor an\rfloor }^{\lceil bn\rceil -1} \Big (\mathcal E_X^+(I_j\cap [a,b],\Lambda _{M,T}(f,n),T)+\mathcal E_Y^+(I_j\cap [a,b],\Lambda _{M,T}(f,n),T)\Big ) \\  &   \ge I(f,a,b) - O\Big (\frac{M^4}{n^{1/4}}+\frac{M^3 n}{T^{1/2}}\Big ). \end{aligned}$$

The proof of Lemma 3.12 is similar to the deterministic bounds required for the upper bound in Sect. 2.2, but also uses the uniform structure of $\Lambda _{M,T}(f,n)$ and therefore requires slightly different estimates. We carry this out in Appendix A.3, and for now continue to the proof of Proposition 3.2. The proof is fairly long, but uses only the ingredients above together with bounds already developed for the upper bound on the first moment.

#### Proof of Proposition 3.2

Recall the construction of $\mathbb {Q}^2$ together with the Markov processes $\xi ^1$ and $\xi ^2$ above. For $s\ge 0$ and $T>0$, write $\xi ^{1,T}_s = \xi ^1_{sT}/T$ and $\xi ^{2,T}_s = \xi ^2_{sT}/T$. For $i=1,2$, define the event

$$\begin{aligned} {\mathcal {B}}_i = \{\xi ^{i,T}|_{[k/n,1]} \in \Lambda _{3M,T}(f,n)|_{[k/n,1]}\} \end{aligned}$$

and for the single spine $\xi$ defined under $\mathbb {Q}$, define

$$\begin{aligned} {\mathcal {B}}(a,b) = \{\xi ^T|_{[a,b]} \in \Lambda _{3M,T}(f,n)|_{[a,b]}\}. \end{aligned}$$

By Lemma 3.10,

$$\begin{aligned}&\mathbb {E}\Bigg [\Bigg (\sum _{v\in \mathcal {N}_T^{(u)}} \mathbbm {1}_{\{Z_v^T|_{[k/n,1]} \in \Lambda _{3M,T}(f,n)|_{[k/n,1]}\}}\Bigg )^2\,\Bigg |\,\mathcal {F}_{kT/n}\Bigg ]\\&= \mathbb {Q}^2 \Big [\mathbbm {1}_{{\mathcal {B}}_1\cap {\mathcal {B}}_2} e^{3\int \limits _{kT/n}^{T\wedge \tau } R(\xi ^1_s)ds + \int \limits _{T\wedge \tau }^T R(\xi ^1_s)ds + \int \limits _{T\wedge \tau }^T R(\xi ^2_s)ds} \Big | \tau >{\textstyle {\frac{kT}{n}}},\, \xi ^{1,T}_{k/n} \!= z\Big ]\Big |_{z=Z_u^T(k/n)}. \end{aligned}$$

From the construction of $\mathbb {Q}^2$ before Lemma 3.10, it is clear that $\tau$ has a density, and that

$$\begin{aligned}&\mathbb {Q}^2\Big [\mathbbm {1}_{{\mathcal {B}}_1\cap {\mathcal {B}}_2} e^{3\int \limits _{kT/n}^{T\wedge \tau } R(\xi ^1_s)ds + \int \limits _{T\wedge \tau }^T R(\xi ^1_s)ds + \int \limits _{T\wedge \tau }^T R(\xi ^2_s)ds} \,\Big |\, \tau>{\textstyle {\frac{kT}{n}}},\, \xi ^{1,T}_{k/n} = z\Big ]\\&\quad \le \int \limits _{kT/n}^T \Big ( \mathbb {Q}^2\Big [\mathbbm {1}_{{\mathcal {B}}_1\cap \mathcal B_2\cap \{\tau \in dt\}} \,\Big |\, \tau>{\textstyle {\frac{kT}{n}}},\, \xi ^{1,T}_{k/n} = z\Big ] \\&\quad \cdot \sup _{g\in \Lambda _{3M,T}(f,n)} e^{3\int \limits _{kT/n}^t R(Tg(s/T))ds + 2\int \limits _t^T R(Tg(s/T))ds} \Big ) \\&\quad + \mathbb {Q}^2\Big [\mathbbm {1}_{{\mathcal {B}}_1\cap \mathcal B_2\cap \{\tau>T\}} \,\Big |\, \tau >{\textstyle {\frac{kT}{n}}},\, \xi ^{1,T}_{k/n} = z\Big ] \sup _{g\in \Lambda _{3M,T}(f,n)} e^{3\int \limits _{kT/n}^T R(Tg(s/T))ds}. \end{aligned}$$

It also follows from the construction of $\mathbb {Q}^2$ before Lemma 3.10 that

$$\begin{aligned}&\mathbb {Q}^2\Big [\mathbbm {1}_{{\mathcal {B}}_1\cap {\mathcal {B}}_2\cap \{\tau \in dt\}} \,\Big |\, \tau >{\textstyle {\frac{kT}{n}}},\, \xi ^{1,T}_{k/n} = z\Big ]\\&\quad \le \mathbb {Q}\Big [\mathbbm {1}_{{\mathcal {B}}(\frac{k}{n},\frac{t}{T})} 2R(\xi _t)e^{-2\int \limits _{{kT/n}}^t R(\xi _s)ds}dt\,\Big |\,\xi ^T_{\frac{k}{n}} = z\Big ] \sup _{\Vert w-f(\frac{t}{T})\Vert<\frac{1}{n^2}} \mathbb {Q}\Big (\mathcal B\big (\tfrac{t}{T},1\big )\,\Big |\,\xi ^T_{\frac{t}{T}}=w\Big )^2\\&\quad \le \mathbb {Q}\Big (\mathcal {B}\big (\tfrac{k}{n},\tfrac{t}{T}\big ) \,\Big |\,\xi ^T_{k/n} = z\Big )\sup _{\Vert w-f(t/T)\Vert <1/n^2} \mathbb {Q}\Big ({\mathcal {B}}\big (\tfrac{t}{T},1\big ) \,\Big |\,\xi ^T_{t/T}=w\Big )^2\\&\quad \cdot \sup _{h\in \Lambda _{3M,T}(f,n)} 2R(Th(t/T))e^{-2T\int \limits _{k/n}^{t/T} R(Th(s))ds} \end{aligned}$$

and that

$$\begin{aligned}&\mathbb {Q}^2\Big [\mathbbm {1}_{{\mathcal {B}}_1\cap {\mathcal {B}}_2\cap \{\tau>T\}} \,\Big |\, \tau >{\textstyle {\frac{kT}{n}}},\, \xi ^{1,T}_{k/n} = z\Big ]\\&\quad = \mathbb {Q}\Big [\mathbbm {1}_{{\mathcal {B}}(k/n,1)}e^{-2\int \limits _{kT/n}^T R(\xi _s)ds}\,\Big |\,\xi ^T_{k/n} = z\Big ]\\&\quad \le \mathbb {Q}\Big ({\mathcal {B}}(k/n,1)\,\Big |\,\xi ^T_{k/n} = z\Big ) \sup _{h\in \Lambda _{3M,T}(f,n)} e^{-2T\int \limits _{k/n}^1 R(Th(s))ds}. \end{aligned}$$

Combining these bounds, we have shown that

19 $$\begin{aligned}&\mathbb {E}\Bigg [\Bigg (\sum _{v\in \mathcal {N}_T^{(u)}} \mathbbm {1}_{\{Z_v^T|_{[k/n,1]} \in \Lambda _{3M,T}(f,n)|_{[k/n,1]}\}}\Bigg )^2\,\Bigg |\,\mathcal {F}_{kT/n}\Bigg ]\nonumber \\&\quad \le \int \limits _{\frac{kT}{n}}^T \mathbb {Q}\Big (\mathcal B\big (\tfrac{k}{n},\tfrac{t}{T}\big )\,\Big |\,\xi ^T_{k/n} = z\Big )\Big |_{z=Z_u^T(\frac{k}{n})} \cdot \sup _{\Vert w-f(t/T)\Vert <1/n^2} \mathbb {Q}\Big (\mathcal B\big (\tfrac{t}{T},1\big )\,\Big |\,\xi ^T_{t/T}=w\Big )^2 \nonumber \\&\quad \cdot \sup _{h\in \Lambda _{3M,T}(f,n)} 2R(Th(t/T))e^{-2T\int \limits _{k/n}^{t/T} R(Th(s))ds} \nonumber \\&\quad \cdot \sup _{g\in \Lambda _{3M,T}(f,n)} e^{3T\int \limits _{k/n}^{t/T} R(Tg(s))ds + 2T\int \limits _{t/T}^1 R(Tg(s))ds}dt\nonumber \\&\quad + \mathbb {Q}\Big ({\mathcal {B}}(k/n,1)\,\Big |\,\xi ^T_{k/n} = z\Big )\Big |_{z=Z_u^T(k/n)} \cdot \sup _{h\in \Lambda _{3M,T}(f,n)} e^{-2T\int \limits _{k/n}^1 R(Th(s))ds} \nonumber \\&\quad \cdot \sup _{g\in \Lambda _{3M,T}(f,n)} e^{3T\int \limits _{k/n}^1 R(Tg(s))ds}. \end{aligned}$$

Recall that $k\ge \lceil \sqrt{n}\rceil$. By (16) and Lemma 2.5, for any $t\in [kT/n,T]$,

$$\begin{aligned} \sup _{g\in \Lambda _{3M,T}(f,n)}\int \limits _{k/n}^{t/T} R(Tg(s))ds \le \int \limits _{k/n}^{\lfloor nt/T\rfloor /n} R^*(f(s))ds + \eta (3M,n,T) \end{aligned}$$

and

$$\begin{aligned} \inf _{h\in \Lambda _{3M,T}(f,n)}\int \limits _{k/n}^{t/T} R(Th(s))ds \ge \int \limits _{k/n}^{\lfloor nt/T\rfloor /n} R^*(f(s))ds - \eta (3M,n,T). \end{aligned}$$

Thus

$$\begin{aligned}  &   \sup _{h\in \Lambda _{3M,T}(f,n)} e^{-2T\int \limits _{k/n}^{t/T} R(Th(s))ds} \cdot \sup _{g\in \Lambda _{3M,T}(f,n)} e^{3T\int \limits _{k/n}^{t/T} R(Tg(s))ds + 2T\int \limits _{t/T}^1 R(Tg(s))ds}\\  &   \le \exp \bigg ( -T\int \limits _{k/n}^{\lfloor nt/T\rfloor /n} R^*(f(s))ds + 2T\int \limits _{ k/n}^1 R^*(f(s))ds + 7T\eta (3M,n,T)\bigg ). \end{aligned}$$

Similarly,

20 $$\begin{aligned}  &   \sup _{h\in \Lambda _{3M,T}(f,n)} e^{-2T\int \limits _{k/n}^1 R(Th(s))ds} \cdot \sup _{g\in \Lambda _{3M,T}(f,n)} e^{3T\int \limits _{k/n}^1 R(Tg(s))ds}\nonumber \\  &   \le \exp \bigg (T\int \limits _{k/n}^1 R^*(f(s))ds + 5T\eta (3M,n,T)\bigg ). \end{aligned}$$

By the definition of $G_{M,T}$, plus the assumption that $T^{2/3}\ge 9Mn^{1/2}$, for any $t\in [kT/n,T]$ we also have

$$\begin{aligned} \sup _{h\in \Lambda _{3M,T}(f,n)} 2R(Th(t/T)) \le 2\frac{TM(t/T+2T^{-2/3})+1}{T(t/(MT)-2T^{-2/3})} \le \frac{6MT}{Tk/(2Mn)} \le 12M^2n. \end{aligned}$$

The above estimates bound the non-probabilistic terms in (19). For the other terms we apply Proposition 3.11 and Lemma 3.12 to obtain the bound

$$\begin{aligned}&\mathbb {Q}\Big ({\mathcal {B}}(a,b) \,\Big |\,\xi ^T_{a}=z\Big )\\&\le \exp \bigg (\! -T\sum _{j=\lfloor an\rfloor }^{\lceil bn\rceil -1} \big ({\mathcal {E}}^+_X(I_j\cap [a,b],\Lambda _{3M,T}(f,n),T) + \mathcal E^+_Y(I_j\cap [a,b],\Lambda _{3M,T}(f,n),T)\big )\bigg )\\&\le \exp \Big (\! -TI(f,a,b) + O\Big (\frac{M^4 T}{n^{1/4}}+ M^3 n T^{1/2}\Big )\Big ). \end{aligned}$$

Putting all these ingredients together, we obtain that

21 $$\begin{aligned}&\mathbb {E}\Bigg [\Bigg (\sum _{v\in \mathcal {N}_T^{(u)}} \mathbbm {1}_{\{Z_v^T|_{[k/n,1]} \in \Lambda _{3M,T}(f,n)|_{[k/n,1]}\}}\Bigg )^2\,\Bigg |\,\mathcal {F}_{kT/n}\Bigg ]\nonumber \\&\quad \le \int \limits _{kT/n}^T \exp \bigg (\!\! -TI(f,k/n,t/T)-2TI(f,t/T,1)+ O\Big (\frac{M^4 T}{n^{1/4}}+ M^3 n T^{1/2}\Big )\bigg ) \nonumber \\&\quad \cdot 12M^2n \exp \bigg ( \! \! -T\! \int \limits _{k/n}^{\lfloor nt/T\rfloor /n} \! R^*(f(s))ds + 2T\! \int \limits _{k/n}^1 \! R^*(f(s))ds + 7T\eta (3M,n,T)\bigg ) dt\nonumber \\&\quad + \exp \bigg (\! \! -TI(f,k/n,1)+ O\Big (\frac{M^4 T}{n^{1/4}}+ M^3 n T^{1/2}\Big ) \bigg ) \nonumber \\&\quad \cdot \exp \bigg ( T\! \int \limits _{k/n}^1 R^*(f(s))ds + 5T\eta (3M,n,T)\bigg ). \end{aligned}$$

Using that $f\in {{\,\textrm{PL}\,}}_n^2$ and therefore is absolutely continuous, we see that

$$\begin{aligned}  &   -I(f,k/n,t/T) - 2I(f,t/T,1) - \int \limits _{k/n}^{\lfloor nt/T\rfloor /n} R^*(f(s))ds + 2\int \limits _{k/n}^1 R^*(f(s))ds\\  &   \le 2 K(f,k/n,1) - K(f,k/n,t/T) + O(M^2/n). \end{aligned}$$

The result follows from substituting this into (21) and recalling from Lemma 2.5 that

$$\begin{aligned} \eta (3M,n,T) = O\Big (\frac{M^4}{n^{1/2}} + \frac{M^3n}{T^{1/3}}\Big ). \end{aligned}$$

$$\square $$ $$\square $$

## Detailed construction and ruling out difficult paths: proof of Lemma 2.1

In this section, we prove Lemma 2.1, which said that for large *M* all particles are (*M*, *T*)-good with high probability as $T\rightarrow \infty$. We will begin by defining a discrete tree with labels to represent the positions and split times of particles, which besides being a necessary step in our proof, also provides a formal construction of the process introduced in Sect. 1.

Take an infinite binary tree ${\mathbb {T}}$ and let ${\mathbb {T}}_n$ be the vertices in the *n*th generation of ${\mathbb {T}}$, so that $|{\mathbb {T}}_n|=2^n$. Attach to each vertex $v\in {\mathbb {T}}$ two independent random variables ${\mathcal {U}}^{\text {split}}_v$ and ${\mathcal {U}}^{\text {dir}}_v$, both uniformly distributed on (0, 1). Also attach another independent random variable $\mathbbm {e}_v$ which is exponentially distributed with parameter 1.

We recursively define random variables $B_v$, $H_v$ and $T_v$ for each vertex $v\in {\mathbb {T}}$, which represent the base, height and birth time of the rectangle corresponding to *v*. Write $\rho$ for the unique vertex in ${\mathbb {T}}_0$, which we call the root. Under the probability measure $\mathbb {P}_{a,b}$, set $B_\rho = a$, $H_\rho = b$ and $T_\rho =0$. We write $\mathbb {P}$ as shorthand for $\mathbb {P}_{1,1}$.

Now take an integer $n\ge 0$ and suppose that we have defined $B_u$, $H_u$ and $T_u$ for all vertices *u* in generations $0,\ldots ,n$. For a vertex $v\in \mathbb {T}_n$, define

$$\begin{aligned} D_v = {\left\{ \begin{array}{ll} 1 &  \text { if } {\mathcal {U}}^{\text {dir}}_v\le P(-\log B_v, -\log H_v)\\ 0 &  \text { if } {\mathcal {U}}^{\text {dir}}_v > P(-\log B_v, -\log H_v).\end{array}\right. } \end{aligned}$$

Write *v*1 and *v*2 for the two children of *v* in generation $n+1$. If $D_v=1$, then set

$$\begin{aligned} B_{v1} = {\mathcal {U}}^{\text {split}}_v B_v, \,\,\,\, B_{v2} = (1-{\mathcal {U}}^{\text {split}}_v) B_v, \,\,\,\,\text { and } \,\,\,\, H_{v1}=H_{v2}=H_v; \end{aligned}$$

if on the other hand $D_v=0$, then set

$$\begin{aligned} H_{v1} = {\mathcal {U}}^{\text {split}}_v H_v, \,\,\,\, H_{v2} = (1-{\mathcal {U}}^{\text {split}}_v) H_v, \,\,\,\,\text { and } \,\,\,\, B_{v1}=B_{v2}=B_v. \end{aligned}$$

Then, for each $v\in {\mathbb {T}}$, define

$$\begin{aligned} X_v = -\log B_v \,\,\,\,\text { and }\,\,\,\, Y_v = -\log H_v. \end{aligned}$$

Finally, set

$$\begin{aligned} T_{v1} = T_{v2} = T_v + \frac{\mathbbm {e}_{v}}{R(X_v,Y_v)}. \end{aligned}$$

We now translate this discrete-time process (with continuous labels) into the continuous-time model described in the introduction. For each $t\ge 0$, define

$$\begin{aligned} \mathcal {N}_t = \Big \{v\in {\mathbb {T}}: T_v\le t<T_v+\frac{\mathbbm {e}_{v}}{R(X_v,Y_v)}\Big \}, \end{aligned}$$

the set of particles that are alive at time *t*. Then for $v\in \mathcal {N}_t$ and $s\le t$, if *u* is the unique ancestor of *v* in ${\mathbb {T}}$ that satsfies $T_u\le s<T_u+\mathbbm {e}_{u}/R(X_u,Y_u)$, then set $B_v(s) = B_u$, $H_v(s)=H_u$, $X_v(s) = X_u$ and $Y_v(s) = Y_u$. We call $Z_v(s) = (X_v(s),Y_v(s))$ the *position* of particle *v* at time *s*. For $T>0$, we can also consider particles’ paths rescaled by *T*, by which we mean, for $s\le t$ and $v\in \mathcal {N}_{tT}$,

$$\begin{aligned} X_v^T(s) = \frac{X_v(sT)}{T}, \,\,\,\, Y_v^T(s) = \frac{Y_v(sT)}{T}, \,\,\,\, Z_v^T(s) = (X_v^T(s),Y_v^T(s)). \end{aligned}$$

If we have $v\in \mathcal {N}_T$ then we may refer to $X_v^T$ to mean the function $X_v^T:[0,1]\rightarrow \mathbb {R}$, and similarly for $Y_v^T$ and $Z_v^T$.

### Lemma 4.1

For any $\kappa >0$, there exists $M>1$ and $N\in \mathbb {N}$ such that

$$\begin{aligned} \mathbb {P}\Big (\exists v\in {\mathbb {T}}_n : X_v \not \in [n/M, Mn] \,&\text { or }\, Y_v \not \in [n/M, Mn] \,\\  &\text { or }\, T_v < n/M \,\text { or }\, T_v+\frac{\mathbbm {e}_{v}}{R(X_v,Y_v)} > Mn\Big ) \le e^{-\kappa n} \end{aligned}$$

for all $$n\ge N$$.

### Proof

Note that for any $u\in {\mathbb {T}}_n$, $X_u$ is the sum of *n* random variables, each of which is (stochastically) bounded above by an independent exponential random variable with parameter 1 (this is the distribution of $-\log U$ when *U* is *U*(0, 1)). Thus, if $E\sim \text {Exp}(1)$,

$$\begin{aligned} \mathbb {P}(X_u > Mn) \le \mathbb {E}[e^{X_u/2}]e^{-Mn/2} \le \mathbb {E}[e^{E/2}]^n e^{-Mn/2} = 2^n e^{-Mn/2} \end{aligned}$$

and, since there are $2^n$ vertices in ${\mathbb {T}}_n$, a union bound gives

$$\begin{aligned} \mathbb {P}(\exists v\in {\mathbb {T}}_n: X_v > Mn) \le 4^n e^{-Mn/2}. \end{aligned}$$

By choosing *M* large enough, we can make this smaller than $e^{-\kappa n}$. By symmetry the same holds for $Y_v$.

For a lower bound on $X_v$ and $Y_v$, we first give a lower bound on $X_v+Y_v$. Indeed, note that for $u\in {\mathbb {T}}_n$, $X_u+Y_u$ is a sum of *n* independent random variables, each of which is exponentially distributed with parameter 1. Thus, for any $\lambda >0$ and any $u\in \mathbb {T}_n$,

$$\begin{aligned} \mathbb {P}(X_u+Y_u < n/M) \le \mathbb {E}[e^{-\lambda (X_u+Y_u)}]e^{\lambda n/M} = \mathbb {E}[e^{-\lambda E}]^n e^{\lambda n/M} = \frac{1}{(1+\lambda )^n} e^{\lambda n/M}, \end{aligned}$$

so that we can choose $M_0$ large enough that

22 $$\begin{aligned} \mathbb {P}(X_u+Y_u < n/M_0) \le 2^{-2n-2}e^{-2\kappa (n+1)}. \end{aligned}$$

Take $u\in \mathbb {T}_n$, let $u'$ be the unique ancestor of *u* in ${\mathbb {T}}_{\lfloor n/2\rfloor }$ and take $M>M_0$. Note that, applying (22), if $n \ge 6$

23 $$\begin{aligned}&\mathbb {P}(\exists v\in {\mathbb {T}}_n : X_v\wedge Y_v< n/M - 1)\nonumber \\&\le \mathbb {E}[\#\{v\in {\mathbb {T}}_n : X_v\wedge Y_v< n/M-1\}] = 2^n\mathbb {P}(X_u\wedge Y_u< n/M-1)\nonumber \\&\le 2^n\mathbb {P}(X_u\wedge Y_u< n/M-1 \text { and } X_{u'}+Y_{u'}\ge \lfloor \tfrac{n}{2} \rfloor /M_0) + 2^n\mathbb {P}(X_{u'}+Y_{u'}< \lfloor \tfrac{n}{2} \rfloor /M_0)\nonumber \\&\le 2^n\mathbb {P}(X_u\wedge Y_u< n/M-1 \text { and } X_{u'}+Y_{u'}\ge n/(3M_0)) + 2^n\cdot 2^{-2\lfloor \tfrac{n}{2} \rfloor -2}e^{-2\kappa \big (\lfloor \tfrac{n}{2} \rfloor +1\big )}\nonumber \\&\le 2^n\mathbb {P}(X_u\wedge Y_u < n/M-1 \text { and } X_{u'}+Y_{u'}\ge n/(3M_0)) + e^{-\kappa n}/2. \end{aligned}$$

Now, if $X_u\wedge Y_u < n/M-1$ and $X_{u'}+Y_{u'}\ge n/(3M_0)$, then for all vertices *v* on the path from $u'$ to *u*, we have

$$\begin{aligned} \frac{X_v\vee Y_v + 1}{X_v\wedge Y_v+1} \ge \frac{X_v+Y_v - X_v \wedge Y_v + 1}{X_v\wedge Y_v+1} \ge \frac{X_v+Y_v}{X_v\wedge Y_v+1} -1 \ge \frac{M}{3M_0}-1. \end{aligned}$$

Recalling the definition of *P*, this means that

$$\begin{aligned} P(X_v, Y_v) \ge 1- \frac{X_v \wedge Y_v+1}{2(X_v \vee Y_v +1)} \ge 1- \frac{1}{2M/(3M_0)-2} \end{aligned}$$

and the same holds for $1-P(X_v,Y_v)$. This means that $X_u\wedge Y_u - X_{u'}\wedge Y_{u'}$ consists of $\lceil n/2\rceil$ random variables, each of which is (stochastically) bounded below by an independent random variable $E'$ which is zero with probability $1/(2M/(3M_0)-2)$ and equals an independent copy of *E* with probability $1-1/(2M/(3M_0)-2)$. Thus, for any $\lambda >0$,

$$\begin{aligned}&\mathbb {P}(X_u\wedge Y_u < n/M-1 \text { and } X_{u'}+Y_{u'}\ge n/M_0)\\&\hspace{25mm}\le \mathbb {E}\Big [e^{-\lambda X_u\wedge Y_u} \mathbbm {1}_{\big \{\frac{X_v\vee Y_v + 1}{X_v\wedge Y_v+1} \ge \frac{M}{3M_0}-1 \big \}}\Big ]e^{\lambda n/M}\\&\hspace{25mm}\le \mathbb {E}\Big [e^{-\lambda (X_u\wedge Y_u - X_{u'}\wedge Y_{u'} )} \mathbbm {1}_{\big \{\frac{X_v\vee Y_v + 1}{X_v\wedge Y_v+1} \ge \frac{M}{3M_0}-1\big \}}\Big ]e^{\lambda n/M}\\&\hspace{25mm}\le \mathbb {E}[e^{-\lambda E'}]^{\lceil n/2 \rceil }e^{\lambda n/M}\\&\hspace{25mm}\le \Big (\frac{1}{2M/(3M_0)-2} + \mathbb {E}[e^{-\lambda E}]\Big (1-\frac{1}{2M/(3M_0)-2}\Big )\Big )^{\lceil n/2 \rceil }e^{\lambda n/M}\\&\hspace{25mm}\le \Big (\frac{1}{2M/(3M_0)-2} + \frac{1}{\lambda +1}\Big )^{\lceil n/2 \rceil }e^{\lambda n/M}. \end{aligned}$$

By choosing $\lambda$ large and then *M* large, we can ensure that this is smaller than $2^{-n} e^{-\kappa n}/2$, which when combined with (23), shows that for *n* sufficiently large,

24 $$\begin{aligned} \mathbb {P}\big (\exists v\in {\mathbb {T}}_n: X_v \not \in [n/M, Mn] \,\text { or }\, Y_v \not \in [n/M, Mn]\big )\le e^{-\kappa n}. \end{aligned}$$

We now turn to $T_v$. As for $X_v$ and $Y_v$, the upper bound is easy: since $R(x,y)\ge 1$ for all *x* and *y*, for any $u\in \mathbb {T}_n$ we have

$$\begin{aligned}  &   \mathbb {P}\Big (\exists v\in \mathbb {T}_n: T_v + \frac{\mathbbm {e}_v}{R(X_v,Y_v)}> Mn \Big ) \le 2^n \mathbb {P}\bigg (\sum _{w\le u} \frac{\mathbbm {e}_w}{R(X_w,Y_w)}> Mn\bigg )\\  &   \le 2^n \mathbb {P}\bigg (\sum _{w\le u} \mathbbm {e}_w > Mn\bigg ) \le 2^n \mathbb {E}[e^{E/2}]^{n+1} e^{-Mn/2} = 2^{2n+1}e^{-Mn/2}, \end{aligned}$$

which can be made smaller than $e^{-\kappa n}$ by choosing *M* large.

For a lower bound on $T_v$, define the event

$$\begin{aligned} \Upsilon _{n,M} = \{X_v \in [k/M, Mk] \,\text { and }\, Y_v \in [k/M, Mk] \,\,\,\,\forall v\in \mathbb {T}_k,\,\,\forall k\ge n\}. \end{aligned}$$

By (24), for any $\kappa >0$, we may choose *N* and $M_0$ sufficiently large that

25 $$\begin{aligned} \mathbb {P}(\Upsilon _{n,M_0}^c) \le \sum _{j=n}^\infty \mathbb {P}\big (\exists v\in {\mathbb {T}}_n: X_v \not \in [ \tfrac{n}{M_0}, M_0n ] \,\text { or }\, Y_v \not \in [ \tfrac{n}{M_0}, M_0n ] \big ) \le 2^{-2n-3}e^{-2\kappa (n+1)}\nonumber \\ \end{aligned}$$

for all $n\ge N$. Fix $u\in \mathbb {T}_n$ and let $\rho = u_0,u_1,u_2,\ldots ,u_n = u$ be the unique path from the root $\rho$ to *u* in the tree. Then for $n\ge 2N$,

26 $$\begin{aligned} \mathbb {P}(T_u< n/M)&= \mathbb {P}\bigg (\sum _{j=0}^{n-1} \frac{\mathbbm {e}_{u_j}}{R(X_{u_j},Y_{u_j})}< \frac{n}{M}\bigg )\nonumber \\&\le \mathbb {P}(\Upsilon _{\lfloor n/2\rfloor ,M_0}^c) + \mathbb {P}\bigg (\Upsilon _{\lfloor n/2\rfloor ,M_0}\cap \bigg \{\sum _{j=\lfloor n/2\rfloor }^n \frac{\mathbbm {e}_{u_j}}{R(X_{u_j},Y_{u_j})} < \frac{n}{M}\bigg \}\bigg ). \end{aligned}$$

Since $n\ge 2N$, we have

27 $$\begin{aligned} \mathbb {P}(\Upsilon _{\lfloor n/2\rfloor ,M_0}^c) \le 2^{-2\lfloor n/2\rfloor -3} e^{-2\kappa (\lfloor n/2\rfloor +1)}/2 \le 2^{-n-1}e^{-\kappa n}. \end{aligned}$$

On the event $\Upsilon _{\lfloor n/2\rfloor ,M_0}$, we have

$$\begin{aligned} R(X_{u_j},Y_{u_j})\le \frac{M_0 j + 1}{j/M_0 +1} \le M_0^2 \end{aligned}$$

for all $j\ge \lfloor n/2\rfloor$; therefore

$$\begin{aligned} \mathbb {P}\bigg (\Upsilon _{\lfloor n/2\rfloor ,M_0}\cap \bigg \{\sum _{j=\lfloor n/2\rfloor }^n \frac{\mathbbm {e}_{u_j}}{R(X_{u_j},Y_{u_j})}< \frac{n}{M}\bigg \}\bigg ) \le \mathbb {P}\bigg (\sum _{j=\lfloor n/2\rfloor }^n \frac{\mathbbm {e}_{u_j}}{M_0^2} < \frac{n}{M}\bigg ). \end{aligned}$$

But for any $\lambda >0$,

$$\begin{aligned}  &   \mathbb {P}\bigg (\sum _{j=\lfloor n/2\rfloor }^n \frac{\mathbbm {e}_{u_j}}{M_0^2} < \frac{n}{M}\bigg ) = \mathbb {P}\big (e^{-\lambda \sum _{j=\lfloor n/2\rfloor }^n \mathbbm {e}_{u_j}} > e^{-\lambda M_0^2 n/M}\big )\\  &   \le \mathbb {E}[e^{-\lambda \sum _{j=\lfloor n/2\rfloor }^n \mathbbm {e}_{u_j}}]e^{\lambda M_0^2 n/M} \le \mathbb {E}[e^{-\lambda E}]^{n/2} e^{\lambda M_0^2 n/M} = \frac{1}{(1+\lambda )^{n/2}} e^{\lambda M_0^2 n/M}. \end{aligned}$$

Substituting this and (27) into (26), and taking a union bound, we have

$$\begin{aligned} \mathbb {P}(\exists v\in {\mathbb {T}}_n: T_v< n/M) \le 2^n\mathbb {P}(T_u < n/M) \le e^{-\kappa n}/2 + \frac{2^n}{(1+\lambda )^{n/2}} e^{\lambda M_0^2 n/M}, \end{aligned}$$

which can be made smaller than $e^{-\kappa n}$ by choosing $\lambda$ large and then *M* large. $\square$

Fix $\alpha \in (0,1)$ and define the event

$$\begin{aligned} {\mathcal {G}}_M(T)= &   \Big \{X_v\in \Big [\frac{n}{M}-T^\alpha , Mn+T^\alpha \Big ],\, Y_v\in \Big [\frac{n}{M}-T^\alpha , Mn+T^\alpha \Big ],\\  &   T_v \ge \frac{n}{M}-T^\alpha \,\text { and }\, T_v+\mathbbm {e}_v \le Mn +T^\alpha \,\,\,\,\forall v\in {\mathbb {T}}_n \,\,\,\,\forall n\ge 0\Big \}. \end{aligned}$$

### Corollary 4.2

There exist $M>1$ and $\delta >0$ such that for any $T\ge 0$,

$$\begin{aligned} \mathbb {P}({\mathcal {G}}_M(T)^c) \le \exp (-\delta T^\alpha ). \end{aligned}$$

### Proof

By Lemma 4.1 we may choose $M\in (1,\infty )$ such that for all *n* large enough,

$$\begin{aligned} \mathbb {P}\big (\exists v\in {\mathbb {T}}_n: X_v \not \in [\tfrac{n}{M}, Mn] \,\text { or }\, Y_v \not \in [\tfrac{n}{M}, Mn] \,\text { or }\, T_v {<} \tfrac{n}{M} \,\text { or }\, T_v+\mathbbm {e}_v {>} Mn \big ) {\le } e^{-n}. \end{aligned}$$

Let

$$\begin{aligned} {\mathcal {G}}_{M,n}(T)= &   \Big \{X_v\in \Big [\frac{n}{M}-T^\alpha , Mn+T^\alpha \Big ],\, Y_v\in \Big [\frac{n}{M}-T^\alpha , Mn+T^\alpha \Big ],\\  &   T_v \ge \frac{n}{M}-T^\alpha \,\text { and }\, T_v+\mathbbm {e}_v \le Mn +T^\alpha \,\,\,\,\forall v\in {\mathbb {T}}_n\Big \}, \end{aligned}$$

so that ${\mathcal {G}}_M(T) = \bigcap _{n=0}^\infty \mathcal G_{M,n}(T)$. For $n \le T^\alpha /M$, since $n/M-T^\alpha \le 0$, we have

$$\begin{aligned} \bigcap _{n=0}^{\lfloor T^\alpha /M\rfloor } {\mathcal {G}}_{M,n}(T) \supset \big \{X_v\le T^\alpha ,\, Y_v\le T^\alpha \,\text { and }\, T_v+\mathbbm {e}_v \le T^\alpha \,\,\,\,\forall v\in {\mathbb {T}}_{\lfloor T^\alpha /M\rfloor }\big \} \end{aligned}$$

and thus, by our choice of *M*,

$$\begin{aligned}  &   \mathbb {P}\bigg (\bigcup _{n=0}^{\lfloor T^\alpha /M \rfloor } \mathcal G_{M,n}(T)^c\bigg ) \\  &   \le \mathbb {P}\big (\exists v\in {\mathbb {T}}_{\lfloor T^\alpha /M\rfloor }: X_v> T^\alpha ,\,\text { or }\, Y_v> T^\alpha \,\text { or }\, T_v+\mathbbm {e}_v > T^\alpha \big ) \le e^{-\lfloor T^\alpha /M\rfloor }. \end{aligned}$$

On the other hand, for $n>T^\alpha /M$,

$$\begin{aligned} {\mathcal {G}}_{M,n}(T) \supset \Big \{X_v \! \in \! \Big [\frac{n}{M}, Mn\Big ],\, Y_v \! \in \! \Big [\frac{n}{M}, Mn\Big ],\, T_v \ge \frac{n}{M}\text { and } T_v+\mathbbm {e}_v \le Mn\,\,\forall v\in {\mathbb {T}}_n\Big \} \end{aligned}$$

so by our choice of *M*, $\mathbb {P}({\mathcal {G}}_{M,n}(T)^c) \le e^{-n}$. Summing this over all $n>T^\alpha /M$ and combining with the bound for $n\le T^\alpha /M$, then choosing $\delta < 1/M$, completes the proof. $\square$

We can now prove our main result for this section, Lemma 2.1.

### Proof of Lemma 2.1

For $t\ge 0$, suppose that $u\in {\mathcal {N}}_{t}$ and let *n*(*u*) be the unique *n* such that $u\in {\mathbb {T}}_n$. By the definition of $\mathcal {N}_{t}$, we have $T_u\le t<T_u+\mathbbm {e}_u$. On $\mathcal G_M(T)$, we have $T_v+\mathbbm {e}_v \le t$ for all $v\in {\mathbb {T}}_n$ with $Mn+T^\alpha \le t$; so we must have $n(u)>(t-T^\alpha )/M$. Similarly, on $\mathcal {G}_M(T)$, we have $T_v>t$ for all $v\in \mathbb T_n$ with $n/M-T^\alpha >t$; so we must have $n(u)\le M(t+T^\alpha )$. Thus, on $\mathcal {G}_M(T)$, we have

$$\begin{aligned} \frac{t-T^\alpha }{M} < n(u)\le M(t+T^\alpha ) \end{aligned}$$

and therefore also

$$\begin{aligned} \frac{t-T^\alpha }{M^2}-T^\alpha< X_u \le M^2 (t+T^\alpha ) + T^\alpha \,\,\,\text { and }\,\,\, \frac{t-T^\alpha }{M^2}-T^\alpha < Y_u \le M^2 (t+T^\alpha ) + T^\alpha . \end{aligned}$$

Since this holds for any particle $u\in \mathcal {N}_t$ for any $t\ge 0$, taking $\alpha =1/3$ and rescaling by *T* we deduce that on $\mathcal G_M(T)$, the paths of all particles fall within $G_{M^2,T}^2$, and the result follows from Corollary 4.2.

## Growth of the population at small times

In this section we prove two results that are essentially concerned with showing that the number of particles near any reasonable straight line $(\lambda s,\mu s)$, $s\ge 0$, grows exponentially fast. The first of these results is Proposition 3.4, which considers the case $\lambda =\mu =1/2$; the idea in this case is that our rectangles prefer to be “roughly square”, and relatively simple moment bounds will show that there are indeed many particles near this line. This will be the content of Sect. 5.1. We then move on to proving Proposition 3.5, which concerns a function that begins by moving along the line (*s*/2, *s*/2) but then gradually shifts its gradient towards a general slope $(\lambda s,\mu s)$. This is done in Sect. 5.2.

### The lead diagonal: proof of Proposition 3.4

Recall the discrete-time setup from Sect. 4. In order to initially remove the dependence between time and space, let ${\tilde{T}}_\rho = 0$, and recursively for each $v\in \mathbb {T}$ let ${\tilde{T}}_{v1} = {\tilde{T}}_{v2} = {\tilde{T}}_v + \mathbbm {e}_v$.

For $v\in {\mathbb {T}}_k$ and $j\le k$, write $X_v(j)$ to mean $X_u$ where *u* is the unique ancestor of *v* in ${\mathbb {T}}_j$. Similarly write $Y_v(j)$, $T_v(j)$, ${\tilde{T}}_v(j)$ and $Z_v(j)$. Also define

$$\begin{aligned} \Delta _v(j) = X_v(j)-Y_v(j) \;\;\text { and }\;\; S_v(j) = X_v(j) + Y_v(j) - j, \end{aligned}$$

and let $(\mathcal {G}_j, j\ge 0)$ be the natural filtration of the discrete-time process. We begin with sixth moment estimates on $\Delta _v(j)$ and $S_v(j)$. The reason for using the sixth moment is that this eventually gives us a decay of order 1/*T*, which will be strong enough for our purposes. We could use higher moments if we wanted to get a better rate of decay.

#### Lemma 5.1

There exists a finite constant *C* such that for any vertex $v\in {\mathbb {T}}_k$ and $0\le j\le k$, we have

$$\begin{aligned}\mathbb {E}\big [(X_v(j) - Y_v(j))^6\big ] \le Cj^3\end{aligned}$$

and

$$\begin{aligned}\mathbb {E}[(X_v(j) + Y_v(j) - j)^6] \le Cj^3.\end{aligned}$$

#### Proof

Let $v_j$ be the vertex in ${\mathbb {T}}_j$ consisting of all 1s, i.e. $v_j = v_{j-1}1$ for all *j*. By symmetry it suffices to consider $v=v_k$. Letting $E_j = -\log \mathcal U_{v_j}^{\text {split}}$, we see by construction that

$$\begin{aligned} X_{v_j} - X_{v_{j-1}} = D_{v_{j-1}}E_{j-1}\;\;\text { and }\;\;Y_{v_j} - Y_{v_{j-1}} = (1-D_{v_{j-1}})E_{j-1}. \end{aligned}$$

We also note that $\{E_j: j\ge 0\}$ is a collection of independent exponentially distributed random variables of parameter 1, such that $E_j$ is independent of $D_{v_j}$ for each *j*.

Let $\Delta _j = X_{v_j} - Y_{v_j}$. We begin by bounding the second moment of $\Delta _j$, then the fourth moment, before we tackle the sixth moment. By the above,

28 $$\begin{aligned} \mathbb {E}\big [\Delta _j^2\big |\mathcal {G}_{j-1}\big ]&= \mathbb {E}\big [\big (\Delta _{j-1} + (2D_{v_{j-1}}-1)E_{j-1}\big )^2\big |\mathcal {G}_{j-1}\big ]\nonumber \\&= \Delta _{j-1}^2 + 2\Delta _{j-1}\mathbb {E}[(2D_{v_{j-1}}\!-1)E_{j-1}|\mathcal {G}_{j-1}]+ \mathbb {E}[(2D_{v_{j-1}}\!-1)^2E_{j-1}^2|\mathcal {G}_{j-1}]\nonumber \\&= \Delta _{j-1}^2 + 2\Delta _{j-1}\mathbb {E}[2D_{v_{j-1}}\!-1|\mathcal {G}_{j-1}]+ 2, \end{aligned}$$

where the last line follows from the independence of $E_{j-1}$ from $D_{v_{j-1}}$ and $\mathcal {G}_{j-1}$ and the fact that $(2D_{v_{j-1}}-1)^2 = 1$. Now we note that, from the definition of $D_{v_j}$, if $\Delta _j\ge 0$ then $D_{v_j}$ equals 1 with probability at most 1/2, whereas if $\Delta _j \le 0$ then $D_{v_j}$ equals 1 with probability at least 1/2. Thus

29 $$\begin{aligned} \Delta _{j-1}\mathbb {E}[2D_{v_{j-1}}-1|\mathcal {G}_{j-1}] \le 0, \end{aligned}$$

so that (28) becomes

$$\begin{aligned}\mathbb {E}\big [\Delta _j^2\big |\mathcal {G}_{j-1}\big ] \le \Delta _{j-1}^2 + 2.\end{aligned}$$

Taking expectations and summing over $i\le j$, we obtain

30 $$\begin{aligned} \mathbb {E}\big [\Delta _j^2\big ] \le 2j. \end{aligned}$$

We now move on to the fourth moment, following a very similar argument:

31 $$\begin{aligned}&\mathbb {E}\big [\Delta _j^4\big |\mathcal {G}_{j-1}\big ] = \mathbb {E}\big [\big (\Delta _{j-1} + (2D_{v_{j-1}}-1)E_{j-1}\big )^4\big |\mathcal {G}_{j-1}\big ]\nonumber \\&\quad = \Delta _{j-1}^4 + 4\Delta _{j-1}^3\mathbb {E}[(2D_{v_{j-1}}-1)E_{j-1}|\mathcal {G}_{j-1}]+ 6\Delta _{j-1}^2\mathbb {E}[(2D_{v_{j-1}}-1)^2E_{j-1}^2|\mathcal {G}_{j-1}]\nonumber \\&\quad + 4\Delta _{j-1}\mathbb {E}[(2D_{v_{j-1}}-1)^3E_{j-1}^3|\mathcal {G}_{j-1}] + \mathbb {E}[(2D_{v_{j-1}}-1)^4E_{j-1}^4|\mathcal {G}_{j-1}] \nonumber \\&\quad = \Delta _{j-1}^4 + 4\Delta _{j-1}^3\mathbb {E}[2D_{v_{j-1}}-1|\mathcal {G}_{j-1}]+ 6\Delta _{j-1}^2\mathbb {E}[E_{j-1}^2]\nonumber \\&\quad + 4\Delta _{j-1}\mathbb {E}[2D_{v_{j-1}}-1|\mathcal {G}_{j-1}]\mathbb {E}[E_{j-1}^3] + \mathbb {E}[E_{j-1}^4], \end{aligned}$$

where again for the last line we used the independence of $E_{j-1}$ from $D_{v_{j-1}}$ and $\mathcal {G}_{j-1}$ and the fact that $(2D_{v_{j-1}}-1)^2 = 1$. By (29) and the facts that $\mathbb {E}[E_{j-1}^2]=2$ and $\mathbb {E}[E_{j-1}^4]=24$, we obtain

$$\begin{aligned}\mathbb {E}\big [\Delta _j^4\big |\mathcal {G}_{j-1}\big ] \le \Delta _{j-1}^4 + 12\Delta _{j-1}^2 + 24.\end{aligned}$$

Taking expectations and using (30), we have

$$\begin{aligned}\mathbb {E}\big [\Delta _j^4\big ] \le \mathbb {E}[\Delta _{j-1}^4] + 24(j-1) + 24 = \mathbb {E}[\Delta _{j-1}^4] + 24j.\end{aligned}$$

Summing over $i\le j$, this gives

32 $$\begin{aligned} \mathbb {E}\big [\Delta _j^4\big ] \le \sum _{i=1}^j 24j = 12j(j+1). \end{aligned}$$

For the sixth moment, the same strategy, expanding out $\Delta _j^6 = (\Delta _{j-1} + (2D_{v_{j-1}}-1)E_{j-1})^6$ and using the independence of $E_{j-1}$ from $D_{v_{j-1}}$ and $\mathcal {G}_{j-1}$, and then applying (29), works again. Omitting the calculations, the upshot is that

$$\begin{aligned}\mathbb {E}\big [\Delta _j^6\big |\mathcal {G}_{j-1}\big ] \le \Delta _{j-1}^6 + 30\Delta _{j-1}^4 + 360\Delta _{j-1}^2 + 720.\end{aligned}$$

Taking expectations and using (30) and (32), we have

$$\begin{aligned}\mathbb {E}\big [\Delta _j^6\big ] \le \mathbb {E}[\Delta _{j-1}^6] + 360j(j+1) + 720j + 720 = \mathbb {E}[\Delta _{j-1}^6] + 360(j+1)(j+2).\end{aligned}$$

Summing over $i\le j$, we have

$$\begin{aligned}\mathbb {E}\big [\Delta _j^6\big ] \le 360\sum _{i=1}^j (i+1)(i+2) = 120j(j^2+6j+11),\end{aligned}$$

which proves the first part of the lemma.

The second statement of the lemma is much simpler to prove, since $X_{v_j} + Y_{v_j} = \sum _{i=0}^{j-1}E_i$. Either by direct calculation or by using the moment generating function, one may write down an expression for every moment of $X_v(j) + Y_v(j) - j$; in particular one may check that

$$\begin{aligned} \mathbb {E}[(X_v(j) + Y_v(j) - j)^6] \le Cj^3 \end{aligned}$$

for some constant *C*, completing the proof. $\square$

#### Lemma 5.2

Let $L(n,T) = \lceil n^{7/8}\rceil T/n + \lceil 2T/n^2\rceil$ and let

$$\begin{aligned} U_{n,T} = \big \{v\in \mathbb {T}_{L(n,T)}:\Vert Z_v(k) - (\tfrac{k}{2}, \tfrac{k}{2}) \Vert \le {\textstyle {\frac{T}{32n^4}}} \text { and } |\tilde{T}_v(k)-k|\le {\textstyle {\frac{T}{4n^2}}} \,\,\forall k\le L(n,T)\big \}. \end{aligned}$$

Then there exists a finite constant *C* such that for any $T\ge Cn^{48}$,

$$\begin{aligned}\mathbb {P}\big (|U_{n,T}|\ge {\textstyle {\frac{1}{2T^2}}} 2^{L(n,T)}\big ) \ge 1-T^{-3/2}.\end{aligned}$$

#### Proof

Note that, for any $L\ge 0$, $v\in \mathbb {T}_{L}$ and $k\le L$, by the triangle inequality we have

$$\begin{aligned} |X_v(k)-k/2|&= \frac{1}{2}|X_v(k)+Y_v(k)-k + X_v(k)-Y_v(k)| \\&\le \frac{1}{2}|X_v(k)+Y_v(k)-k| + \frac{1}{2}|X_v(k)-Y_v(k)|, \end{aligned}$$

and similarly for $|Y_v(k)-k/2|$. Thus

$$\begin{aligned}  &   \mathbb {P}\big (\Vert Z_v(k) - (\tfrac{k}{2}, \tfrac{k}{2})\Vert> {\textstyle {\frac{T}{32n^4}}}\big ) \\  &   \le \mathbb {P}\big (|X_v(k) +Y_v(k) - k|> {\textstyle {\frac{T}{32n^4}}}\big ) + \mathbb {P}\big (|X_v(k) -Y_v(k)| > {\textstyle {\frac{T}{32n^4}}}\big ). \end{aligned}$$

Applying Markov’s inequality and the sixth moment estimates from Lemma 5.1, we obtain

$$\begin{aligned}  &   \mathbb {P}\big (\Vert Z_v(k) - (\tfrac{k}{2}, \tfrac{k}{2})\Vert > {\textstyle {\frac{T}{32n^4}}}\big ) \\  &   \le \mathbb {E}\big [|X_v(k) +Y_v(k) - k|^6\big ]\big ({\textstyle {\frac{32 n^{4}}{T}}}\big )^6 + \mathbb {E}\big [|X_v(k) -Y_v(k)|^6\big ]\big ({\textstyle {\frac{32 n^{4}}{T}}}\big )^6 \le 2Ck^3\big ({\textstyle {\frac{32 n^{4}}{T}}}\big )^6 \end{aligned}$$

where *C* is a finite constant.

Now note that ${\tilde{T}}_v(k)$ is a sum of *k* independent exponential random variables of parameter 1, and therefore has the same distribution as $X_v(k)+Y_v(k)$. Thus, again by Lemma 5.1,

$$\begin{aligned}\mathbb {E}\big [|{\tilde{T}}_v(k) - k|^6\big ] \le Ck^3\end{aligned}$$

and therefore

$$\begin{aligned} \mathbb {P}\big (|{\tilde{T}}_v(k)-k| > {\textstyle {\frac{T}{4n^2}}}\big ) \le \mathbb {E}\big [|\tilde{T}_v(k) - k|^6\big ]\big ({\textstyle {\frac{4 n^{2}}{T}}}\big )^6 \le Ck^3\big ({\textstyle {\frac{4 n^{2}}{T}}}\big )^6. \end{aligned}$$

We deduce that, for some finite constant $C'$,

33 $$\begin{aligned} \mathbb {P}\big (\exists k\le L: \Vert Z_v(k) - (\tfrac{k}{2}, \tfrac{k}{2}) \Vert> {\textstyle {\frac{T}{32n^4}}} \text { or } |{\tilde{T}}_v(k)-k| > {\textstyle {\frac{T}{4n^2}}}\big ) \le \sum _{k=1}^{L} \frac{C' k^3 n^{24}}{T^6} \le \frac{C'L^4 n^{24}}{T^6}.\nonumber \\ \end{aligned}$$

Since $L(n,T) = O(n^{-1/8} T) \le O(T)$, this is at most $\frac{C'' n^{24}}{T^2}$ for some finite constant $C''$.

$$\begin{aligned} \textrm{Thus}\,\, \mathbb {P}\big (\exists k\le L(n,T): \Vert Z_v(k)-(\tfrac{k}{2}, \tfrac{k}{2})\Vert> {\textstyle {\frac{T}{32n^4}}} \text { or } |{\tilde{T}}_v(k)-k| > {\textstyle {\frac{T}{4n^2}}}\big ) \le \frac{C'' n^{24}}{T^2} \end{aligned}$$

for some finite constant $C''$.

Converting the above to a statement about $U_{n,T}$, since there are $2^{L(n,T)}$ vertices in $\mathbb {T}_{L(n,T)}$, we have shown that

$$\begin{aligned} \mathbb {E}\big [|U_{n,T}|\big ] = 2^{L(n,T)}\mathbb {P}(v\in U_{n,T}) \ge 2^{L(n,T)}\Big (1 - \frac{C'' n^{24}}{T^2}\Big ). \end{aligned}$$

Obviously we also have

$$\begin{aligned} \mathbb {E}\big [|U_{n,T}|^2\big ] \le 2^{2L(n,T)}, \end{aligned}$$

and therefore by the Paley-Zygmund inequality,

$$\begin{aligned} \mathbb {P}\bigg (|U_{n,T}|\ge \frac{1}{T^2} 2^{L(n,T)}\Big (1 - \frac{C'' n^{24}}{T^2}\Big ) \bigg )&\ge \bigg (1-\frac{1}{T^2}\bigg )^2\frac{\mathbb {E}\big [|U_{n,T}|\big ]^2}{\mathbb {E}\big [|U_{n,T}|^2\big ]}\\&\ge \bigg (1-\frac{2}{T^2}\bigg )\Big (1 - \frac{C'' n^{24}}{T^2}\Big )^2. \end{aligned}$$

The result follows. $\square$

#### Lemma 5.3

Define

$$\begin{aligned} V_{n,T} = \big \{v\in \mathbb {T}_{L(n,T)}: \Vert Z_v(k) - (\tfrac{k}{2}, \tfrac{k}{2})\Vert \le {\textstyle {\frac{T}{32n^4}}} \text { and } |T_v(k)-k|\le {\textstyle {\frac{7T}{8n^2}}} \,\,\,\forall k\le L(n,T)\big \}, \end{aligned}$$

where $L(n,T) = \lceil n^{7/8}\rceil T/n + \lceil 2T/n^2\rceil$ as in Lemma 5.2. Then there exists a finite constant *C* such that for any $T\ge Cn^{48}$,

$$\begin{aligned}\mathbb {P}\big (|V_{n,T}|\ge {\textstyle {\frac{1}{2T^2}}} 2^{L(n,T)}\big ) \ge 1-T^{-3/2}.\end{aligned}$$

#### Proof

We claim that every $v\in U_{n,T}$ is also in $V_{n,T}$. By Lemma 5.2 this is sufficient to complete the proof.

Take $v\in U_{n,T}$. In particular, for each $k\le L(n,T)$, we have $\Vert Z_v(k)-(k/2,k/2)\Vert \le {\textstyle {\frac{T}{32n^4}}}$. This ensures that *v* satisfies the first condition required to be in $V_{n,T}$, but it also implies that

$$\begin{aligned} R(X_v(k),Y_v(k)) \le \frac{\frac{k}{2} + \frac{T}{32 n^4} + 1}{\frac{k}{2} - \frac{T}{32 n^4} + 1} = \frac{1+\frac{T}{32n^4(k/2+1)}}{1-\frac{T}{32n^4(k/2+1)}}, \end{aligned}$$

and so for $k\ge \frac{T}{4n^2}-2$,

34 $$\begin{aligned} R(X_v(k),Y_v(k)) \le \frac{1+\frac{1}{4n^2}}{1-\frac{1}{4n^2}} \le \frac{1}{(1-\frac{1}{4n^2})^2} \le \frac{1}{1-\frac{1}{2n^2}}, \end{aligned}$$

where we used the fact that $1+x\le 1/(1-x)$ for $x\in [0,1)$.

Now, ${\tilde{T}}_v(k)$ consists of a sum of *k* independent exponential random variables of parameter 1, which we call $\mathbbm {e}_v(0),\ldots ,\mathbbm {e}_v(k-1)$. For $k\ge \lfloor T/4n^2\rfloor$ we then have, by definition,

$$\begin{aligned} T_v(k) = \sum _{i=0}^{k-1} \frac{\mathbbm {e}_v(i)}{R(X_v(i),Y_v(i))} \ge \sum _{i=\lfloor T/4n^2\rfloor }^{k-1} \frac{\mathbbm {e}_v(i)}{R(X_v(i),Y_v(i))}. \end{aligned}$$

Applying (34), this is at least

$$\begin{aligned} \Big (1-\frac{1}{2n^2}\Big ) \sum _{i=\lfloor T/4n^2\rfloor }^{k-1} \mathbbm {e}_v(i) = \Big (1-\frac{1}{2n^2}\Big )\big ({\tilde{T}}_v(k) - {\tilde{T}}_v(\lfloor T/4n^2\rfloor )\big ). \end{aligned}$$

Since $v\in U_{n,T}$, whenever $k\le L(n,T)$ we have $|{\tilde{T}}_v(k) - k|\le T/4n^2$, and we obtain

$$\begin{aligned} T_v(k)  &   \ge \Big (1-\frac{1}{2n^2}\Big )\big (k-T/4n^2 - (\lfloor T/4n^2\rfloor + T/4n^2)\big )\\  &   \ge \Big (1-\frac{1}{2n^2}\Big )\Big (k-\frac{3T}{4n^2}\Big ) \ge k-\frac{7T}{8n^2}. \end{aligned}$$

We also obviously have $T_v(k) \ge 0 \ge k-\frac{7T}{8n^2}$ when $k<\lfloor T/4n^2\rfloor$; and since $R(x,y)\ge 1$ for all *x* and *y*, we have $T_v(k)\le {\tilde{T}}_v(k)$ for all *k*. Thus we have shown that if $v\in U_{n,T}$ then $|T_v(k)-k|\le 7T/8n^2$ for all $k\le L(n,T)$, and we deduce that also $v\in V_{n,T}$, as required. $\square$

We now want to move from discrete to continuous time. We need some more notation. For $v\in {\mathcal {N}}_t$ and $s\le t$, let *v*(*s*) be the unique ancestor of *v* that is in ${\mathcal {N}}_s$. Also let ${{\,\textrm{gen}\,}}(v)$ be the unique integer such that $v\in \mathbb {T}_{{{\,\textrm{gen}\,}}(v)}$.

#### Lemma 5.4

Recall the definition of $V_{n,T}$ from Lemma 5.3. If $v\in V_{n,T}$ then $v\in {\mathcal {N}}_t$ for some $t\ge \lceil n^{7/8}\rceil T/n + \lceil 2T/n^2\rceil - T/n^2$, and

$$\begin{aligned} \big |{{\,\textrm{gen}\,}}(v(s)) - s\big | \le \frac{7T}{8n^2} + 1 \end{aligned}$$

for all $$s\le \lceil n^{7/8}\rceil T/n + \lceil 2T/n^2\rceil - T/n^2 - 1$$.

#### Proof

If $v\in V_{n,T}$ then $|T_v(k)-k|\le 7T/8n^2$ for all $k\le L(n,T)$. In particular

$$\begin{aligned} T_v\ge L(n,T)-T/n^2 = \lceil n^{7/8}\rceil T/n + \lceil 2T/n^2\rceil - T/n^2, \end{aligned}$$

and therefore $v\in {\mathcal {N}}_t$ for some $t\ge \lceil n^{7/8}\rceil T/n + \lceil 2T/n^2\rceil - T/n^2$.

Now, for any $s\le \lceil n^{7/8}\rceil T/n + \lceil 2T/n^2\rceil - T/n^2 - 1$, since $v\in V_{n,T}$,

$$\begin{aligned} T_{v(s)} = T_v({{\,\textrm{gen}\,}}(v(s))) \ge {{\,\textrm{gen}\,}}(v(s)) - 7T/8n^2, \end{aligned}$$

so since $T_{v(s)}\le s$ (because $v(s)\in {\mathcal {N}}_s$) we have

35 $$\begin{aligned} {{\,\textrm{gen}\,}}(v(s))\le s+7T/8n^2. \end{aligned}$$

Since $s\le \lceil n^{7/8}\rceil T/n + \lceil 2T/n^2\rceil - T/n^2 - 1$, the above implies in particular that ${{\,\textrm{gen}\,}}(v(s))+1\le L(n,T) = {{\,\textrm{gen}\,}}(v)$ and therefore we also have (again since $v\in V_{n,T}$)

$$\begin{aligned} T_{v(s)}+\mathbbm {e}_{v(s)} = T_v({{\,\textrm{gen}\,}}(v(s))+1) \le {{\,\textrm{gen}\,}}(v(s))+1+7T/8n^2. \end{aligned}$$

Combining this with the fact that $T_{v(s)}+\mathbbm {e}_{v(s)} > s$ (because $v(s)\in {\mathcal {N}}_s$), we obtain

$$\begin{aligned} s<{{\,\textrm{gen}\,}}(v(s))+1+7T/8n^2, \end{aligned}$$

and combining this with (35) gives the result. $\square$

We can now prove the main result of this section.

#### Proof of Proposition 3.4

By Lemma 5.3, with probability at least $1-1/T^{3/2}$, we have $|V_{n,T}|\ge {\textstyle {\frac{1}{2T^2}}} 2^{L(n,T)}$. Suppose that $v\in V_{n,T}$ and let $t=\lceil n^{7/8}\rceil T/n$. Then by Lemma 5.4, ${{\,\textrm{gen}\,}}(v(t)) \ge t-7T/8n^2-1$, and of course ${{\,\textrm{gen}\,}}(v) = L(n,T) = \lceil n^{7/8}\rceil T/n + \lceil 2T/n^2\rceil$. Thus the number of descendants that *v*(*t*) has in $V_{n,T}$ is at most

$$\begin{aligned} 2^{\lceil n^{7/8}\rceil T/n + \lceil 2T/n^2\rceil - (\lceil n^{7/8}\rceil T/n - 7T/8n^2 - 1)} \le 2^{3T/n^2}. \end{aligned}$$

We deduce that if $|V_{n,T}|\ge \frac{1}{2T^2} 2^{L(n,T)}$ then the number of *distinct* ancestors of particles in $V_{n,T}$ that are in ${\mathcal {N}}_t$ must be at least

$$\begin{aligned} \frac{2^{L(n,T)}}{2T^2 \cdot 2^{3T/n^2}} \ge 2^{T/n^{1/8} - T/n^2 - 2\log _2 T - 1}. \end{aligned}$$

For $T\ge Cn^{48}$ and *C* large the right-hand side is certainly larger than $2^{T/n^{1/8} - 2T/n^2}$.

Now, if $u\in {\mathcal {N}}_t$ is an ancestor of a particle $v\in V_{n,T}$, and $s\le t$, then

$$\begin{aligned} \big \Vert Z_u(s) -(\tfrac{s}{2}, \tfrac{s}{2})\big \Vert&= \big \Vert Z_v(s)-(\tfrac{s}{2}, \tfrac{s}{2})\big \Vert \\&\le \big \Vert Z_v(s) - \big ({\textstyle {\frac{{{\,\textrm{gen}\,}}(v(s))}{2}}},{\textstyle {\frac{{{\,\textrm{gen}\,}}(v(s))}{2}}}\big )\big \Vert + \Vert \big ({\textstyle {\frac{{{\,\textrm{gen}\,}}(v(s))}{2}}},{\textstyle {\frac{{{\,\textrm{gen}\,}}(v(s))}{2}}}\big ) -(\tfrac{s}{2}, \tfrac{s}{2})\big \Vert \\&\le \frac{T}{32n^4} + \frac{1}{2}|{{\,\textrm{gen}\,}}(v(s))-s| \le \frac{T}{32n^4} + \frac{1}{2}\Big (\frac{7T}{8n^2} + 1\Big ) \end{aligned}$$

where for the first inequality we used the triangle inequality, for the second we used that $v\in V_{n,T}$, and for the third we again used that $v\in V_{n,T}$ together with Lemma 5.4. For $T\ge Cn^{48}$ and *C* large this is smaller than $T/2n^2$, which completes the proof.$\square$

### From the lead diagonal to other gradients: proof of Proposition 3.5

We will build up to the proof of Proposition 3.5 gradually, first constructing a suitable candidate function $h_{f,n}$, and then proving several lemmas that establish the required properties of $h_{f,n}$.

For $$\mu \ge \lambda >0$$ let

$$\begin{aligned} \kappa (\lambda ,\mu )&= \frac{\mu }{\lambda } - \Big (\sqrt{2}\Big (\frac{\mu }{\lambda }-\frac{1}{2}\Big )^{1/2} - \lambda ^{1/2}\Big )^2 - (1-\mu ^{1/2})^2\\&= -\frac{\mu }{\lambda } - \lambda - \mu + 2\mu ^{1/2} + 2(2\mu -\lambda )^{1/2}. \end{aligned}$$

We have defined $\kappa$ in such a way that, for $\mu \ge \lambda >0$, if $g(s)=(\lambda s,\mu s)$ for $s\in [0,1]$ then

$$\begin{aligned} K(g,0,t) = \kappa (\lambda ,\mu )t. \end{aligned}$$

We would like our function $h_{f,n}$ to begin with gradient (1/2, 1/2), but then to transition in small steps to having gradient $(f'_X(0),f'_Y(0))$. In order to ensure that $K(h_{f,n},0,t)$ remains positive for all small *t*, we need to check that $\kappa (\lambda ,\mu )$ is strictly positive for all the gradients $(\lambda ,\mu )$ that $h_{f,n}$ passes through at small times. If $\kappa$ was concave (or even concave on the region where it is positive) then this would be trivial since we could ask $h_{f,n}$ to transition linearly. Unfortunately there is a small region on which $\kappa$ is positive and not concave, so we have to use a more complicated argument. This is done in the following lemma.

#### Lemma 5.5

For every $0 < \lambda \le \mu$ such that $\kappa (\lambda , \mu )>0$, there exists a path $\gamma (t)=(\gamma _X(t), \gamma _Y(t))$, $t \in [0,1]$ and $\kappa _0>0$ such that

(i) $$(\gamma _X(0), \gamma _Y(0))=(1/2, 1/2)$$ and $$(\gamma _X(1), \gamma _Y(1))=(\lambda , \mu )$$;
(ii) $\kappa (\gamma (t)) \ge \kappa _0>0$ for all $t \in [0,1]$;
(iii) $\gamma$ is piecewise linear and $|\gamma _X'(t)|\le 20$ and $|\gamma _Y'(t)|\le 20$ for all $t \in [0,1]$ such that $\gamma$ is differentiable at *t*;
(iv) $\gamma _X(t) \in [ 3/2 - \sqrt{2}, 10]$ and $\gamma _Y(t) \in [ 3/2 - \sqrt{2}, 10]$ for all $t\in [0,1]$.

**Fig. 5:**
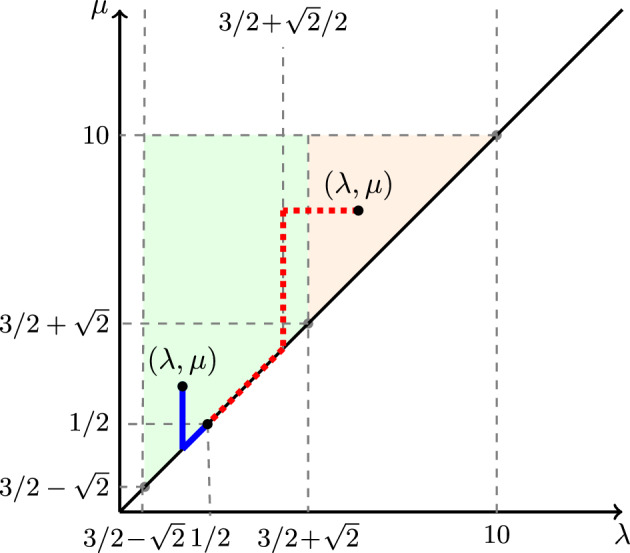
The pale green region is $\Upsilon _1$ and the pale orange region is $\Upsilon _2$. The thick blue (solid) and red (dotted) paths show our definition of $\gamma$ when $(\lambda , \mu )$ is in $\Upsilon _1$ and $\Upsilon _2$ respectively

#### Proof

We define $\Upsilon = \Upsilon _1\cup \Upsilon _2$ where

$$\begin{aligned} \Upsilon _1 = \{(\lambda ,\mu ): \lambda \in (3/2-\sqrt{2}, 3/2+\sqrt{2}),\, \mu \in [\lambda , 10)\} \end{aligned}$$

and

$$\begin{aligned} \Upsilon _2 = \{(\lambda ,\mu ): \mu \in (3/2+\sqrt{2}, 10),\, \lambda \in [3/2+\sqrt{2},\mu ]\}. \end{aligned}$$

Fig. 5 shows $\Upsilon _1$ and $\Upsilon _2$ in pale green and pale orange respectively. We show that the statement of the lemma holds for all the points ($\lambda , \mu ) \in \Upsilon$ and that $\kappa (\lambda , \mu )\le 0$ if $(\lambda , \mu ) \notin \Upsilon$. It is easy to see that

36 $$\begin{aligned} \kappa (\lambda ,\lambda )= -2\lambda +4 \sqrt{\lambda }-1>0 \,\,\,\,\text { for }\,\,\,\, \lambda \in (3/2-\sqrt{2}, 3/2+\sqrt{2}). \end{aligned}$$

and this is concave as a function of $\lambda$. Since for $0<\lambda \le \mu$ we have

$$\begin{aligned} \frac{\partial ^2 \kappa (\lambda , \mu )}{\partial \lambda ^2} = -(1/2)(2\mu -\lambda )^{-3/2} - (2 \mu )\lambda ^{-3/2} <0 \end{aligned}$$

and

$$\begin{aligned} \frac{\partial ^2 \kappa (\lambda , \mu )}{\partial \mu ^2} = -2(2\mu -\lambda )^{-3/2} - (1/2)\mu ^{-3/2} <0, \end{aligned}$$

the functions $\kappa (\cdot ,\mu )$ on $(0,\mu ]$ and $\kappa (\lambda ,\cdot )$ on $[\lambda ,\infty )$ are concave for each fixed $\lambda$ and $\mu$ respectively. This means that if we move parallel to either axis, we have the concave property; and so if for example $\kappa (\lambda _1,\mu )>0$ and $\kappa (\lambda _2,\mu )>0$ with $\lambda _1,\lambda _2\le \mu$, then $\kappa (\lambda ,\mu )>0$ for all $\lambda \in [\lambda _1,\lambda _2]$.

We now take advantage of this concavity parallel to the axes. For every $(\lambda , \mu ) \in \Upsilon _1$ such that $\kappa (\lambda , \mu )>0$ we choose $\gamma$ to be the union of the linear paths connecting (1/2, 1/2) to $(\lambda , \lambda )$ and then to $(\lambda , \mu )$.

Then clearly $\gamma$ satisfies (i) and (iv). Since $0<\lambda \le \mu \le 10$ throughout $\Upsilon$, the total length of the linear paths described is at most 20, and therefore we may choose a time parameterization of $\gamma$ such that $|\gamma _X'(t)|\le 20$ and $|\gamma _Y'(t)|\le 20$, so that $\gamma$ satisfies (iii). We claim that $\gamma$ also satisfies (ii). Indeed, by (36) $\kappa (\gamma (t))$ is positive on the first linear segment; in particular $\kappa (\lambda ,\lambda )>0$, and since by assumption $\kappa (\lambda ,\mu )>0$, by concavity parallel to the axes $\kappa (\gamma (t))$ is positive on the second linear segment too and $\kappa (\lambda ,\mu ) \ge \kappa _0$ where $\kappa _0:=\min \{ \kappa (1/2,1/2), \kappa (\lambda , \lambda ), \kappa (\lambda , \mu )\} >0$.

Now consider $(\lambda , \mu ) \in \Upsilon _2$ such that $\kappa (\lambda , \mu )>0$. Since $\lambda \ge 3/2+\sqrt{2}/2$ and $\mu <10$, we have

37 $$\begin{aligned} \frac{\partial \kappa (\lambda , \mu )}{\partial \lambda } = \frac{\mu }{\lambda ^2} - \frac{1}{\sqrt{2\mu -\lambda }}-1 \le \frac{4 \mu }{(3+\sqrt{2})^2} - \frac{1}{\sqrt{2\mu -\lambda }}-1 <0, \end{aligned}$$

so $\kappa (\lambda ', \mu )> \kappa (\lambda , \mu )>0$ for every $\lambda ' \in [3/2+\sqrt{2}/2, \lambda ]$. In particular, $\kappa (3/2+\sqrt{2}/2, \mu )>0$ and $(3/2+\sqrt{2}/2, \mu ) \in \Upsilon _1$, so we can define $\gamma$ as the union of the linear paths connecting (1/2, 1/2) to $(3/2+\sqrt{2}/2, 3/2+\sqrt{2}/2)$, then to $(3/2+\sqrt{2}/2, \mu )$, and then to $(\lambda , \mu )$. Then as above, $\gamma$ clearly satisfies (i) and (iv) and can be parameterized such that it satisfies (iii). Also $\kappa (\gamma (t))$ is positive on the first and second linear segments by the analysis of the $\lambda \in (3/2-\sqrt{2}, 3/2+\sqrt{2})$ case above, it is positive on the third linear segment by (37) and $\kappa (\lambda , \mu ) \ge \kappa _0$. Thus $\gamma$ satisfies (ii) too.

To complete our proof, it remains to show that $\kappa (\lambda , \mu )\le 0$ for $(\lambda ,\mu ) \notin \Upsilon$. First we note that $\kappa (3/2-\sqrt{2}, 3/2-\sqrt{2}) = 0$, and since for $\lambda \le 3/2-\sqrt{2}$ we have

$$\begin{aligned} \frac{\partial }{\partial \lambda }\kappa (\lambda ,3/2-\sqrt{2}) = \frac{3/2-\sqrt{2}}{\lambda ^2} - 1 - \frac{1}{(3-2\sqrt{2} -\lambda )^{1/2}} > 0, \end{aligned}$$

it follows that for every $\lambda < 3/2-\sqrt{2}$,

38 $$\begin{aligned} \kappa (\lambda , 3/2-\sqrt{2}) < 0. \end{aligned}$$

The fact that $\kappa (\lambda ,\mu )\le 0$ for $0<\lambda \le \mu \le 3/2-\sqrt{2}$ then follows by noticing that for every $t \in [0,3/2-\sqrt{2}-\mu ]$,

$$\begin{aligned} \frac{d}{dt} \kappa (\lambda +t,\mu +t)&= \left( \frac{\partial \kappa (\lambda , \mu )}{\partial \lambda } + \frac{\partial \kappa (\lambda , \mu )}{\partial \mu } \right) \bigg |_{(\lambda , \mu ) =(\lambda +t, \mu +t)} \\&=\frac{\mu -\lambda }{(\lambda +t)^2} + \frac{1}{\sqrt{2\mu +t-\lambda }} +\frac{1}{\sqrt{\mu +t}} -2 \\&\ge \frac{1}{\sqrt{3/2-\sqrt{2}}} -2 >0. \end{aligned}$$

Next, if $0<\lambda \le 3/2-\sqrt{2} < \mu$, then we use (38) plus the fact that

$$\begin{aligned} \frac{\partial \kappa (\lambda , \mu )}{\partial \mu } = -\frac{1}{\lambda } + \frac{2}{\sqrt{2 \mu -\lambda }} + \frac{1}{\sqrt{\mu }} -1 \le -\frac{1}{3/2-\sqrt{2}} + \frac{3}{\sqrt{3/2-\sqrt{2}}}-1 < 0. \end{aligned}$$

Finally, when $0< \lambda \le \mu$ and $\mu \ge 10$, the key fact is to observe that for every $\mu \ge 10$

39 $$\begin{aligned} \frac{\partial \kappa (\lambda , \mu )}{\partial \mu } = -\frac{1}{\lambda } + \frac{2}{\sqrt{2 \mu -\lambda }} + \frac{1}{\sqrt{\mu }} -1 \le -\frac{1}{\mu } + \frac{3}{\sqrt{\mu }}-1 < 0. \end{aligned}$$

Since $\kappa (\lambda , 10)<0$ for $\lambda \le 10$, (39) gives that $\kappa (\lambda , \mu )<0$ for any $\mu \ge 10$ and $\lambda \le 10$; and since $\kappa (\lambda ,\lambda )<0$ for $\lambda \ge 10$, (39) gives that $\kappa (\lambda ,\mu )<0$ whenever $\mu \ge 10$ and $\lambda \ge 10$. This completes the proof. $\square$

Take $f\in G_M^2$ such that $\frac{d}{dt} K(f,0,t)|_{t=0} > 0$ and $K(f,0,t)>0$ for all $t\in (0,1]$. Also fix $n\in \mathbb {N}$ and $m\in \mathbb {N}$ such that $n\ge m$. We now construct a function $h=h_{f,n,m}$ which depends on *n* and *m*; we will later show that for *m* sufficiently large (and *n* even larger) the resulting function satisfies the properties of Proposition 3.5.

Let $\tau = m^m \lceil n^{7/8}\rceil /n$. We will eventually choose *n* much larger than *m*, so that $\tau$ is small. Also let $\lambda = f'_X(0)$ and $\mu = f'_Y(0)$. Take $\gamma$ as in Lemma 5.5 and for $j\in \{0,1,\ldots ,m\}$ define

40 $$\begin{aligned} \lambda _j = \lambda _j^{(m)} = \gamma _X(j/m), \hspace{4mm} \mu _j = \mu _j^{(m)} = \gamma _Y(j/m)\hspace{2mm}\text { and }\hspace{2mm}\tau _j = \tau m^{j-m}. \end{aligned}$$

Begin by defining $h(s) = (s/2,s/2) = (\lambda _0 s,\mu _0 s)$ for $s\le \tau _0$. Then recursively, for each $j=1,\ldots ,m$, suppose that *h*(*s*) is defined for $s\le \tau _{j-1}$ and set

$$\begin{aligned} h(s) = h(\tau _{j-1}) + \big (\lambda _j(s-\tau _{j-1}),\,\mu _j(s-\tau _{j-1})\big ) \,\,\,\, \text { for } s\in (\tau _{j-1},\tau _j]. \end{aligned}$$

Also define

$$\begin{aligned} h(s) = h(\tau ) + \big (f(2\tau )-h(\tau )\big )\Big (\frac{s-\tau }{\tau }\Big ) \,\,\,\,\text { for } s\in (\tau ,2\tau ]. \end{aligned}$$

Finally, for each $j\in \{2\tau n,2\tau n+1,\ldots ,n\}$ let $h(j/n)=f(j/n)$ and interpolate linearly between these values.

Note that, since *K* has only downward jumps and $K(f,0,t)>0$ for all $t\in (0,1]$, we have $\inf _{s\in [\nu ,1]} K(f,0,s) > 0$ for every $\nu >0$. Thus we may choose $\nu = \nu _{f,m}\in (0,1]$ such that

- $$\Vert f(s)-(\lambda s,\mu s)\Vert \le s/m$$ for all $$s\le \nu $$,
- $K(f,s,t)>0$ for all $s\le \nu$ and $t\ge s$, and
- $$K(f,\nu ,1) \ge K(f,0,1)-1/m$$.

#### Lemma 5.6

Suppose that $\mu \ge \lambda >0$ and $\kappa (\lambda ,\mu )>0$. For any $m\ge 2$, $j\in \{1,\ldots ,m\}$ and any $s\in [\tau _{j-1},\tau _j]$,

$$\begin{aligned} \Vert h_{f,n,m}(s)-(\lambda _j s,\mu _j s)\Vert \le 40\tau _{j-1}/m. \end{aligned}$$

Moreover, if $2\tau \le \nu$, then for any $s\in [\tau ,\nu ]$,

$$\begin{aligned} \Vert h_{f,n,m}(s)-(\lambda s,\mu s)\Vert \le 40 s/m. \end{aligned}$$

#### Proof

We begin by noting that for $s\in [\tau _{j-1},\tau _j]$,

41 $$\begin{aligned} h(s)-(\lambda _j s,\mu _j s) = h(\tau _{j-1})-(\lambda _{j}\tau _{j-1},\mu _{j}\tau _{j-1}), \end{aligned}$$

so for the first part of the lemma it suffices to show that for any $j\in \{1,\ldots ,m\}$,

42 $$\begin{aligned} \Vert h(\tau _{j-1})-(\lambda _{j}\tau _{j-1},\mu _{j}\tau _{j-1})\Vert \le 40\tau _{j-1}/m. \end{aligned}$$

We prove (42) by induction. Recall that for each *j*, $\lambda _j = \gamma _X(j/m)$, and by Lemma 5.5 (iii), $|\lambda _{j-1} - \lambda _{j}|\le 20/m$ and $|\mu _{j}- \mu _{j-1} |\le 20/m$. Thus we first have

$$\begin{aligned} \Vert h(\tau _0)-(\lambda _1 \tau _0,\mu _1 \tau _0)\Vert = \max \{ |\lambda _0-\lambda _1 |\tau _0, |\mu _0-\mu _1 |\tau _0 \} \le 20 \tau _0/m. \end{aligned}$$

Suppose that $j\in \{1,\ldots ,m-1\}$ and (42) holds for *j*. By the triangle inequality,

$$\begin{aligned} |h_X(\tau _j)-\lambda _{j+1}\tau _j| \le |h_X(\tau _j) - \lambda _j\tau _j| + |\lambda _j\tau _j - \lambda _{j+1}\tau _j| \end{aligned}$$

and then by (41), this equals

$$\begin{aligned} |h_X(\tau _{j-1}) - \lambda _j\tau _{j-1}| + |\lambda _j-\lambda _{j+1}|\tau _j. \end{aligned}$$

Applying (42) and using the fact that $|\lambda _{j-1} - \lambda _{j}|\le 20/m$, we obtain that

$$\begin{aligned} |h_X(\tau _j)-\lambda _{j+1}\tau _j| \le \frac{40\tau _{j-1}}{m} + \frac{20\tau _j}{m} \le \frac{40\tau _j}{m}. \end{aligned}$$

By symmetry we also have $|h_Y(\tau _{j})-\mu _{j+1} \tau _{j}|\le 40\tau _j/m$. Hence, by induction, (42) holds for all $j\in \{1,\ldots ,m\}$, proving the first part of the lemma.

For the second part, suppose that $2\tau \le \nu$. Note first that for $s\in [\tau ,2\tau ]$, *h* is linear and therefore

$$\begin{aligned} \Vert h(s)-(\lambda s,\mu s)\Vert \le \max \big \{\Vert h(\tau )-(\lambda \tau ,\mu \tau )\Vert ,\,\Vert h(2\tau )-(2\lambda \tau ,2\mu \tau )\Vert \big \}. \end{aligned}$$

By the first part of the lemma,

$$\begin{aligned} \Vert h(\tau )-(\lambda \tau ,\mu \tau )\Vert \le 40\tau _{m-1}/m \le 40\tau /m \end{aligned}$$

and since $h(2\tau ) = f(2\tau )$, by property (a) of *f*,

$$\begin{aligned} \Vert h(2\tau )-(2\lambda \tau ,2\mu \tau )\Vert \le 2\tau /m. \end{aligned}$$

This proves the second part of the lemma for $s\in [\tau ,2\tau ]$; for $s\in [2\tau ,\nu ]$, we note that *h* linearly interpolates between values of *f*, and therefore for *j* such that $s\in [j/n,(j+1)/n]$,

$$\begin{aligned}  &   \Vert h(s)-(\lambda s,\mu s)\Vert \\  &   \hspace{5mm} \le \max \big \{\Vert f({\textstyle {\frac{j}{n}}})-(\lambda {\textstyle {\frac{j}{n}}},\mu {\textstyle {\frac{j}{n}}})\Vert ,\,\Vert f({\textstyle {\frac{j+1}{n}}})-(\lambda {\textstyle {\frac{j+1}{n}}},\mu {\textstyle {\frac{j+1}{n}}})\Vert \big \} \le \frac{j+1}{nm} \le \frac{s+1/n}{m} \le \frac{2s}{m} \end{aligned}$$

where we used (a) and the fact that $2\tau \ge 1/n$. $\square$

#### Corollary 5.7

Suppose that $\mu \ge \lambda >0$, $\kappa (\lambda ,\mu )>0$ and $m\ge 1600$, $m\in \mathbb {N}$. Then for any $j\in \{1,\ldots ,m\}$ and any $s\in [\tau _{j-1},\tau _j]$,

$$\begin{aligned} \frac{\mu _j}{\lambda _j}\Big (1-\frac{1600}{m}\Big ) \le R^*(h_{f,n,m}(s)) \le \frac{\mu _j}{\lambda _j}\Big (1+\frac{3200}{m}\Big ), \end{aligned}$$

and if $2\tau \le \nu$ then for any $s\in [\tau ,\nu ]$

$$\begin{aligned} \frac{\mu }{\lambda }\Big (1-\frac{1600}{m}\Big ) \le R^*_X(h_{f,n,m}(s))+1/2 \le R^*(h_{f,n,m}(s)) \le \frac{\mu }{\lambda }\Big (1+\frac{3200}{m}\Big ). \end{aligned}$$

#### Proof

We begin with the lower bound on $R^*(h(s))$ for $s\in [\tau _{j-1},\tau _j]$. Since $\mu \ge \lambda$, we have $h_Y(s)\ge h_X(s)$, and therefore by the first part of Lemma 5.6,

$$\begin{aligned} R^*(h(s)) = \frac{h_Y(s)}{h_X(s)} \ge \frac{\mu _j s - 40\tau _{j-1}/m}{\lambda _j s + 40\tau _{j-1}/m} = \frac{\mu _j}{\lambda _j}\Big (\frac{1 - 40\tau _{j-1}/(m\mu _j s)}{1 + 40\tau _{j-1}/(m\lambda _j s)}\Big ). \end{aligned}$$

Using the fact that $s\ge \tau _{j-1}$, and then that $1/(1+x)\ge 1-x$ for $x\ge 0$, this is at least

$$\begin{aligned} \frac{\mu _j}{\lambda _j}\Big (1 - \frac{40}{m\mu _j}\Big )\Big (1 - \frac{40}{m\lambda _j}\Big ). \end{aligned}$$

By Lemma 5.5 (iv), we have $\lambda _j\ge 3/2-\sqrt{2} \ge 1/20$ and similarly for $\mu _j$, so the above is at least $\frac{\mu _j}{\lambda _j}(1-1600/m)$, and the first lower bound on $R^*(h(s))$ follows. The first upper bound is similar, using that $1/(1-x) \le 1+2x$ for $x\in [0,1/2]$; since $m\ge 1600$ and $\lambda _j\ge 1/20$ we have $40/(m\lambda _j) \le 1/2$, and we obtain

$$\begin{aligned} R^*(h(s)) \le \frac{\mu _j}{\lambda _j}\Big (1 + \frac{40}{m\mu _j}\Big )\Big (1 + \frac{80}{m\lambda _j}\Big ); \end{aligned}$$

then since $\lambda _j\ge 1/20$, $\mu _j\ge 1/20$ and $m\ge 1600$, the product of the last two terms reduces to the desired form.

The proof of the second part of the corollary, when $s\in [\tau ,\nu ]$, is almost identical. Indeed, if $h_Y(s)\ge h_X(s)$ then $R^*_X(h(s))+1/2 = R^*(h(s))$ and we use the same argument but apply the second part of Lemma 5.6 rather than the first part. The same applies to the lower bound even when $h_Y(s)<h_X(s)$, since in any case $R^*_X(h(s)) +1/2 \ge h_Y(s)/h_X(s)$. However, we have to make a slight modification to the upper bound when $h_Y(s)<h_X(s)$; in this case, we instead have $R^*_X(h(s))+1/2 \le R^*(h(s))$ where $R^*(h(s)) = h_X(s)/h_Y(s)$, and then the argument above gives

$$\begin{aligned} R^*(h(s)) = \frac{h_X(s)}{h_Y(s)} \le \frac{\lambda s + 40s/m}{\mu s - 40s/m} \le \frac{\lambda }{\mu }\Big (1+\frac{40}{\lambda m}\Big )\Big (1+\frac{80}{\mu m}\Big ) \le \frac{\lambda }{\mu }\Big (1+\frac{3200}{m}\Big ). \end{aligned}$$

However, since $\lambda \le \mu$, we have $\lambda /\mu \le 1 \le \mu /\lambda$ and so the same conclusion holds. $\square$

#### Corollary 5.8

Suppose that $\mu \ge \lambda >0$, $\kappa (\lambda ,\mu )>0$ and $m\ge 1600$, $m\in \mathbb {N}$. There exists a finite constant *C* such that for any $j\in \{1,\ldots ,m\}$ and any $s,t\in [\tau _{j-1},\tau _j]$ with $s\le t$, we have

$$\begin{aligned} K(h_{f,n,m},s,t) \ge \kappa (\lambda _j,\mu _j)(t-s) - \frac{C}{m}(t-s), \end{aligned}$$

and if $2\tau \le \nu$ then for any $s,t\in [\tau ,\nu ]$ with $s\le t$, we have

$$\begin{aligned} K(h_{f,n,m},s,t) \ge \kappa (\lambda ,\mu )(t-s) - \frac{C}{m}(t-s). \end{aligned}$$

#### Proof

We begin with the first statement. Using (7), since $h(t)-h(s) = (\lambda _j(t-s),\mu _j(t-s))$, we have

$$\begin{aligned} K(h,s,t)= &   -\int \limits _s^t R^*(h(u))du + 2\sqrt{2} \int \limits _s^t \sqrt{R^*_X(h(u))\lambda _j}\, du\\  &   + 2\sqrt{2} \int \limits _s^t \sqrt{R^*_Y(h(u))\mu _j}\, du - \lambda _j(t-s) - \mu _j(t-s). \end{aligned}$$

Since $\mu \ge \lambda$, we have $h_Y(u)\ge h_X(u)$ for all $u\le \tau$ and therefore $R^*_X(h(u))=R^*(h(u))-1/2$ and $R^*_Y(h(u))=1/2$ for all $u\le \tau$. Thus

43 $$\begin{aligned} K(h,s,t)= &   -\int \limits _s^t R^*(h(u))du + 2\sqrt{2} \int \limits _s^t \sqrt{(R^*(h(u))-1/2)\lambda _j}\, du\nonumber \\  &   + 2\sqrt{\mu _j} (t-s) - \lambda _j(t-s) - \mu _j(t-s). \end{aligned}$$

By Corollary 5.7, for any $u\in [s,t]$ we have

$$\begin{aligned} \frac{\mu _j}{\lambda _j}\Big (1-\frac{1600}{m}\Big ) \le R^*(h(u)) \le \frac{\mu _j}{\lambda _j}\Big (1+\frac{3200}{m}\Big ) \end{aligned}$$

and, using also that $(1-x)^{1/2}\ge 1-x$ for $x\in [0,1)$,

$$\begin{aligned} \sqrt{(R^*(h(u))-1/2)}&\ge \bigg (\frac{\mu _j}{\lambda _j}\Big (1-\frac{1600}{m}\Big ) - 1/2\bigg )^{1/2} \\&= \Big (\frac{\mu _j}{\lambda _j}-\frac{1}{2}\Big )^{1/2}\Big (1-\frac{1600\mu _j}{m\lambda _j(\mu _j/\lambda _j-1/2)}\Big )^{1/2}\\&\ge \Big (\frac{\mu _j}{\lambda _j}-\frac{1}{2}\Big )^{1/2}\Big (1-\frac{3200}{m}\Big ). \end{aligned}$$

Substituting these estimates into (43), we have

$$\begin{aligned} K(h,s,t)\ge &   -\frac{\mu _j}{\lambda _j}\Big (1+\frac{3200}{m}\Big )(t-s) + 2\sqrt{2} \Big (\frac{\mu _j}{\lambda _j}-\frac{1}{2}\Big )^{1/2}\lambda _j^{1/2}\Big (1-\frac{3200}{m}\Big )(t-s)\\  &   + 2 \sqrt{\mu _j} (t-s) - \lambda _j(t-s) - \mu _j(t-s). \end{aligned}$$

Recognising that

$$\begin{aligned} \kappa (\lambda _j,\mu _j) = -\frac{\mu _j}{\lambda _j} + 2\sqrt{2}\Big (\frac{\mu _j}{\lambda _j}-\frac{1}{2}\Big )^{1/2}\lambda _j^{1/2} + 2\mu _j^{1/2}-\lambda _j-\mu _j, \end{aligned}$$

we see that

$$\begin{aligned} K(h,s,t)\ge &   \kappa (\lambda _j,\mu _j) (t-s)\\  &   \quad - \frac{3200\mu _j}{\lambda _j m}(t-s) - 2\sqrt{2} \Big (\frac{\mu _j}{\lambda _j}-\frac{1}{2}\Big )^{1/2}\lambda _j^{1/2}\frac{3200}{m}(t-s) \end{aligned}$$

and the first part of the result follows using Lemma 5.5 (iv).

The proof of the second part is almost identical, though since for $u\in [\tau ,\nu ]$ we do not have exactly $h'_X(u) = \lambda$ and $h'_Y(u)=\mu$, we must additionally use the bounds

$$\begin{aligned} h'_X(u) = \frac{f(2\tau )-h(\tau )}{\tau } \le \frac{2\lambda \tau + 2\tau /m^2 - \lambda \tau + 40\tau /m}{\tau } \le \lambda + \frac{42}{m} \end{aligned}$$

and similarly

$$\begin{aligned} h'_X(u) \ge \lambda - \frac{42}{m} \hspace{10mm} \text { and } \hspace{10mm} \mu - \frac{42}{m} \le h'_Y(u) \le \mu + \frac{42}{m}. \end{aligned}$$

With the addition of these estimates, the proof proceeds as before. $\square$

Corollary 5.8 essentially guarantees that $K(h_{f,n,m},s,t)$ is positive for $0\le s<t\le \nu$, provided that $\kappa (\lambda ,\mu )>0$. We now need to show that $K(h_{f,n,m},\nu ,t)$ is not too negative for $t\ge \nu$. The following result will be used to check that $K(f,\nu ,t)$ is closely approximated by $K(h_{f,n,m},\nu ,t)$.

#### Proposition 5.9

Suppose that $0\le s\le t\le 1$ and that $f\in G_M^2$. Let $f_n$ be the function in ${{\,\textrm{PL}\,}}_n$ constructed by setting $f_n(j/n)=f(j/n)$ for each $j=0,\ldots ,n$ and interpolating linearly. Then

$$\begin{aligned} \liminf _{n\rightarrow \infty }K(f_n,s,t) \ge K(f,s,t). \end{aligned}$$

We prove this in Appendix C.2. Later, in Lemma 7.2, we will also show that the opposite inequality holds in certain circumstances. We now have the ingredients to prove Proposition 3.5.

#### Proof of Proposition 3.5

As usual let $\lambda = f'_X(0)$, $\mu = f'_Y(0)$ and $\tau = m^m\lceil n^{7/8}\rceil /n$, with $\lambda _j$ and $\mu _j$ as in (40) and $\tau _j = \tau m^{j-m}$, for $j\in \{0,1,\ldots ,m\}$. We will check that $h_{f,n,m}$ satisfies the desired properties when *m* and *n* are sufficiently large. Without loss of generality we assume that $\mu \ge \lambda$.

Since $\tau _0 n$ is an integer we have $h_{f,n,m}\in {{\,\textrm{PL}\,}}_n^2$, and since $f\in G_M^2$ it is easy to see that $h_{f,n,m}\in G_M^2$ too. Since $\Vert h_{f,n,m}(s)\Vert \le Ms$ and $\Vert f(s)\Vert \le Ms$ for $s\le 2\tau = 2\,m^m\lceil n^{7/8}\rceil /n$, and $h_{f,n,m}(j/n)=f(j/n)$ for $j\ge 2\tau n$, by choosing *n* large enough that $2\tau M\le \varepsilon$ we have $h_{f,n,m}\in B(f,\varepsilon )$. This proves that $h_{f,n,m}$ satisfies (11) when *n* is large.

For (12), note first that $\tau _0 = \lceil n^{7/8}\rceil /n$. Take *n* large enough that $2\tau <\nu$. Then we claim that since $\lim _{t\rightarrow 0}K(f,0,t)/t > 0$, we have $\kappa (\lambda ,\mu )>0$. To see why the claim holds, for small *s* we have $\Vert f'(s)-(\lambda ,\mu )\Vert \le 1/m$ and $\Vert f(s)-(\lambda s,\mu s)\Vert \le s/m$. The same argument as in Corollary 5.7 then shows that for some finite constant *C*,

$$\begin{aligned} \frac{\mu }{\lambda }\Big (1-\frac{C}{m}\Big ) \le R^*_X(f(s)) + \frac{1}{2} \le R^*(f(s)) \le \frac{\mu }{\lambda }\Big (1+\frac{C}{m}\Big ) \end{aligned}$$

and plugging these estimates into (7) with $a=0$ and $b=t$ and using standard approximations shows that $K(f,0,t) \le \kappa (\lambda ,\mu )t + C't/m$ for some finite constant $C'$. This implies the claim.

Since $\kappa (\lambda , \mu )>0$, by Lemma 5.5 we may choose $\kappa _0>0$ such that $\kappa (\gamma (t))\ge \kappa _0$ for all $t\in [0,1]$, and then $\kappa (\lambda _j,\mu _j)\ge \kappa _0$ for all $j\in \{0,\ldots ,m\}$. Corollary 5.8 then tells us that for $s\in [\tau _{j-1},\tau _j]$ we have

$$\begin{aligned}&K(h_{f,n,m},\tau _0,s) \\&\ge \sum _{i=1}^{j-1}(\kappa (\lambda _i,\mu _i)-C/m)(\tau _i-\tau _{i-1}) + (\kappa (\lambda _j,\mu _j)-C/m)(s-\tau _{j-1})\\&\ge (\kappa _0-C/m)(s-\tau _0), \end{aligned}$$

and for $s\in [\tau ,\nu ]$ we have

$$\begin{aligned} K(h_{f,n,m},\tau _0,s)&\ge \sum _{i=1}^{m}(\kappa (\lambda _i,\mu _i)-C/m)(\tau _i-\tau _{i-1}) + (\kappa (\lambda ,\mu )-C/m)(s-\tau )\\&\ge (\kappa _0-C/m)(s-\tau _0). \end{aligned}$$

Thus, by choosing *m* large enough, we have $K(h_{f,n,m},\tau _0,s)\ge \kappa _0(s-\tau _0)/2$ for all $s\in [\tau _0,\nu ]$.

For $s>\nu$, by the above argument we have

44 $$\begin{aligned}  &   K(h_{f,n,m},\tau _0,s) \nonumber \\  &   \ge K(h_{f,n,m},\tau _0,\nu ) + K(h_{f,n,m},\nu ,s) \ge \kappa _0(\nu -\tau _0)/2 + K(h_{f,n,m},\nu ,s),\nonumber \\ \end{aligned}$$

and since $\kappa _0(\nu -\tau _0)/2$ increases to $\kappa _0\nu /2$ as $n\rightarrow \infty$ and $K(f,\nu ,s)>0$ by (b), to show (12) it suffices to show that for large *n*, $K(h_{f,n,m},\nu ,s) \ge K(f,\nu ,s) - \kappa _0\nu /4$. But since *h* is the piecewise linear interpolation of *f* on the interval $[\nu ,1]$, this follows from Proposition 5.9.

Finally, for (13), applying (44) with $s=1$, we certainly have

$$\begin{aligned} K(h_{f,n,m},\tau _0,1) \ge K(h_{f,n,m},\nu ,1); \end{aligned}$$

by Proposition 5.9 the right-hand side converges to $K(f,\nu ,1)$ as $n\rightarrow \infty$; and by (c) we know that $K(f,\nu ,1) \ge K(f,0,1)-1/m$. This completes the proof.

## Coupling $\xi ^T$ with simpler processes

One problem we face is that $\xi _X$ and $\xi _Y$ are not independent, because their jump rates at time *t* are functions of the pair $(\xi _X(t),\xi _Y(t))$. However, if we already know that $\xi ^T$ has remained near a fixed function *f*, then the jump rates are “almost deterministic” and therefore $\xi _X$ and $\xi _Y$ are “almost independent”. In order to take advantage of this idea, we will construct new processes $Z_+$ and $Z_-$ which have the maximal and minimal jump rates (respectively) that $\xi ^T$ may have if it remains near *f*. We will couple these processes with another process, *Z*, which will have the same distribution as $\xi ^T$ but will be trapped between $Z_+$ and $Z_-$, as long as *Z* remains near *f*.

Recall the definitions of $R_X^-(I,F,T)$, $R_X^+(I,F,T)$, $R_Y^-(I,F,T)$, $R_Y^+(I,F,T)$, |*I*|, $I^+$, $I^-$, $x^-(s,F)$, $x^+(s,F)$, $y^-(s,F)$, $y^+(s,F)$, $\Gamma _{M,T}(f,n)$ and $I_j$ from Sect. 2.1. In what follows, the reader can think of the case $I=I_j$ and $F=\Gamma _{M,T}(f,n)$ for some function *f*.

Let

$$\begin{aligned}V(I,F) = [x^-(I^-,F),x^+(I^-,F)]\times [y^-(I^-,F),y^+(I^-,F)].\end{aligned}$$

Take $z=(x,y)\in V(I,F)$. Under a probability measure $Q_z = Q^{I,F,T}_z$, let $(X_+(I^-+s), s\in |I|)$ be a compound Poisson process started from *x* with rate $2R_X^+(I,F,T)T$ and jumps that are exponentially distributed with parameter *T*, and let $(Y_+(I^-+s), s\in |I|)$ be an independent compound Poisson process started from *y* with rate $2R_Y^+(I,F,T)T$ and jumps that are exponentially distributed with parameter *T*. Let $Z_+ = (X_+,Y_+)$.

We now construct—again under $Q_z^{I,F,T}$—two more (pure jump) processes $Z(I^-+s)$ and $Z_-(I^-+s)$ for $s\in |I|$ recursively as follows. Start by setting $Z(I^-)=z$ and $Z_-(I^-) = z$. The jumps of both *Z* and $Z_-$ are subsets of the jumps of $Z_+$. Suppose that $Z_+$ has a jump at time *s*, and that $Z(s-) = z'$. Let *U* be an independent Uniform[0, 1] random variable. Since $X_+$ and $Y_+$ are independent, exactly one of $X_+$ or $Y_+$ jumps at time *s*. Suppose for a moment that $X_+$ has a jump of size $x'>0$. Then accept the jump for *Z* if $U\le R_X(z')/R_X^+(I,F,T)$ and reject it otherwise; in other words, set $Z(s) = z'+(x',0)$ with probability $R_X(z')/R_X^+(I,F,T)$ and $Z(s) = z'$ otherwise. Accept the jump for $Z_-$ if $U\le R_X^-(I,F,T)/R_X^+(I,F,T)$ and reject it otherwise. Similarly, if $Y_+$ has a jump of size $y'>0$, then accept the jump for *Z* if $U\le R_Y(z')/R_Y^+(I,F,T)$, and accept it for $Z_-$ if $U\le R_Y^-(I,F,T)/R_Y^+(I,F,T)$.

More formally, we take homogeneous Poisson processes $(n_s^X)_{s\ge 0}$ and $(n_s^Y)_{s\ge 0}$ of rates $2R^+_X(I,F,T)T$ and $2R^+_Y(I,F,T)T$ respectively; two independent sequences $(\mathcal X_j)_{j\in \mathbb {N}}$ and $({\mathcal {Y}}_j)_{j\in \mathbb {N}}$ of independent Exponential random variables of parameter *T*, and two independent sequences $(U^X_j)_{j\in \mathbb {N}}$ and $(U^Y_j)_{j\in \mathbb {N}}$ of independent uniform random variables on (0, 1). We define $Z_+= (X_+,Y_+)$ by setting

$$\begin{aligned} X_+(I^- + s) = \sum _{j=1}^{n^X_s}{\mathcal {X}}_j \,\,\,\,\text { and } \,\,\,\, Y_+(I^- + s) = \sum _{j=1}^{n^Y_s}{\mathcal {Y}}_j. \end{aligned}$$

Then to define $Z_- = (X_-,Y_-)$, let $u^-_X = \frac{R^-_X(I,F,T)}{R^+_X(I,F,T)}$ and $u^-_Y = \frac{R^-_Y(I,F,T)}{R^+_Y(I,F,T)}$ and set

$$\begin{aligned} X_-(I^-+s) = \sum _{j=1}^{n^X_s}{\mathcal {X}}_j\mathbbm {1}_{\{U_j^X \le u^-_X\}}\,\,\,\,\text { and } \,\,\,\, Y_-(I^- + s) = \sum _{j=1}^{n^Y_s}{\mathcal {Y}}_j\mathbbm {1}_{\{U_j^Y \le u^-_Y\}}. \end{aligned}$$

To define $Z=(X,Y)$, let ${\mathcal {T}}_j = \inf \{t\ge 0: n_s^X + n_s^Y \ge j\}$, the *j*th jump time of the combined Poisson process $n_s^X+n_s^Y$. In particular ${\mathcal {T}}_0=0$. Define $X(I^-+s)=Y(I^-+s)=0$ for $s\in [{\mathcal {T}}_0,{\mathcal {T}}_1)$, and recursively for each $i\in \mathbb {N}$ set

$$\begin{aligned} u_X(j) = \frac{R_X(X(I^-+{\mathcal {T}}_{i-1}),Y(I^-+{\mathcal {T}}_{i-1}))}{R_X^+(I,F,T)} \end{aligned}$$

and

$$\begin{aligned} u_Y(j) = \frac{R_Y(X(I^-+{\mathcal {T}}_{i-1}),Y(I^-+{\mathcal {T}}_{i-1}))}{R_Y^+(I,F,T)} \end{aligned}$$

and for $$s\in [{\mathcal {T}}_i,{\mathcal {T}}_{i+1})$$,

$$\begin{aligned} X_-(I^-+s) = \sum _{j=1}^{n^X_s}{\mathcal {X}}_j\mathbbm {1}_{\{U_j^X \le u_X(j)\}}\,\,\,\,\text { and } \,\,\,\, Y_-(I^- + s) = \sum _{j=1}^{n^Y_s}{\mathcal {Y}}_j\mathbbm {1}_{\{U_j^Y \le u_X(j)\}}. \end{aligned}$$

Now, recall that for $F\subset E^2$, $g\in E$ and an interval $I\subset [0,1]$, we say that $g|_I\in F|_I$ if there exists a function $h\in F$ such that $h(u)=g(u)$ for all $u\in I$. Let

$$\begin{aligned} {\mathcal {A}}_\xi (I,F,T) = \big \{\xi ^T|_I \in F|_I\big \} \hspace{10mm} \text { and } \hspace{10mm} {\mathcal {A}}(I,F,T) = \big \{Z|_I \in F|_I\big \}. \end{aligned}$$

Note that for any $z\in V(I,F)$, on the event ${\mathcal {A}}(I,F,T)$, under $Q_z^{I,F,T}$ we always have

$$\begin{aligned}  &   R_X(Z(s))\in [R_X^-(I,F,T),R_X^+(I,F,T)] \\  &   \hspace{2mm}\text { and }\hspace{2mm} R_Y(Z(s))\in [R_Y^-(I,F,T),R_Y^+(I,F,T)], \end{aligned}$$

for all $s\in I$. Thus, by our construction:

(i) under $Q^{I,F,T}_z$, on the event ${\mathcal {A}}(I,F,T)$, we have $X_-(s) \le X(s)\le X_+(s)$ and $Y_-(s)\le Y(s)\le Y_+(s)$ for all $s\in I$;
(ii) the process $(Z(s)\mathbbm {1}_{{\mathcal {A}}(I\cap [0,s],F,T)})_{s\in I}$ under $Q_z^{I,F,T}$ is equal in distribution to the process $(\xi ^T(s)\mathbbm {1}_{{\mathcal {A}}_\xi (I\cap [0,s],F,T)})_{s\in I}$ conditionally on $\xi ^T(I^-)=z$ under $\mathbb {Q}$;
(iii) under $Q^{I,F,T}_z$, the processes $(X_-,X_+)$ and $(Y_-,Y_+)$ are independent.

Furthermore, by the thinning property of Poisson processes,

(i) under $Q^{I,F,T}_z$, the processes $X_-$ and $X_+-X_-$ are independent, as are $Y_-$ and $Y_+-Y_-$.

### Applying the coupling to the upper bound: proof of Propositions 2.3 and 3.11

Recall the terminology “$X+$ case” and “$X-$ case” from Sect. 2.1, and the definitions of $\mathcal E^+_X(I,F,T)$ and ${\mathcal {E}}^+_Y(I,F,T)$. The main part of the proof of Proposition 2.3 is the following lemma, whose proof is a completely standard application of Chernoff bounds, which we include for completeness in Appendix B.

#### Lemma 6.1

Suppose that $F\subset E^2$ and $T>1$. Then for any $I\subset [0,1]$ and $z\in V(I,F)$,

$$\begin{aligned} \mathbb {Q}\big ({\mathcal {A}}_\xi (I,F,T) \,\big |\,\xi ^T(I^-)=z\big ) \le \exp \big (-T{\mathcal {E}}^+_X(I,F,T)-T{\mathcal {E}}^+_Y(I,F,T)\big ). \end{aligned}$$

Our main results in this section are now easy corollaries of Lemma 6.1.

#### Proof of Proposition 2.3

Recall that $I_j = [j/n,(j+1)/n]$ and define $V(j) = V(I_j,\Gamma _{M,T}(f,n))$. Note that the restrictions on *z* ensure that $z\in V(i)$, and therefore by the Markov property,

$$\begin{aligned}&\mathbb {Q}\big (\xi ^T|_{[i/n,\theta ]}\in \Gamma _{M,T}(f,n)\big |_{[i/n,\theta ]}\,\big |\,\xi ^T_{i/n} = z\big ) \\&\quad \le \prod _{j=i}^{\lfloor \theta n\rfloor -1} \sup _{z'\in V(j)} \mathbb {Q}\big (\xi ^T|_{I_j}\in \Gamma _{M,T}(f,n)|_{I_j} \,\big |\, \xi ^T_{j/n} = z'\big )\\&\quad = \prod _{j=i}^{\lfloor \theta n\rfloor -1} \sup _{z'\in V(j)} \mathbb {Q}\big ({\mathcal {A}}_\xi (I_j,\Gamma _{M,T}(f,n),T) \,\big |\, \xi ^T_{j/n} = z'\big ). \end{aligned}$$

The result now follows from Lemma 6.1.

#### Proof of Proposition 3.11

Let $i=\lfloor an\rfloor$ and $\ell =\lceil bn\rceil$. Let $V_i = \{w: \Vert w-f(a)\Vert <1/n^2\}$, and for $j \in \{i+1,\ldots ,\ell \}$ let $V_j = \{w: \Vert w-f(j/n)\Vert <1/n^2\}$. Note that, by the Markov property,

$$\begin{aligned}&\mathbb {Q}\big (\xi ^T|_{[a,b]}\in \Lambda _{M,T}(f,n)\big |_{[a,b]}\,\big |\,\xi ^T_a = z\big ) \\&\quad \le \prod _{j=i}^{\ell -1} \sup _{w\in V_j} \mathbb {Q}\big (\xi ^T|_{I_j\cap [a,b]}\in \Lambda _{M,T}(f,n)\big |_{I_j\cap [a,b]}\,\big |\,\xi ^T_{j/n} = w\big )\\&\quad = \prod _{j=i}^{\ell -1} \sup _{w\in V_j} \mathbb {Q}\big (\mathcal A_\xi (I_j\cap [a,b],\Lambda _{M,T}(f,n),T) \,\big |\, \xi ^T_{j/n} = w\big ). \end{aligned}$$

The result now follows from Lemma 6.1, together with (16) and (17).$\square$

### Applying the coupling to the lower bound: proofs of Lemmas 3.6, 3.7 and 3.8

We begin this section with the proof of Lemma 3.6, which links the probability that we want to bound with our coupled compound Poisson processes.

#### Proof of Lemma 3.6

We begin by splitting [0, 1] into its subintervals $I_j$, $j=0,\ldots ,n-1$. By applying the Markov property at each time *j*/*n*,

$$\begin{aligned}&\mathbb {Q}\Big (\xi ^T|_{[k/n,1]}\in \Lambda _{M,T}(f,n)|_{[k/n,1]}\,\Big |\, \xi ^T(k/n) = w\Big )\\&\quad \ge \mathbb {Q}\Big ( \Vert \xi ^T(s)-f(s)\Vert<1/n^2 \;\;\forall s\in I_j, \ \xi ^T\big ({\textstyle {\frac{j+1}{n}}} \big )\in {\mathcal {Z}}_{j+1}, \\&\qquad \qquad \quad \xi ^T|_{I_j}\in G_{M,T}^2|_{I_j} \; \; \forall j\in \{k,\ldots ,n-1\} \Big |\, \xi ^T\big ({\textstyle {\frac{k}{n}}}\big ) = w\Big )\\&\quad \ge \prod _{j=k}^{n-1} \inf _{z \in {\mathcal {Z}}_j} \mathbb {Q}\Big ( \Vert \xi ^T(s)-f(s)\Vert <1/n^2 \;\; \forall s\in I_j, \ \xi ^T\big ({\textstyle {\frac{j+1}{n}}}\big )\in {\mathcal {Z}}_{j+1}, \\&\qquad \qquad \qquad \qquad \xi ^T|_{I_j}\in G_{M,T}^2|_{I_j} \Big |\, \xi ^T\big ({\textstyle {\frac{j}{n}}}\big )=z\Big ). \end{aligned}$$

It therefore remains to show that for each *j* and any $z\in {\mathcal {Z}}_j$,

45 $$\begin{aligned}  &   \mathbb {Q}\Big ( \big \Vert \xi ^T(s)-f(s)\big \Vert <1/n^2 \;\; \forall s\in I_j, \; \xi ^T\big ({\textstyle {\frac{j+1}{n}}}\big )\in {\mathcal {Z}}_{j+1}, \; \xi ^T|_{I_j}\in G_{M,T}^2|_{I_j} \,\Big |\, \xi ^T\big ({\textstyle {\frac{j}{n}}}\big )=z\Big )\nonumber \\  &   \quad \ge q^X_{n,M,T}(z,j,f)\, {\hat{q}}^X_{n,M,T}(z,j,f)\, q^Y_{n,M,T}(z,j,f)\, {\hat{q}}^Y_{n,M,T}(z,j,f). \end{aligned}$$

We now use the coupling from Sect. 6, with $I=I_j$ and $F=\Lambda _{M,T}(f,n)$. We simply write $Q_z$ as shorthand for $Q_z^{I_j,\Lambda _{M,T}(f,n),T}$. By property (ii) of the coupling, we have

$$\begin{aligned}  &   \mathbb {Q}\Big ( \big \Vert \xi ^T(s)-f(s)\big \Vert <1/n^2 \;\; \forall s\in I_j, \; \xi ^T\big ({\textstyle {\frac{j+1}{n}}}\big )\in {\mathcal {Z}}_{j+1}, \; \xi ^T|_{I_j}\in G_{M,T}^2|_{I_j} \,\Big |\, \xi ^T\big ({\textstyle {\frac{j}{n}}}\big )=z\Big )\\  &   = Q_z\Big ( \big \Vert Z(s)-f(s)\big \Vert \le 1/n^2\;\;\forall s\in I_j, \; Z\big ({\textstyle {\frac{j+1}{n}}}\big )\in {\mathcal {Z}}_{j+1}, \; Z|_{I_j}\in G_{M,T}^2|_{I_j}\Big ) \end{aligned}$$

which, by property (i), is at least

$$\begin{aligned}  &   Q_z\Big (\big \Vert Z_-(s)- f(s)\big \Vert \le {\textstyle {\frac{1}{n^2}}} \,\,\forall s\in I_j, \\  &   \qquad Z_-\big ({\textstyle {\frac{j+1}{n}}}\big )\in {\mathcal {Z}}_{j+1}, \, Z_-|_{I_j}\in G_{M,T}^2|_{I_j},\, Z_+(s)-Z_-(s) =0\,\,\forall s\in I_j\Big ). \end{aligned}$$

By property (iv), this equals

$$\begin{aligned}  &   Q_z\Big (\big \Vert Z_-(s)- f(s)\big \Vert \le {\textstyle {\frac{1}{n^2}}} \;\;\forall s\in I_j, \; Z_-\big ({\textstyle {\frac{j+1}{n}}}\big )\in {\mathcal {Z}}_{j+1}, \; Z_-|_{I_j}\in G_{M,T}^2|_{I_j}\Big )\\  &   \cdot Q_z\Big ( Z_+(s)-Z_-(s) =0\;\;\forall s\in I_j\Big ) \end{aligned}$$

and finally, by property (iii), the above equals

$$\begin{aligned}  &   Q_z\Big (\big |X_-(s)- f_X(s)\big | \le {\textstyle {\frac{1}{n^2}}} \;\forall s\in I_j,\, \big |X_-\big ({\textstyle {\frac{j+1}{n}}}\big )-f_X\big ({\textstyle {\frac{j+1}{n}}}\big )\big | \le {\textstyle {\frac{1}{2n^2}}}, \, X_-|_{I_j}\in G_{M,T}|_{I_j}\Big )\\  &   \cdot Q_z\Big (\big |Y_-(s)- f_Y(s)\big | \le {\textstyle {\frac{1}{n^2}}} \;\forall s\in I_j,\, \big |Y_-\big ({\textstyle {\frac{j+1}{n}}}\big )- f_Y\big ({\textstyle {\frac{j+1}{n}}}\big )\big | \le {\textstyle {\frac{1}{2n^2}}}, \, Y_-|_{I_j}\in G_{M,T}|_{I_j}\Big )\\  &   \cdot Q_z\Big ( X_+(s)-X_-(s) =0\;\forall s\in I_j\Big ) \cdot Q_z\Big ( Y_+(s)-Y_-(s) =0\;\forall s\in I_j\Big ). \end{aligned}$$

Noting that $X_+-X_-$ and $Y_+-Y_-$ are increasing, this is exactly

$$\begin{aligned} q^X_{n,M,T}(z,j,f)\, {\hat{q}}^X_{n,M,T}(z,j,f)\, q^Y_{n,M,T}(z,j,f)\, {\hat{q}}^Y_{n,M,T}(z,j,f). \end{aligned}$$

Thus we have shown (45) and the proof is complete.$\square$

The proof of Lemma 3.7, which bounds the ${\hat{q}}$ terms, is elementary.

#### Proof of Lemma 3.7

Recall that

$$\begin{aligned} {\hat{q}}^X_{n,M,T}(z,j,f) = Q_z^{I_j,\Lambda _{M,T}(f,n),T}\Big ( X_+\big ({\textstyle {\frac{j+1}{n}}}\big )-X_-\big ({\textstyle {\frac{j+1}{n}}}\big ) =0\Big ). \end{aligned}$$

Also recall that under $Q_z^{I_j,\Lambda _{M,T}(f,n),T}$, the process $X_+ - X_-$ jumps at rate

$$\begin{aligned} 2\big (R_X^+(I_j,\Lambda _{M,T}(f,n),T) - R_X^-(I_j,\Lambda _{M,T}(f,n),T)\big )T. \end{aligned}$$

Therefore, for each $j\in \{0,\ldots ,n-1\}$ and $z\in {\mathcal {Z}}_j$, using (17),

$$\begin{aligned} {\hat{q}}^X_{n,M,T}(z,j,f) \ge \exp \Big (-2\big (R_X^+(I_j,\Gamma _{M,T}(f,n),T) - R_X^-(I_j,\Gamma _{M,T}(f,n),T)\big )T/n\Big ). \end{aligned}$$

Since $f\in G_M^2$, for $j\ge \sqrt{n}$, by (18) we have ${\hat{q}}^X_{n,M,T}(z,j,f) \ge \exp \big (-4\delta _{M,T}(j,n)T/n\big )$, and by symmetry the same bound holds for ${\hat{q}}^Y_{n,M,T}(z,j,f)$. The result then follows from (60).

The proof of Lemma 3.8 is much more delicate. Our next result provides a bound on compound Poisson processes, which we prove using standard arguments in Appendix B. This will then be applied to prove Lemma 3.8.

#### Lemma 6.2

Suppose that $\delta ,t,A>0$ and $a\in \mathbb {R}$ satisfy $a<tA/2$ and $|a|\le \delta /2$. Suppose also that $R\ge 1/2$. Let $(X(s),s\ge 0)$ be a compound Poisson process of rate *RT* whose jumps are exponentially distributed with parameter *T*. Then for $T>\frac{2(A-a/t)^{3/2}(4t+\delta )}{R^{1/2}\delta ^{2}((A-a/t)\wedge 1)^2}$,

$$\begin{aligned}  &   \mathbb {P}(|a+X(s)-As|<\delta \,\, \forall s\le t, \,\, |a+X(t)-At|<\delta /2)\\  &   \ge \frac{1}{2}\exp \Big ( \! -tT(\sqrt{R} - \sqrt{A})^2 - \delta \Big (1+\sqrt{R}\big (\sqrt{2t/\delta }+1/2\big )\Big )T\Big ). \end{aligned}$$

We now apply Lemma 6.2 to prove Lemma 3.8. However we still need to consider two cases: if $f_X$ does not change much over the interval $I_j$ then we may simply ask our process not to jump over that interval, and a bound similar to that in the proof of Lemma 3.7 is better than the estimate provided by Lemma 6.2.

#### Proof of Lemma 3.8

Recall that

$$\begin{aligned} q^X_{n,3M,T}(z,j,f)= &   Q_z^{I_j,\Lambda _{3M,T}(f,n),T}\Big (\big |X_-(s)- f_X(s)\big | \le {\textstyle {\frac{1}{n^2}}} \,\,\forall s\in I_j,\\  &   \big |X_-({\textstyle {\frac{j+1}{n}}})-f_X({\textstyle {\frac{j+1}{n}}})\big | \le {\textstyle {\frac{1}{2n^2}}},\, X_-|_{I_j}\in G_{3M,T}|_{I_j}\Big ) \end{aligned}$$

Write $Q_z$ as shorthand for $Q_z^{I_j,\Lambda _{3M,T}(f,n),T}$.

Since $f\in G_M^2$, $j\ge 1$ and $n\ge 2\,M$, under $Q_z$ we also have, for any $s\in I_j$,

$$\begin{aligned} X_-(s)\ge x \ge f_X \Big (\frac{j}{n}\Big )-\frac{1}{2n^2} \ge \frac{j}{Mn} - \frac{1}{2n^2} \ge \frac{3j}{3Mn} - \frac{1}{3Mn}&\ge \frac{j+2}{3Mn} - \frac{1}{3Mn} \\&= \frac{j+1}{3Mn} \ge \frac{s}{3M}, \end{aligned}$$

and if $\big |X_-({\textstyle {\frac{j+1}{n}}})-f_X({\textstyle {\frac{j+1}{n}}})\big | \le {\textstyle {\frac{1}{2n^2}}}$ then also

$$\begin{aligned} X_-(s)\le X_-({\textstyle {\frac{j+1}{n}}}) \le f_X({\textstyle {\frac{j+1}{n}}}) + {\textstyle {\frac{1}{2n^2}}} \le M{\textstyle {\frac{j+1}{n}}} + {\textstyle {\frac{1}{2n^2}}} \le 3Ms. \end{aligned}$$

Thus in fact, under the conditions of the lemma, $X_-|_{I_j}$ is always in $G_{3M,T}|_{I_j}$, so

46 $$\begin{aligned} q^X_{n,3M,T}(z,j,f)&= Q_z^{I_j,\Lambda _{3M,T}(f,n),T}\Big ( \big |X_-(s)- f_X(s)\big | \le {\textstyle {\frac{1}{n^2}}} \;\;\forall s\in I_j,\nonumber \\&\quad \qquad \big |X_-({\textstyle {\frac{j+1}{n}}})-f_X({\textstyle {\frac{j+1}{n}}})\big | \le {\textstyle {\frac{1}{2n^2}}}\Big ). \end{aligned}$$

For the remainder of this proof, for $I\subset [0,1]$, we write $\hat{R}^-_X(I)$ as shorthand for the quantity $R^-_X(I,\Lambda _{M,T}(f,n),T)$, and similarly for ${\hat{R}}^+_X(I)$, ${\hat{R}}^-_Y(I)$ and ${\hat{R}}^+_Y(I)$. (Recall that we wrote $R^-_X(I)$ in Sect. 6 to mean $R^-_X(I,\Gamma _{M,T}(f,n),T)$.)

**Case 1:** $$f_X(\frac{j+1}{n})\le x + \frac{1}{2n^2}$$.

Note that since $z\in {\mathcal {Z}}_j$, we have $x\le f_X({\textstyle {\frac{j}{n}}})+{\textstyle {\frac{1}{2n^2}}} \le f_X({\textstyle {\frac{j+1}{n}}})+{\textstyle {\frac{1}{2n^2}}}$, and therefore $|x-f_X(s)|\le {\textstyle {\frac{1}{2n^2}}}$ for all $s\in I_j$. Thus (46) can be bounded in the following trivial way:

$$\begin{aligned} q^X_{n,3M,T}(z,j,f) \ge Q_z\big (X_-(s) = x \;\;\forall s\in I_j) = Q_z\big (X_-({\textstyle {\frac{j+1}{n}}}) = x). \end{aligned}$$

Under $Q_z$, $X_-$ jumps at rate $2{\hat{R}}_X^-(j)T$, so we deduce that

47 $$\begin{aligned} q^X_{n,3M,T}(z,j,f) \ge \exp \Big (-\frac{2{\hat{R}}_X^-(j)T}{n}\Big ). \end{aligned}$$

On the other hand we have

$$\begin{aligned}&\int \limits _{j/n}^{(j+1)/n} \Big (\sqrt{2R^*_X(f(s))}-\sqrt{f'_X(s)}\Big )^2 ds \\&\hspace{5mm}\ge 2\int \limits _{j/n}^{(j+1)/n} R^*_X(f(s)) ds - 2\int \limits _{j/n}^{(j+1)/n} \sqrt{2R^*_X(f(s))n\big (f_X({\textstyle {\frac{j+1}{n}}})-f_X({\textstyle {\frac{j}{n}}})\big )} ds \\&\hspace{90mm}+ f_X({\textstyle {\frac{j+1}{n}}}) - f_X({\textstyle {\frac{j}{n}}}). \end{aligned}$$

By (18),

$$\begin{aligned} R_X^*(f(s)) \ge {\hat{R}}_X^+(j) - \delta _{M,T}(j,n) \ge {\hat{R}}_X^-(j) - \delta _{M,T}(j,n), \end{aligned}$$

so since $f_X({\textstyle {\frac{j+1}{n}}}) - f_X({\textstyle {\frac{j}{n}}}) \le {\textstyle {\frac{1}{n^2}}}$ and $R_X^*(f(s))\le M$ for all *s*,

$$\begin{aligned} \int \limits _{j/n}^{(j+1)/n} \Big (\sqrt{2R^*_X(f(s))}-\sqrt{f'_X(s)}\Big )^2 ds \ge \frac{2{\hat{R}}^-_X(j) - 2\delta _{M,T}(j,n)}{n} - \frac{2 \sqrt{2}M^{1/2}}{n^{3/2}}. \end{aligned}$$

The result now follows from this and (47).

**Case 2:** $$f_X(\frac{j+1}{n})> x+\frac{1}{2n^2}$$.

Note that $X_-$ jumps at rate $2{\hat{R}}_X^-(j)T$ and has exponential jumps of parameter *T* under $Q_z$. We therefore aim to apply Lemma 6.2, with $A=n(f_X(\frac{j+1}{n})-f_X(\frac{j}{n}))$, $\delta = 1/n^2$, $t=1/n$ and $a=x-f_X(j/n)$. We need to check that $a<tA/2$; to see this, note that since $z\in {\mathcal {Z}}(j)$ and we are in Case 2,

$$\begin{aligned} 2a=2\big (x-f_X({\textstyle {\frac{j}{n}}})\big )\le {\textstyle {\frac{1}{2n^2}}} + x-f_X({\textstyle {\frac{j}{n}}}) < f_X({\textstyle {\frac{j+1}{n}}}) - f_X({\textstyle {\frac{j}{n}}})=tA. \end{aligned}$$

It is also easy to check that for $T>8n^{9/2}M^{3/2}$, *T* is large enough that the conclusion of Lemma 6.2 holds. Thus applying Lemma 6.2 to (46) gives

$$\begin{aligned} q^X_{n,3M,T}(z,j,f)\ge &   \frac{1}{2} \exp \bigg (-\frac{T}{n}\Big (\sqrt{2{\hat{R}}_X^-(j)}- \sqrt{n\big (f_X({\textstyle {\frac{j+1}{n}}})-f_X({\textstyle {\frac{j}{n}}})\big )}\Big )^2 \bigg ) \\  &   \cdot \exp \bigg ( -\frac{1}{n^2}\Big (1+\sqrt{2\hat{R}_X^-(j)}\big (\sqrt{2n}+1/2\big )\Big )T\bigg ). \end{aligned}$$

Since $f\in G_M^2$, we have ${\hat{R}}_X^-(j)\le M$ and therefore

$$\begin{aligned} 1+\sqrt{2{\hat{R}}_X^-(j)}\big (\sqrt{2n}+1/2\big ) \le 1 + \sqrt{2M}\big (\sqrt{2n}+1/2\big ) \le 2(M+1)n^{1/2}. \end{aligned}$$

Thus

48 $$\begin{aligned} q^X_{n,3M,T}(z,j,f) \ge \frac{1}{2} \exp \bigg (-\frac{T}{n}\Big (\sqrt{2{\hat{R}}_X^-(j)} - \sqrt{n\big (f_X({\textstyle {\frac{j+1}{n}}})-f_X({\textstyle {\frac{j}{n}}})\big )}\Big )^2 - \frac{2(M+1)T}{n^{3/2}}\bigg ).\nonumber \\ \end{aligned}$$

Noting that since $f\in {{\,\textrm{PL}\,}}_n^2$ we have $n\big (f_X({\textstyle {\frac{j+1}{n}}})-f_X({\textstyle {\frac{j}{n}}})\big ) = f'(s)$ for all $s\in I_j$, and by (18)

$$\begin{aligned} \big (R^*_X(f(s))-\delta _{M,T}(j,n)\big )\vee 0 \le {\hat{R}}_X^-(j) \le {\hat{R}}_X^+(j) \le R_X^*(f(s))+\delta _{M,T}(j,n), \end{aligned}$$

we deduce that

$$\begin{aligned} \frac{2}{n}{\hat{R}}_X^-(j) \le \int \limits _{j/n}^{(j+1)/n} 2R^*_X(f(s))ds + \frac{2\delta _{M,T}(j,n)}{n} \end{aligned}$$

and using also that $\sqrt{(a-b)\wedge 0} \ge \sqrt{a} - \sqrt{b}$ for $a,b\ge 0$,

$$\begin{aligned}  &   \frac{1}{n}\sqrt{2{\hat{R}}_X^-(j)n\big (f_X({\textstyle {\frac{j+1}{n}}})-f_X({\textstyle {\frac{j}{n}}})\big )}\\  &   \ge \int \limits _{j/n}^{(j+1)/n} \sqrt{2R^*_X(f(s))f'_X(s)}ds - \frac{1}{\sqrt{n}}\sqrt{2\delta _{M,T}(j,n)\big (f_X({\textstyle {\frac{j+1}{n}}})-f_X({\textstyle {\frac{j}{n}}})\big )}. \end{aligned}$$

Thus

$$\begin{aligned}  &   \frac{1}{n}\Big (\sqrt{2{\hat{R}}_X^-(j)} - \sqrt{n\big (f_X({\textstyle {\frac{j+1}{n}}})-f_X({\textstyle {\frac{j}{n}}})\big )}\Big )^2 \\  &   \le \int \limits _{j/n}^{(j+1)/n} \Big (\sqrt{2R^*_X(f(s))} - \sqrt{f'(s)}\Big )^2 ds + \frac{2\delta _{M,T}(j,n)}{n} \\  &   \qquad \qquad + \frac{1}{\sqrt{n}}\sqrt{2\delta _{M,T}(j,n)\big (f_X({\textstyle {\frac{j+1}{n}}})-f_X({\textstyle {\frac{j}{n}}})\big )}. \end{aligned}$$

This combines with (48) to give the result.

## The final details for the upper bound

### Compactness and semicontinuity

There are a few more technical issues that must be resolved in order to complete the proof of the upper bound in Theorem 1.1. One of the remaining ingredients is to prove that the set of functions that we are interested in can be covered by a finite collection of small balls around suitably chosen functions. Recall that ${{\,\textrm{PL}\,}}_n$ is the subset of functions in *E* that are linear on each interval $[i/n,(i+1)/n]$ for all $i=0,\ldots ,n-1$ and continuous on [0, 1]. For $F\subset E$ and $r>0$, write $B_d(F,r) = \bigcup _{f\in F} B_d(f,r)$, where $B_d(f,r)$ is the ball of radius *r* about *f* in the metric *d*.

#### Lemma 7.1

Suppose that $F\subset E^2$ and $M>1$. For any $n\ge 4M$, there exist $N\in \mathbb {N}\cup \{0\}$ and $g_1,\ldots ,g_N\in G_{4\,M}^2\cap {{\,\textrm{PL}\,}}_n^2$ such that

$$\begin{aligned} F\cap G_{M,T}^2\subset \bigcup _{i=1}^N \big ( B_{\Delta _n}(g_i,1/n^2) \cap B_d(g_i,1/n)\big )\subset B_d(F,2/n) \end{aligned}$$

for all $$T\ge (4Mn)^{3/2}$$.

We will prove this in Appendix C.1.

In order to check that the supremum of our rate function *K* over $f\in B_d(F,\varepsilon )$ is close to the supremum over $f\in F$ when $\varepsilon$ is small, we will need to show that *K* has some form of upper semi-continuity.

#### Lemma 7.2

Suppose that $0<\theta \le 1$ and there exists $M\in (1,\infty )$ such that $f,f_n\in G_M^2$ for all *n*. Suppose also that either *f* is continuous at $\theta$, or $\theta =1$. If $d(f_n,f)\rightarrow 0$ then

$$\begin{aligned} \limsup _{n\rightarrow \infty } K(f_n,0,\theta ) \le K(f,0,\theta ). \end{aligned}$$

This will be used in the proofs of Proposition 2.10 and Lemma 7.3. We will prove it in Appendix C.3.

### The result for fixed *T*: proof of Propositions 2.6 and 2.7

#### Proof of Proposition 2.6

By Markov’s inequality, for any $\kappa >0$,

$$\begin{aligned} \mathbb {P}\Big (N_T\big (\Gamma _{M,T}(g,n),\theta \big ) \ge \kappa \Big )\le \mathbb {E}\bigg [\sum _{v\in \mathcal {N}_T} \mathbbm {1}_{\{Z_v^T|_{[0,\theta ]}\in \Gamma _{M,T}(g,n)|_{[0,\theta ]}\}}\bigg ]\frac{1}{\kappa }, \end{aligned}$$

and by Lemma 2.2,

$$\begin{aligned} \mathbb {E}\bigg [\sum _{v\in \mathcal {N}_T} \mathbbm {1}_{\{Z_v^T|_{[0,\theta ]}\in \Gamma _{M,T}(g,n)|_{[0,\theta ]}\}}\bigg ] = \mathbb {Q}\left[ \mathbbm {1}_{\{\xi ^T|_{[0,\theta ]}\in \Gamma _{M,T}(g,n)|_{[0,\theta ]}\}}e^{\int \limits _0^{\theta T} R(\xi _s)ds}\right] . \end{aligned}$$

Now, if $\xi ^T|_{[0,\theta ]}\in \Gamma _{M,T}(g,n)|_{[0,\theta ]}$, then by Lemma 2.5,

$$\begin{aligned} \int \limits _0^{\theta T} R(\xi _s)ds = T\int \limits _0^\theta R(T\xi ^T(s)) ds \le T\int \limits _0^{\lfloor \theta n\rfloor /n} R^*(g(s))ds + T\eta (M,n,T), \end{aligned}$$

and therefore

$$\begin{aligned}  &   \mathbb {Q}\left[ \mathbbm {1}_{\{\xi ^T|_{[0,\theta ]}\in \Gamma _{M,T}(g,n)|_{[0,\theta ]}\}}e^{\int \limits _0^{\theta T} R(\xi _s)ds}\right] \\  &   \le \mathbb {Q}\big (\xi ^T|_{[0,\theta ]}\in \Gamma _{M,T}(g,n)|_{[0,\theta ]}\big ) e^{T\int \limits _0^{\lfloor \theta n\rfloor /n} R^*(g(s))ds + T\eta (M,n,T)}. \end{aligned}$$

We also know from Proposition 2.3 that

$$\begin{aligned}  &   \mathbb {Q}(\xi ^T|_{[0,\theta ]}\in \Gamma _{M,T}(g,n)|_{[0,\theta ]}) \\  &   \le \exp \bigg (-T\sum _{j=0}^{\lfloor \theta n\rfloor -1} \big (\mathcal E^+_X(I_j,\Gamma _{M,T}(g,n),T) + \mathcal E^+_Y(I_j,\Gamma _{M,T}(g,n),T)\big )\bigg ), \end{aligned}$$

and by Proposition 2.4 that, if $g = (g_X,g_Y)$,

$$\begin{aligned}  &   \sum _{j=\lceil \sqrt{n}\rceil }^{\lfloor \theta n\rfloor -1} \mathcal E^+_X(I_j,\Gamma _{M,T}(g,n),T) \\  &   \ge \int \limits _{\lceil \sqrt{n}\rceil /n}^{\lfloor \theta n\rfloor /n} \Big (\sqrt{2R_X^*(g(s))} -\sqrt{g_X'(s)}\Big )^2 ds - O\Big (\frac{M^4}{n^{1/4}} +\frac{M^3n}{T^{1/2}}\Big ). \end{aligned}$$

Since $g\in G_{M}^2$, we also have

49 $$\begin{aligned} \int \limits _0^{\lceil \sqrt{n}\rceil /n} \Big (\sqrt{2R_X^*(g(s))} - \sqrt{g'_{X}(s)}\Big )^2 ds&\le \int \limits _0^{\lceil \sqrt{n}\rceil /n} 2R_X^*(g(s)) ds + \int \limits _0^{\lceil \sqrt{n}\rceil /n} g'_{X}(s) ds\nonumber \\&\le \frac{2M^2\lceil \sqrt{n}\rceil }{n} + \frac{M\lceil \sqrt{n}\rceil }{n} \le \frac{4M^2}{\sqrt{n}} \end{aligned}$$

so

$$\begin{aligned}  &   \sum _{j=\lceil \sqrt{n}\rceil }^{\lfloor \theta n\rfloor -1} \mathcal E^+_X(I_j,\Gamma _{M,T}(g,n),T) \\  &   \ge \int \limits _0^{\lfloor \theta n\rfloor /n} \Big (\sqrt{2R_X^*(g(s))} - \sqrt{g_X'(s)}\Big )^2 ds - O\Big (\frac{M^4}{n^{1/4}} + \frac{M^3n}{T^{1/2}}\Big ) \end{aligned}$$

and by symmetry the same bound holds for *Y*. Recalling from Lemma 2.5 that $\eta (M,n,T) = O\Big (\frac{M^4}{n^{1/2}} + \frac{M^3n}{T^{1/3}}\Big )$, we deduce that

$$\begin{aligned}&\mathbb {P}\Big (N_T\big (\Gamma _{M,T}(g,n),\theta \big ) \ge \kappa \Big )\\&\quad \le e^{-T\int \limits _0^{\lfloor \theta n\rfloor /n} \big (\sqrt{2R_X^*(g(s))} - \sqrt{g_X'(s)}\big )^2 ds - T\int \limits _0^{\lfloor \theta n\rfloor /n} \big (\sqrt{2R_Y^*(g(s))} - \sqrt{g_Y'(s)}\big )^2 ds}\\&\quad \cdot e^{O\left( \frac{M^4T}{n^{1/4}} + M^3nT^{2/3}\right) + T\int \limits _0^{\lfloor \theta n\rfloor /n} R^*(g(s))ds}\cdot \frac{1}{\kappa }\\&\quad = \frac{1}{\kappa } \exp \bigg ( T K\Big (g,0,\tfrac{\lfloor \theta n\rfloor }{n}\Big ) + O\Big (\tfrac{M^4T}{n^{1/4}} + M^3n T^{2/3}\Big )\bigg ) \end{aligned}$$

as required, where for the last equality we used the fact that $g\in G_M^2\cap {{\,\textrm{PL}\,}}_n^2$, and therefore $K(g,0,s)=\int _0^s R^*(g(u))du - I(g,0,s)$ for all *s*.$\square$

Proposition 2.7 essentially establishes the upper bound in Theorem 1.1 with high probability for a fixed (large) *T*. The proof mostly involves using Lemma 2.1 and the technical results stated in Sect. 7.1 to ensure that we can cover our set in a suitable way with finitely many balls around piecewise linear functions, and then applying Proposition 2.6.

#### Proof of Proposition 2.7

Take $M\ge M_0$ and the other parameters as in the statement of the Proposition. By Lemma 2.1,

$$\begin{aligned} \mathbb {P}(\exists v\in \mathcal {N}_T: Z^T_v\not \in G_{M,T}^2) \le e^{-\delta _0 T^{1/3}}. \end{aligned}$$

By Proposition 2.10, since *F* is closed we may choose *n* large enough such that $n\ge 4M$ and

$$\begin{aligned} \sup _{f\in B_d(F,2/n)\cap G_{4M}^2} K(f,0,1) \le \sup _{f\in F\cap G_{4M}^2} K(f,0,1) + \varepsilon /3. \end{aligned}$$

By Lemma 7.1 we may choose $N\in \mathbb {N}$ and $g_1,\ldots ,g_N\in G_{4M}^2\cap {{\,\textrm{PL}\,}}_n^2$ such that

$$\begin{aligned} F\cap G_{M,T}^2 \subset \bigcup _{i=1}^N \big (B_{\Delta _n}(g_i,1/n^2)\cap B_d(g_i,1/n)\big ) \subset B_d(F,2/n) \end{aligned}$$

for all $T\ge (4Mn)^{3/2}$. Recall that $\Gamma _{M,T}(g_i,n) = B_{\Delta _n}(g_i,1/n^2)\cap B_d(g_i,1/n)\cap G_{M,T}^2$. Then for any $A\ge 0$,

50 $$\begin{aligned} \mathbb {P}\big (N_T(F) \ge e^{A T}\big )&\le \mathbb {P}\big (\exists v\in \mathcal {N}_T : Z_v^T \not \in G_{M,T}^2\big ) + \sum _{i=1}^N \mathbb {P}\bigg (N_T\big (\Gamma _{M,T}(g_i,n)\big ) \ge \frac{e^{A T}}{N}\bigg )\nonumber \\&\le e^{-\delta _0 T^{1/3}} + \sum _{i=1}^N \mathbb {P}\bigg (N_T\big (\Gamma _{M,T}(g_i,n)\big ) \ge \frac{e^{A T}}{N}\bigg ). \end{aligned}$$

By Proposition 2.6, for each *i* we have

$$\begin{aligned} \mathbb {P}\bigg (N_T\big (\Gamma _{M,T}(g_i,n)\big ) \ge \frac{e^{A T}}{N}\bigg ) \le \frac{N}{e^{AT}}\exp \bigg (T K(g_i,0,1) + O\Big (\frac{M^4T}{n^{1/4}} + M^3n T^{2/3}\Big )\bigg ), \end{aligned}$$

and combining this with (50) we see that

$$\begin{aligned}  &   \mathbb {P}\big (N_T(F) \ge e^{A T}\big ) \\  &   \le e^{-\delta _0 T^{1/3}} + \frac{N^2}{e^{A T}}\max _{i\in \{1,\ldots ,N\}} \exp \bigg (T K(g_i,0,1) + O\Big (\frac{M^4T}{n^{1/4}} + M^3n T^{2/3}\Big )\bigg ). \end{aligned}$$

By our choice of $g_1,\ldots ,g_N$ and *n*, we have

$$\begin{aligned} \max _{i\in \{1,\ldots ,N\}} K(g_i,0,1) \le \sup _{f\in B_d(F,2/n)\cap G^2_{4M}}K(f,0,1) \le \sup _{f\in F\cap G^2_{4M}} K(f,0,1) + \varepsilon /3 \end{aligned}$$

and therefore

$$\begin{aligned}  &   \frac{1}{T^{1/3}}\log \mathbb {P}\big (N_T(F) \ge e^{A T}\big ) \\  &   \le (-\delta _0)\vee \bigg (\sup _{f\in F\cap G^2_{4M}} K(f,0,1) T^{2/3}- A T^{2/3} + \frac{\varepsilon T^{2/3}}{3} + O\Big (\frac{M^4 T^{2/3}}{n^{1/4}}\Big )\bigg ). \end{aligned}$$

Increasing *n* if necessary so that the $O(\frac{M^4 T^{2/3}}{n^{1/4}})$ term is smaller than ${\textstyle {\frac{\varepsilon T^{2/3}}{3}}}$, and choosing

$$\begin{aligned} A = \sup _{f\in F\cap G_{4M}^2} K(f,0,1) + \varepsilon , \end{aligned}$$

we have

$$\begin{aligned} \lim _{T\rightarrow \infty } \frac{1}{T^{1/3}}\log \mathbb {P}\big (N_T(F,\theta ) \ge e^{A T}\big ) \le -\delta _0. \end{aligned}$$

This is precisely the statement of the proposition, but with 4*M* in place of *M*. Since we only assumed that $M\ge M_0$ in the proof, the proposition holds when $M\ge 4M_0$.$\square$

### Paths with $K^+(f)=-\infty$ are unlikely: proof of Lemma 2.8

Before proving Lemma 2.8, we need to relate $K^+$ to *K*.

#### Lemma 7.3

Suppose that $M>1$. If $F\subset E^2$ is closed and $\sup _{f\in F} K^+(f) = -\infty$, then there exists $\varepsilon >0$ such that

$$\begin{aligned} \sup _{f\in B(F,\varepsilon )\cap G_{M,1}^2} \inf _{\theta \in [0,1]} K(f,0,\theta ) < 0. \end{aligned}$$

#### Proof

If the result is not true, then for each $n\in \mathbb {N}$ we may choose $f_n\in B(F,1/n)\cap G_{M,1}^2$ such that

$$\begin{aligned} \inf _{\theta \in [0,1]} K(f_n,0,\theta ) \ge -1/n. \end{aligned}$$

It is easy to check that $G_{M,1}^2$ is closed and totally bounded. Since $(E^2,d)$ is complete, $G_{M,1}^2$ is compact. We may therefore find a subsequence $(f_{n_j})_{j\ge 1}$ such that $d(f_{n_j},f_\infty )\rightarrow 0$ as $j\rightarrow \infty$ for some $f_\infty \in G_{M,1}^2$. Since $F\cap G_{M,1}^2$ is closed, and $d(f_\infty ,F\cap G_{M,1}^2)=0$, we must in fact have $f_\infty \in F\cap G_{M,1}^2$. On the other hand, by Lemma 7.2, for any $\theta \in [0,1]$ such that $f_\infty$ is continuous at $\theta$,

$$\begin{aligned} K(f_\infty ,0,\theta ) \ge \limsup _{j\rightarrow \infty }K(f_{n_j},0,\theta ) \ge 0. \end{aligned}$$

But $f_\infty$ is non-decreasing and therefore continuous almost everywhere, and $t\mapsto K(f,0,t)$ has only downward jumps, so we must have $K(f_\infty ,0,\theta )\ge 0$ for all $\theta \in [0,1]$. Thus $K^+(f_\infty )\ge 0$, which contradicts the hypothesis of the lemma. $\square$

We can now prove Lemma 2.8, which says that if *F* is closed and $\sup _{f\in F} K^+(f) = -\infty$, then with high probability $N_T(F)$ is zero.

#### Proof of Lemma 2.8

Choose $M\ge M_0$. Since $G_{4M}\subset G_{4M,1}$, by Lemma 7.3 we may choose $n_0\ge 4M$ such that

$$\begin{aligned} \sup _{f\in B(F,2/n_0)\cap G_{4M}^2} \inf _{\theta \in [0,1]} K(f,0,\theta )<0. \end{aligned}$$

Let

51 $$\begin{aligned} \eta = -\sup _{f\in B(F,2/n_0)\cap G_{4M}^2} \inf _{\theta \in [0,1]} K(f,0,\theta ) > 0. \end{aligned}$$

Then take $n\ge n_0$ such that the error term in Proposition 2.6 is smaller than $\eta T/3$ for *T* sufficiently large, and such that $(4M)^2/n \le \eta /3$.

By Lemma 7.1 we may choose $N\in \mathbb {N}\cup \{0\}$ and $g_1,\ldots ,g_N\in G_{4M}^2\cap {{\,\textrm{PL}\,}}_n^2$ such that

$$\begin{aligned} F\cap G_{M,T}^2 \subset \bigcup _{i=1}^N \big ( B_{\Delta _n}(g_i,1/n^2) \cap B_d(g_i,1/n)\big )\subset B_d(F,2/n) \end{aligned}$$

for all $$T\ge (4Mn)^{3/2}$$.

For each $i=1,\ldots ,N$, note that since $g_i\in G_{4M}^2$, by the definition of *K*, for any $0\le s\le t\le 1$ we have

52 $$\begin{aligned} K(g_i,0,t) \le K(g_i,0,s) + (4M)^2(t-s). \end{aligned}$$

In particular, the function $t\mapsto K(g_i,0,t)$ has only downward jumps, and therefore its infimum is achieved. Thus, by (51), we may choose $\theta _i$ such that

$$\begin{aligned} K(g_i,0,\theta _i) = \inf _{\theta \in [0,1]} K(g_i,0,\theta )\le -\eta . \end{aligned}$$

Let $\hat{\theta }_i = \lceil \theta _i n\rceil /n$. Using (52) again, we then have

53 $$\begin{aligned} K(g_i,0,\hat{\theta }_i) \le -\eta + (4M)^2/n \le -2\eta /3 \end{aligned}$$

where the last inequality holds because we chose *n* such that $(4M)^2/n\le \eta /3$.

Now, by our choice of $g_1,\ldots ,g_N$, we have

$$\begin{aligned} N_T(F) \le N_T((G_{M,T}^2)^c) + \sum _{i=1}^N N_T(\Gamma _{M,T}(g_i,n)) \end{aligned}$$

and therefore

54 $$\begin{aligned} \mathbb {P}(N_T(F)\ge 1) \le \mathbb {P}\big (N_T((G_{M,T}^2)^c)\ge 1\big ) + \sum _{i=1}^N \mathbb {P}\big (N_T(\Gamma _{M,T}(g_i,n))\ge 1\big ). \end{aligned}$$

By Lemma 2.1, the first term on the right-hand side above is at most $e^{-\delta _0 T^{1/3}}$. Also, since a population that is extinct at time $\theta$ must also be extinct at time 1, for each *i* we have

$$\begin{aligned} \mathbb {P}\big (N_T(\Gamma _{M,T}(g_i,n))\ge 1\big ) \le \mathbb {P}\big (N_T(\Gamma _{M,T}(g_i,n),\hat{\theta }_i)\ge 1\big ). \end{aligned}$$

Since $\hat{\theta }_i$ is an integer multiple of 1/*n*, by Proposition 2.6 we have

$$\begin{aligned} \mathbb {P}\big (N_T(\Gamma _{M,T}(g_i,n),\hat{\theta }_i)\ge 1\big )&\le \exp \bigg (T K(g,0,\hat{\theta }_i) + O\Big (\frac{M^4T}{n^{1/4}} + M^3 n T^{2/3}\Big )\bigg )\\&\le \exp \Big (\! -\frac{\eta T}{3}\Big ), \end{aligned}$$

where the last inequality follows from (53) and our choice of *n*. Returning to (54), we have shown that

$$\begin{aligned} \mathbb {P}(N_T(F)\ge 1) \le e^{-\delta _0 T^{1/3}} + N e^{-\eta T/3}, \end{aligned}$$

which completes the proof.$\square$

### Lattice times to continuous time: proof of Proposition 2.9

Before moving on to the proof of Proposition 2.9, we state and prove two lemmas that will check that paths of particles are not drastically changed by rescaling by a slightly different value of *T*.

#### Lemma 7.4

Suppose that $M>1$, $t\ge 3\,M$ and $t-1\le s\le t$. For any $F\subset E^2$, we have

$$\begin{aligned} N_s(F\cap G_{M,s}^2)\le N_t\big (B(F, 3M/t)\big ). \end{aligned}$$

#### Proof

Suppose that $u\in \mathcal {N}_{s}$ satisfies $Z_u^s\in F\cap G_{M,s}^2$. Take any $v\in \mathcal {N}_{t}$ such that *v* is a descendant of *u*. We claim that $d(X_u^s, X_v^t)\le 3M/t$, which means that for all $\tau \in [-3M/t,1+3M/t]$,

$$\begin{aligned} X_v^{t}(\tau -3M/t)-3M/t \le X_u^s(\tau ) \le X_v^{t}(\tau +3M/t) + 3M/t \end{aligned}$$

where $f(\tau )$ is interpreted to equal *f*(0) for $\tau <0$ and *f*(1) for $\tau >1$. Since $Z_u^s\in F$, the claim plus its equivalent *Y* statement ensure that $Z_v^t\in B(F, 3M/t)$, which is enough to complete the proof.

To prove the claim, first note that it holds when $\tau \le 0$, since in this case $X_u^s(\tau )=X_u^s(0)=X_v^t(0) = X_v^t(\tau )$. If $\tau >0$, since $s\le t$ and

$$\begin{aligned} \tau s \ge \tau (t-1) = t(\tau - \tau /t) \ge t\Big (\tau -\frac{1+3M/t}{t}\Big ) \ge t\Big (\tau - \frac{3M}{t}\Big ), \end{aligned}$$

we have

$$\begin{aligned} X^s_u(\tau ) = X^s_v(\tau ) \ge X_v^t(\tau - {\textstyle {\frac{3M}{t}}}). \end{aligned}$$

Also, since $X_u^s\in G_{M,s}^2$, for any $\tau \in [0,1]$ we have

$$\begin{aligned} X^s_u(\tau ) = \frac{1}{s}X_u(\tau s)&= \frac{1}{t}X_u(\tau s) + \Big (1-\frac{s}{t}\Big )X_u^s(\tau ) \\&\le \frac{1}{t}X_v(\tau t) + \Big (\frac{t-s}{t}\Big ) M(1+2s^{-2/3}) \\&\le X_v^{t}(\tau ) + \frac{M}{t}(1+2s^{-2/3}) \le X_v^{t}(\tau ) + \frac{3M}{t} \end{aligned}$$

as required. If $\tau >1$ then $X^s_u(\tau ) = X^s_u(1)$ and then the argument above gives that $X^s_u(1)\le X^t_v(1) + 3\,M/t = X^t_v(\tau ) + 3\,M/t$. $\square$

#### Lemma 7.5

Suppose that $M>2$, $T\ge 2$ and $t\in [T-1,T]$. If $N_t((G_{M,t}^2)^c)\ge 1$ then either $N_T((G_{M/2,T}^2)^c)\ge 1$ or $N_{T-1}((G_{M/2,T-1}^2)^c)\ge 1$.

#### Proof

Suppose there exists $v\in \mathcal {N}_{t}$ such that $Z^t_v\in (G_{M,t}^2)^c$. It is possible that either $X^t_v$ or $Y^t_v$ (or both) is the reason for $Z^t_v$ falling outside $G_{M,t}^2$; without loss of generality assume that it is $X^t_v$. Then there exists $s\in [0,1]$ such that either $X^t_v(s) > M(s+2t^{-2/3})$, or $X^t_v(s)<s/M - 2t^{-2/3}$. In the first case, take $w\in \mathcal {N}_{T}$ such that *w* is a descendant of *v*. Then

$$\begin{aligned} X^T_w(s) = \frac{1}{T}X_w(sT) \ge \frac{t}{T}\frac{1}{t}X_v(st) > \frac{1}{2}M(s+2t^{-2/3}) \ge \frac{M}{2}(s+2T^{-2/3}) \end{aligned}$$

so $Z^T_w\in (G_{M/2,T}^2)^c$. In the second case, let *u* be the ancestor of *v* in $\mathcal {N}_{T-1}$. Then

$$\begin{aligned} X^{T-1}_u(s) = \frac{1}{T-1}X_u(s(T-1)) \le \frac{t}{T-1}\frac{1}{t}X_v(st)&< \frac{t}{T-1}\Big (\frac{s}{M}-2t^{-2/3}\Big ) \\&\le \frac{2s}{M} - 2(T-1)^{-2/3} \end{aligned}$$

so $Z^{T-1}_u \in (G_{M/2,T-1}^2)^c$. This completes the proof. $\square$

#### Proof of Proposition 2.9

We begin with the first part of the result. Take $\varepsilon >0$. We start by noting that

55 $$\begin{aligned}  &   \mathbb {P}\Big (\exists t\in [T-1,T]: \frac{1}{t}\log N_t(F)\ge \sup _{f\in F\cap G_M^2} K(f,0,1) + \varepsilon \Big )\nonumber \\  &   \le \mathbb {P}\Big (\exists t\in [T-1,T]: \frac{1}{t}\log N_t(F\cap G_{M,t}^2)\ge \sup _{f\in F\cap G_M^2} K(f,0,1) + \varepsilon \Big )\nonumber \\  &   + \mathbb {P}\big (\exists t\in [T-1,T]: N_t((G_{M,t}^2)^c)\ge 1\big ). \end{aligned}$$

We show that the right-hand side is exponentially small in *T*. By Proposition 2.10, we can choose $\varepsilon '\in (0,1)$ such that

$$\begin{aligned} \sup _{f\in \overline{B(F,\varepsilon ')}\cap G_M^2} K(f,0,1) \le \sup _{f\in F\cap G_M^2} K(f,0,1) + \varepsilon /3. \end{aligned}$$

By Lemma 7.4, provided that $3M/T\le \varepsilon '$, we have

$$\begin{aligned} N_t(F\cap G_{M,t}^2) \le N_T(B(F,\varepsilon ')) \end{aligned}$$

for all $t\in [T-1,T]$. Therefore for large *T*

$$\begin{aligned}&\mathbb {P}\Big (\exists t\in [T-1,T] : \frac{1}{t}\log N_t(F\cap G_{M,t}^2)\ge \sup _{f\in F\cap G_M^2} K(f,0,1) + \varepsilon \Big )\\&\quad \le \mathbb {P}\Big (\frac{1}{T-1}\log N_T(B(F,\varepsilon '))\ge \sup _{f\in F\cap G_M^2} K(f,0,1) + \varepsilon \Big )\\&\quad \le \mathbb {P}\Big (\frac{1}{T}\log N_T(B(F,\varepsilon '))\ge \sup _{f\in \overline{B(F,\varepsilon ')}\cap G_M^2} K(f,0,1) + \varepsilon /3 \Big ). \end{aligned}$$

Then Proposition 2.7 tells us that this is at most $\exp (-\delta _0 T^{1/3}/2)$ for large *T*. Substituting this into (55), we have

56 $$\begin{aligned}  &   \mathbb {P}\Big (\exists t\in [T-1,T]: \frac{1}{t}\log N_t(F)\ge \sup _{f\in F\cap G_M^2} K(f,0,1) + \varepsilon \Big )\nonumber \\  &   \le \exp (-\delta _0 T^{1/3}/2) + \mathbb {P}\big (\exists t\in [T-1,T]: N_t((G_{M,t}^2)^c)\ge 1\big ). \end{aligned}$$

For the remaining term, Lemma 7.5 tells us that for $T\ge 2$,

$$\begin{aligned}&\mathbb {P}(\exists t\in [T-1,T] : N_t((G_{M,t}^2)^c)\ge 1) \\&\quad \le \mathbb {P}(N_T((G_{M/2,T}^2)^c)\ge 1) + \mathbb {P}(N_{T-1}((G_{M/2,T-1}^2)^c)\ge 1). \end{aligned}$$

By Lemma 2.1, this is at most $2 \exp \big ( \! -\delta _0 (T-1)^{1/3} \big )$. Returning to (56), we have

$$\begin{aligned}&\mathbb {P}\Big (\exists t\in [T-1,T] : \frac{1}{t}\log N_t(F)\ge \sup _{f\in F\cap G_M^2} K(f,0,1) + \varepsilon \Big ) \\&\quad \le \exp (- \delta _0 T^{1/3}/2) + 2 \exp \big ( \! -\delta _0 (T-1)^{1/3} \big ). \end{aligned}$$

By the Borel–Cantelli lemma,

$$\begin{aligned} \mathbb {P}\Big (\limsup _{t\rightarrow \infty }\frac{1}{t}\log N_t(F)\ge \sup _{f\in F\cap G_M^2} K(f,0,1) + \varepsilon \Big ) = 0, \end{aligned}$$

and since $\varepsilon >0$ was arbitrary, we deduce the first part of the result.

The proof when $\sup _{f\in F} K^+(f) = -\infty$ is similar. By Lemma 7.3, we may choose $\varepsilon '' >0$ such that

57 $$\begin{aligned} \sup _{f\in \overline{B(F,\varepsilon '')}\cap G_{M,1}^2} K^+(f) = -\infty . \end{aligned}$$

Then

58 $$\begin{aligned} \mathbb {P}(\exists t\in [T-1,T]: N_t(F) \ge 1 )\le &   \mathbb {P}(\exists t\in [T-1,T]: N_t(F\cap G_{M,t}^2) \ge 1 )\nonumber \\  &   + \mathbb {P}(\exists t\in [T-1,T]: N_t((G_{M,t}^2)^c) \ge 1 ).\nonumber \\ \end{aligned}$$

As argued above, by Lemmas 7.5 and 2.1 the last term on the right-hand side is at most $2 \exp \big ( \! -\delta _0 (T-1)^{1/3} \big )$ provided that $T\ge 2$. For the first term on the right-hand side, by Lemma 7.4, provided that $3M/T\le \varepsilon ''$ we have

$$\begin{aligned}&\mathbb {P}(\exists t\in [T-1,T] : N_t(F\cap G_{M,t}^2) \ge 1 )\\&\le \mathbb {P}(N_T( \overline{B(F,\varepsilon '')}) \ge 1) \le \mathbb {P}(N_T(\overline{B(F,\varepsilon '')}\cap G_{M,1}^2)\ge 1) + \mathbb {P}(N_T((G_{M,1}^2)^c)\ge 1). \end{aligned}$$

Due to (57), we can apply Lemma 2.8 to tell us that the first term on the right-hand side above is at most $\exp ( \! -\delta _0 T^{1/3}/2)$, and Lemma 2.1 to tell us that the second term on the right-hand side is at most $\exp ( \! -\delta _0 T^{1/3})$. Returning to (58), and applying the Borel-Cantelli lemma, we have

$$\begin{aligned} \mathbb {P}\big (\limsup _{t\rightarrow \infty } N_t(F) \ge 1 \big ) = 0. \end{aligned}$$

This completes the proof.

## Data Availability

No data were created during the study.

## Deterministic bounds on the rate function

We use the same notation as in Sect. 6. Our main aim in this section is to prove Proposition 2.4 and Lemma 2.5, showing that the bounds obtained in Sect. 6, in terms of $\mathcal E^+_X(I_j,\Gamma _{M,T}(f,n),T)$, look something like the growth rate seen in our main theorem. This work involves tedious approximations of sums and integrals. Most of the work is in bounding $R^*$ in terms of $R^+$ and $R^-$, which is done using the following lemma. Throughout this section we write $R^-_X(j) = R^-_X(I_j,\Gamma _{M,T}(f,n),T)$ and similarly for $R^+_X$, $R^-_Y$ and $R^+_Y$.

### Lemma A.1

Suppose that $M>1$, $n\ge 2M$, $f|_{I_j}\in G_M^2|_{I_j}$, $j\ge n^{1/2}$ and $s\in I_j$. Then

59 $$\begin{aligned} R_X^+(j) - \delta _{M,T}(j,n) \le R_X^*(f(s)) \le R_X^-(j) + \delta _{M,T}(j,n) \end{aligned}$$

where

$$\begin{aligned} \delta _{M,T}(j,n)= &   \big (6M^3n^{1/2}+{\textstyle {\frac{2M^2n}{T}}}\big )\big (f_X({\textstyle {\frac{j+1}{n}}})-f_X({\textstyle {\frac{j}{n}}})\big )\\  &   \quad + Mn^{1/2}\big (f_Y({\textstyle {\frac{j+1}{n}}})-f_Y({\textstyle {\frac{j}{n}}})\big ) + {\textstyle {\frac{7M^3}{n^{3/2}}}} + {\textstyle {\frac{3M^3n}{T}}}. \end{aligned}$$

Moreover,

60 $$\begin{aligned} \sum _{j=\lceil \sqrt{n}\rceil }^{n-1}\frac{\delta _{M,T}(j,n)}{n} \le \frac{14M^4}{n^{1/2}}+\frac{5M^3 n}{T}. \end{aligned}$$

### Proof

We begin with the upper bound in (59), and claim first that for any $j\in \{0,1,\ldots ,n-1\}$ we have

61 $$\begin{aligned} f_Y\Big (\frac{j}{n}\Big ) \le \Big (R_X^-(j) + \frac{1}{2}\Big )\Big (f_X\Big (\frac{j+1}{n}\Big )+\frac{1}{n^2}+\frac{1}{T}\Big ) + \frac{1}{n^2}. \end{aligned}$$

To see why this is true, by the definition of $R^-_X(j)$, for any $\varepsilon >0$ we may take $g\in \Gamma _{M,T}(f,n)$ and $s\in I_j$ such that

$$\begin{aligned} R_X(Tg(s))\le R^-_X(j)+\varepsilon , \end{aligned}$$

and then

$$\begin{aligned} \frac{g_Y(s)+1/T}{g_X(s)+1/T} - \frac{1}{2} \le R_X(Tg(s)) \le R_X^-(j) + \varepsilon . \end{aligned}$$

Noting that $g_Y(s)\ge g_Y(\frac{j}{n})\ge f_Y(\frac{j}{n})-1/n^2$ and $g_X(s)\le g_X(\frac{j+1}{n})\le f_X(\frac{j+1}{n})+1/n^2$, we see that

$$\begin{aligned} \frac{f_Y(\frac{j}{n})-1/n^2 + 1/T}{f_X(\frac{j+1}{n})+1/n^2+1/T} - \frac{1}{2} \le R_X^-(j) + \varepsilon . \end{aligned}$$

Since $\varepsilon >0$ was arbitrary, the left-hand side must in fact be at most $R_X^-(j)$, and then rearranging gives (61).

We now aim to bound $R_X^*(f(s))$ above for $s\in I_j$. We concentrate first on the case that $f_Y(s)>f_X(s)$. Whenever this holds, using (61),

$$\begin{aligned} R_X^*(f(s))&= \frac{f_Y(s)}{f_X(s)}-\frac{1}{2} \\  &= \frac{f_Y(\frac{j}{n})}{f_X(s)}-\frac{1}{2} + \frac{f_Y(s)-f_Y(\frac{j}{n})}{f_X(s)}\\&\le \frac{\big (R_X^-(j)+\tfrac{1}{2}\big )\big (f_X(\frac{j+1}{n})+\tfrac{1}{n^2}+\tfrac{1}{T} \big ) + \frac{1}{n^2}}{f_X(s)}-\frac{1}{2} + \frac{f_Y(s)-f_Y(\frac{j}{n})}{f_X(s)}. \end{aligned}$$

Writing

$$\begin{aligned}  &   \Big (R_X^-(j)+\frac{1}{2}\Big )\Big (f_X\big ({\textstyle {\frac{j+1}{n}}}\big )+\frac{1}{n^2}+\frac{1}{T}\Big )\\  &   = \Big (R_X^-(j)+\frac{1}{2}\Big )f_X(s) + \Big (R_X^-(j)+\frac{1}{2}\Big )\Big (f_X\big ({\textstyle {\frac{j+1}{n}}}\big )-f_X(s)+\frac{1}{n^2}+\frac{1}{T}\Big ) \end{aligned}$$

and substituting this into the bound above, we have (for $f_Y(s)> f_X(s)$)

$$\begin{aligned}  &   R_X^*(f(s)) \\  &   \le R_X^-(j) + \frac{\big (R_X^-(j)+\frac{1}{2}\big )\big (f_X(\frac{j+1}{n})-f_X(s)+\frac{1}{n^2}+\frac{1}{T}\big ) + \frac{1}{n^2} + f_Y(s)-f_Y(\frac{j}{n})}{f_X(s)}. \end{aligned}$$

The first term on the right-hand side is the important one, and we now aim to bound the other terms. Since $f|_{I_j}\in G_M^2|_{I_j}$, we have $f_X(s)\ge s/M$, and since also $f\in \Gamma _{M,T}(f,n)$,

62 $$\begin{aligned} R_X^-(j)\le R_X(Tf(s)) \le \frac{Ms+1/T}{s/M}-\frac{1}{2} = M^2 + \frac{M}{sT} - \frac{1}{2}, \end{aligned}$$

so

$$\begin{aligned}  &   R_X^*(f(s)) \\  &   \le R_X^-(j) + \frac{\big (M^2 + \frac{M}{sT}\big )\big (f_X(\frac{j+1}{n})-f_X(\frac{j}{n})+\frac{1}{n^2}+\frac{1}{T}\big ) + \frac{1}{n^2} + f_Y(\frac{j+1}{n})-f_Y(\frac{j}{n})}{s/M}. \end{aligned}$$

This is true in the case $f_Y(s)>f_X(s)$, but when $f_Y(s)\le f_X(s)$ we have $R_X^*(f(s))=1/2\le R_X^-(j)$, so the inequality above trivially holds in that case too. Taking $s\ge j/n\ge n^{-1/2}$, and combining some of the terms, we obtain the upper bound in (59).

The lower bound in (59) is similar. We choose $g\in \Gamma _{M,T}(f,n)$ and $s\in [\frac{j}{n},\frac{j+1}{n}]$ such that $R_X(Tg(s))\ge R^+_X(j)-1/n^2$. If $R_X(Tg(s))=1/2$ then $R_X^+(j)\le 1/2+1/n^2$, so the lower bound on that interval is trivial; we may therefore assume that $R_X(Tg(s))>1/2$ and then we have a similar bound to (61):

63 $$\begin{aligned} f_Y\Big (\frac{j+1}{n}\Big ) \ge \Big (R_X^+(j) + \frac{1}{2}\Big )\Big (f_X\Big (\frac{j}{n}\Big )-\frac{1}{n^2}\Big ) - \frac{1}{n^2} - \frac{1}{T}. \end{aligned}$$

We then apply this essentially as in the proof of the upper bound to obtain

$$\begin{aligned}  &   R_X^*(f(s)) \\  &   \ge R_X^+(j) - \frac{\big (R_X^+(j)+\frac{1}{2}\big )\big (f_X(s)-f_X(\frac{j}{n})+\frac{1}{n^2}\big ) + \frac{1}{n^2} + \frac{1}{T} + f_Y(\frac{j+1}{n}) - f_Y(s)}{f_X(s)}. \end{aligned}$$

In place of (62) we must use the slightly more involved bound, for $j\ge \sqrt{n}$ and $n\ge 2\,M$,

$$\begin{aligned} R^+_X(j)&\le \frac{M\frac{j+1}{n} + \frac{1}{n^2} + \frac{1}{T}}{\frac{1}{M}\frac{j}{n} - \frac{1}{n^2} + \frac{1}{T}} - \frac{1}{2} \le \frac{3M\frac{j}{n} + \frac{1}{T}}{\frac{j}{2Mn}} - \frac{1}{2} = 6M^2 + \frac{2Mn}{jT} - \frac{1}{2} \\&\le 6M^2 + \frac{2M\sqrt{n}}{T} - \frac{1}{2}. \end{aligned}$$

Applying this and taking $s\ge j/n\ge n^{-1/2}$, and combining terms, gives the lower bound in (59).

To prove (60), summing over $j\ge n^{1/2}$ and telescoping gives

$$\begin{aligned} \sum _{j=\lceil \sqrt{n}\rceil }^{n-1}\frac{\delta _{M,T}(j,n)}{n} \le \Big (\frac{6M^3}{n^{1/2}}+\frac{2M^2}{T}\Big )f_X(1) + \frac{M}{n^{1/2}}f_Y(1) + \frac{7M^3}{n^{3/2}} + \frac{3M^3n}{T} \end{aligned}$$

Using that $f_X(1)\le M$ and $f_Y(1)\le M$, and combining terms, gives the result. $\square$

### Proof of Proposition 2.4

We first give a lemma which handles the cross-term that appears when multiplying out the quadratics involved in Proposition 2.4.

#### Lemma A.2

Suppose that $f\in {{\,\textrm{PL}\,}}_n^2 \cap G_M^2$, $\theta \in (0,1]$, $M>1$ and $n\ge 2M$. Then for any $k\in \{\lceil \sqrt{n}\rceil ,\ldots ,\lfloor \theta n\rfloor -1\}$,

$$\begin{aligned} \int \limits _{k/n}^{\lfloor \theta n\rfloor /n} \sqrt{R_X^*(f(s))f'_X(s)} ds \ge \sum _{j=k}^{\lfloor \theta n\rfloor -1} \sqrt{\frac{R_X^+(j)}{n}(x^+_{j+1}-x^-_j)} - \frac{8M^{5/2}}{n^{1/4}} - \frac{4M^2n^{1/2}}{T^{1/2}}. \end{aligned}$$

#### Proof

Take $s\in [\frac{j}{n},\frac{j+1}{n}]$ for some $j\in \{0,1,\ldots ,n-1\}$. For $a\in \mathbb {R}$, write $a_+$ to mean $\max \{a,0\}$. Note that since $f\in {{\,\textrm{PL}\,}}_n$, we have

$$\begin{aligned} f'_X(s) = n\big (f\big ({\textstyle {\frac{j+1}{n}}}\big )-f\big ({\textstyle {\frac{j}{n}}}\big )\big ) \ge n\big (x_{j+1}^+-1/n^2 - x_j^--1/n^2\big )_+.\end{aligned}$$

Using the elementary inequality $\sqrt{(a-b)_+} \ge a^{1/2}-b^{1/2}$ valid for all $a,b\ge 0$, we obtain

$$\begin{aligned}\sqrt{f'_X(s)} \ge n^{1/2}(x_{j+1}^+ - x_j^-)^{1/2} - \sqrt{2} n^{-1/2}.\end{aligned}$$

Thus

$$\begin{aligned}\int \limits _{j/n}^{(j+1)/n} \sqrt{R_X^*(f(s))f'_X(s)} ds \ge \big ((x_{j+1}^+ - x_j^-)^{1/2} - \sqrt{2}/n\big )\int \limits _{j/n}^{(j+1)/n} \sqrt{nR_X^*(f(s))} ds\end{aligned}$$

and since $f\in G_M^2$, $R_X^*(f(s))\le M^2$ for all $s>0$, so

$$\begin{aligned}\int \limits _{j/n}^{(j+1)/n} \! \! \sqrt{R_X^*(f(s))f'_X(s)} ds \ge (x_{j+1}^+ - x_j^-)^{1/2}\int \limits _{j/n}^{(j+1)/n} \! \! \sqrt{nR_X^*(f(s))} ds - \sqrt{2} Mn^{-3/2}.\end{aligned}$$

We now use the lower bound in (59) to see that for $j\ge \sqrt{n}$,

$$\begin{aligned} \int \limits _{j/n}^{(j+1)/n} \hspace{-1mm} \sqrt{nR_X^*(f(s))} ds&\ge \int \limits _{j/n}^{(j+1)/n}\hspace{-1mm} \sqrt{(nR_X^+(j)-n\delta _{M,T}(j,n))_+} \,ds \\&= \sqrt{\Big (\frac{R_X^+(j)}{n}-\frac{\delta _{M,T}(j,n)}{n}\Big )_+}. \end{aligned}$$

Again using $\sqrt{(a-b)_+} \ge a^{1/2}-b^{1/2}$, we therefore have, for $j\ge \sqrt{n}$,

64 $$\begin{aligned}  &   \int \limits _{j/n}^{(j+1)/n} \sqrt{R_X^*(f(s))f'_X(s)} ds\nonumber \\  &   \ge \sqrt{\frac{R_X^+(j)}{n}\big (x_{j+1}^+ - x_j^-\big )} - \frac{\sqrt{2} M}{n^{3/2}} - \sqrt{\frac{\delta _{M,T}(j,n)}{n}}(x_{j+1}^+ - x_j^-)^{1/2}. \end{aligned}$$

By the Cauchy-Schwartz inequality,

$$\begin{aligned} \sum _{j=k}^{n-1}\sqrt{\frac{\delta _{M,T}(j,n)}{n}}(x_{j+1}^+ - x_j^-)^{1/2}&\le \bigg (\sum _{j=k}^{n-1}\frac{\delta _{M,T}(j,n)}{n} \sum _{i=k}^{n-1}(x_{i+1}^+-x_i^-)\bigg )^{1/2}\\&\le \bigg (\sum _{j=k}^{n-1}\frac{\delta _{M,T}(j,n)}{n} (f_X(1)+1/n)\bigg )^{1/2} \end{aligned}$$

and applying (60), together with the fact that $f_X(1)\le M$, gives

$$\begin{aligned}\bigg (\sum _{j=k}^{n-1}\frac{\delta _{M,T}(j,n)}{n}(f(1)+1/n)\bigg )^{1/2} \le \Big (\frac{28M^5}{n^{1/2}}+\frac{10M^4 n}{T}\Big )^{1/2} \le \frac{6M^{5/2}}{n^{1/4}} + \frac{4M^2n^{1/2}}{T^{1/2}}. \end{aligned}$$

Summing (64) over $k\le j \le \lfloor \theta n\rfloor -1$ and substituting the above bound gives the result. $\square$

We can now prove our main proposition for this section.

#### Proof of Proposition 2.4

We first claim that for each $j=0,\ldots ,n-1$ we have

65 $$\begin{aligned} {\mathcal {E}}^+_X(I_j,\Gamma _{M,T}(f,n),T) \ge \frac{2R_X^-(j)}{n} - 2\sqrt{\frac{2R_X^+(j)}{n}(x^+_{j+1}-x^-_j)} + x^-_{j+1} - x^+_j - \frac{4}{n^2}.\nonumber \\ \end{aligned}$$

Indeed, in either the $X+$ case or the $X-$ case, this follows directly from the definition of ${\mathcal {E}}^+_X$, even without the $4/n^2$ error term on the right-hand side. If we are in neither the $X+$ nor the $X-$ case, then $2R_X^-(j)/n \le x^+_{j+1}-x^-_j$ and $2R_X^+(j)/n \ge x^-_{j+1}-x^+_j$, so

$$\begin{aligned}&\frac{2R_X^-(j)}{n} - 2\sqrt{\frac{2R_X^+(j)}{n}(x^+_{j+1}-x^-_j)} + x^-_{j+1} - x^+_j\\&\hspace{20mm}\le x^+_{j+1}-x^-_j -2\sqrt{\big ((x^-_{j+1} - x^+_j)\vee 0\big )(x^+_{j+1}-x^-_j)} + \big (x^-_{j+1} - x^+_j\big )\vee 0\\&\hspace{20mm}= \Big (\sqrt{x^+_{j+1}-x^-_j} - \sqrt{\big (x^-_{j+1} - x^+_j\big )\vee 0}\Big )^2 \end{aligned}$$

and using $\sqrt{(a-b)\vee 0} \ge a^{1/2}-b^{1/2}$ we have

$$\begin{aligned} \sqrt{\big (x^-_{j+1} - x^+_j\big )\vee 0} \ge \sqrt{\big (x_{j+1}^+-x_j^- -4/n^2\big )\vee 0}\ge \sqrt{x_{j+1}^+-x_j^-} - 2/n, \end{aligned}$$

so in this case

$$\begin{aligned} \frac{2R_X^-(j)}{n} - 2\sqrt{\frac{2R_X^+(j)}{n}(x^+_{j+1}-x^-_j)} + x^-_{j+1} - x^+_j \le \frac{4}{n^2} \le \mathcal E^+_X(I_j,\Gamma _{M,T}(f,n),T) + \frac{4}{n^2} \end{aligned}$$

and the claim is proved.

Now write $L=\lfloor \theta n\rfloor$. By the upper bound in (59), for any $j\ge n^{1/2}$,

$$\begin{aligned} \int \limits _{\frac{j}{n}}^{\frac{j+1}{n}} R_X^*(f(s)) ds \le \frac{R_X^-(j)}{n} + \frac{\delta _{M,T}(j,n)}{n}. \end{aligned}$$

Summing over $k\le j\le L -1$ and applying (60) gives

$$\begin{aligned} \int \limits _{k/n}^{L/n} R_X^*(f(s)) ds \le \sum _{j=k}^{L-1} \frac{R_X^-(j)}{n} + \frac{14M^4}{n^{1/2}} + \frac{5M^3n}{T}. \end{aligned}$$

Lemma A.2 gives that

$$\begin{aligned} \int \limits _{k/n}^{L/n} \sqrt{R_X^*(f(s))f'_X(s)} ds \ge \sum _{j=k}^{L-1} \sqrt{\frac{R_X^+(j)}{n}(x^+_{j+1}-x^-_j)} - \frac{8 M^{5/2}}{n^{1/4}} - \frac{4M^2n^{1/2}}{T^{1/2}}. \end{aligned}$$

Also

$$\begin{aligned} \int \limits _{k/n}^{L/n} f'_X(s) ds \le \sum _{j=k}^{L-1} \big (f({\textstyle {\frac{j+1}{n}}})-f({\textstyle {\frac{j}{n}}})\big )&\le \sum _{j=k}^{L-1} \big (x^-_{j+1}+1/n^2 - x^+_j+1/n^2\big ) \\&\le \sum _{j=k}^{L-1} \big (x^-_{j+1} - x^+_j\big ) + 2/n. \end{aligned}$$

Putting these bounds together with (65), and combining error terms, we obtain

$$\begin{aligned} \int \limits _{k/n}^{L/n} \Big (\sqrt{2R_X^*(f(s))} - \sqrt{f'_X(s)}\Big )^2 ds \le \sum _{j=k}^{L-1} {\mathcal {E}}^+_X(I_j,\Gamma _{M,T}(f,n),T) + O\Big (\frac{M^4}{n^{1/4}} + \frac{M^3n}{T^{1/2}}\Big ), \end{aligned}$$

completing the proof.$\square$

### Proof of Lemma 2.5

The proof of Lemma 2.5 is relatively straightforward. The upper and lower bounds are very similar, but quite lengthy, so we separate them out into two proofs.

#### Proof of Lemma 2.5: upper bound

Write $L=\lfloor \theta n\rfloor$. We split the integral from 0 to $\theta$ into three parts:

66 $$\begin{aligned} \int \limits _0^\theta \! \! R(Tg(s)) ds \le \int \limits _0^{3MT^{-2/3}} \! \! \!\! \! R(Tg(s))ds + \int \limits _{3MT^{-2/3}}^{\lceil \sqrt{n}\rceil /n} \! R(Tg(s))ds + \int \limits _{\lceil \sqrt{n}\rceil /n}^\theta \! \! R(Tg(s))ds.\nonumber \\ \end{aligned}$$

For the first term on the right-hand side, note that for any $g \! \in \! G_{M,T}^2$ and $s\le 3MT^{-2/3}$,

$$\begin{aligned} R(Tg(s)) \le \frac{MT(s + 2T^{-2/3})+1}{1} \le MT(3M+2)T^{-2/3}+1 \le 6M^2T^{1/3}. \end{aligned}$$

For the second term on the right-hand side of (66), we note that for $s>3MT^{-2/3}$ we have $2T^{-2/3}\le \frac{2}{3}s/M$ and therefore, since $g\in G_{M,T}^2$,

$$\begin{aligned} R(Tg(s)) \le \frac{MT(s + 2T^{-2/3})+1}{T(s/M - 2T^{-2/3})} \le \frac{MT(s+\frac{2s}{3M})+1}{T\frac{s}{3M}}&\le 3M^2 \Big (1+\frac{2}{3M}\Big ) + \frac{3M}{Ts} \\  &\le 6M^2 + T^{-1/3}. \end{aligned}$$

We now consider the last term in (66), but work with any $k\ge \lceil \sqrt{n}\rceil$; since $g \! \in \! \Gamma _{M,T}(f,n)$, by definition of $R_X^+$ and $R_Y^+$ we have

67 $$\begin{aligned}&\int \limits _{k/n}^\theta R(Tg(s))ds \nonumber \\&\hspace{15mm}= \int \limits _{k/n}^\theta R_X(Tg(s))ds + \int \limits _{k/n}^\theta R_Y(Tg(s))ds\nonumber \\&\hspace{15mm}\le \sum _{j=k}^{L} \int \limits _{\frac{j}{n}}^{\frac{j+1}{n}} R_X^+(I_j,\Gamma _{M,T}(f,n),T) ds + \sum _{j=k}^{L} \int \limits _{\frac{j}{n}}^{\frac{j+1}{n}} R_Y^+(I_j,\Gamma _{M,T}(f,n),T) ds\nonumber \\&\hspace{15mm}= \sum _{j=k}^{L} \frac{R_X^+(j)}{n} + \sum _{j=k}^{L} \frac{R_Y^+(j)}{n}. \end{aligned}$$

By the lower bound in (59), for any $s\in [\frac{j}{n},\frac{j+1}{n}]$,

$$\begin{aligned} R_X^+(j) \le R_X^*(f(s)) + \delta _{M,T}(j,n), \end{aligned}$$

so using (60) and the fact that *f* is *M*-good,

$$\begin{aligned} \sum _{j=k}^{L} \frac{R_X^+(j)}{n}&\le \int \limits _{k/n}^{(L+1)/n} R_X^*(f(s)) ds + \sum _{j=k}^{L}\frac{\delta _{M,T}(j,n)}{n}\\&\le \int \limits _{k/n}^{(L+1)/n} R_X^*(f(s)) ds + O\Big (\frac{M^4}{n^{1/2}}+\frac{M^3 n}{T}\Big )\\&\le \int \limits _{k/n}^{L/n} R_X^*(f(s)) ds + O\Big (\frac{M^4}{n^{1/2}}+\frac{M^3 n}{T}\Big ). \end{aligned}$$

By symmetry we also have the same statement with *Y* in place of *X*.

Substituting these bounds into (67) gives the upper bound in the second part of the lemma. For the first part of the lemma, returning to (66) and substituting in our estimates above for the three terms on the right-hand side, we have

$$\begin{aligned} \int \limits _0^\theta R(Tg(s)) ds \le \int \limits _{k/n}^{L/n} R^*_X(f(s))ds + O\Big (\frac{M^3}{T^{1/3}} + \frac{M^4}{n^{1/2}} + \frac{1}{T^{1/3}n^{1/2}} + \frac{M^3n}{T}\Big ). \end{aligned}$$

Since $R^*_X(f(s))\ge 0$ for all *s*, the result follows.

#### Proof of Lemma 2.5: lower bound

Note that, again writing $L=\lfloor \theta n\rfloor$ but now with any *k* satisfying $\lceil \sqrt{n}\rceil \le k \le L$,

$$\begin{aligned}\int \limits _{k/n}^\theta R(Tg(s)) ds \ge \int \limits _{k/n}^{L/n} R(Tg(s))ds.\end{aligned}$$

Since $g\in \Gamma _{M,T}(f,n)$, by definition of $R_X^-$ and $R_Y^-$ we have

$$\begin{aligned}&\int \limits _{k/n}^\theta R(Tg(s))ds = \int \limits _{k/n}^\theta R_X(Tg(s))ds + \int \limits _{k/n}^\theta R_Y(Tg(s))ds\\&\quad \ge \sum _{j=k}^{L-1} \int \limits _{\frac{j}{n}}^{\frac{j+1}{n}} R_X^-(I_j,\Gamma _{M,T}(f,n),T) ds + \sum _{j=k}^{L-1} \int \limits _{\frac{j}{n}}^{\frac{j+1}{n}} R_Y^-(I_j,\Gamma _{M,T}(f,n),T) ds\\&\quad = \sum _{j=k}^{L-1} \frac{R_X^-(j)}{n} + \sum _{j=k}^{L-1} \frac{R_Y^-(j)}{n}. \end{aligned}$$

By the upper bound in (59), for any $s\in [\frac{j}{n},\frac{j+1}{n}]$, we have $R_X^-(j) \ge R_X^*(f(s)) - \delta _{M,T}(j,n)$, so using (60) and the fact that *f* is *M*-good,

$$\begin{aligned} \sum _{j=k}^{L-1} \frac{R_X^-(j)}{n}&\ge \int \limits _{k/n}^{L/n} R_X^*(f(s)) ds - \sum _{j=k}^{L-1}\frac{\delta _{M,T}(j,n)}{n} \\  &\ge \int \limits _{k/n}^{L/n} R_X^*(f(s)) ds - O\Big (\frac{M^4}{n^{1/2}} + \frac{M^3n}{T}\Big ). \end{aligned}$$

By symmetry we also have

$$\begin{aligned} \sum _{j=k}^{L-1} \frac{R_Y^-(j)}{n} \ge \int \limits _{k/n}^{L/n} R_Y^*(f(s)) ds - O\Big (\frac{M^4}{n^{1/2}} + \frac{M^3n}{T}\Big ). \end{aligned}$$

Combining these bounds gives the result.$\square$

### Proof of Lemma 3.12

The main difference between Lemma 3.12 and our previous deterministic bounds on the rate function is that it requires us to consider more general time intervals than those of the form $[j/n, (j+1)/n]$. Lemma  will do most of the work required, and uses the uniform structure of $\Lambda _{M,T}(f,n)$ to get better bounds than are possible for $\Gamma _{M,T}(f,n)$.

#### Lemma A.3

Suppose that $M,T>1$, $n\ge 2\,M$ and $f\in PL_n^2 \cap G_M^2$. Then for any $j\in \{\lceil \sqrt{n}\rceil ,\ldots ,n-1\}$ and *u*, *v* such that $\frac{j}{n}\le u<v\le \frac{j+1}{n}$,

$$\begin{aligned} \int \limits _u^v \Big (\sqrt{2R^*_X(f(s))} - \sqrt{f_X'(s)}\Big )^2 ds&\le {\mathcal {E}}_X^+\big ([u,v],\Lambda _{M,T}(f,n),T\big ) + \frac{6\delta _{M,T}(j,n)}{n} \\&+2\sqrt{\frac{2\delta _{M,T}(j,n)}{n}\big (f_X({\textstyle {\frac{j+1}{n}}})-f_X({\textstyle {\frac{j}{n}}})\big )} + \frac{14M}{n^{3/2}}. \end{aligned}$$

#### Proof

As in the proof of Lemma 3.8, for $I\subset [0,1]$ we write ${\hat{R}}^-_X(I)$ as shorthand for the quantity $R^-_X(I,\Lambda _{M,T}(f,n),T)$, and similarly for ${\hat{R}}^+_X(I)$, ${\hat{R}}^-_Y(I)$ and ${\hat{R}}^+_Y(I)$. We also write, for $s\in [0,1]$,

$$\begin{aligned} x^-(s) = x^-(s,\Lambda _{M,T}(f,n)) = \inf \{g_X(s): g\in \Lambda _{M,T}(f,n)\} \end{aligned}$$

and similarly for $x^+(s)$, $y^-(s)$ and $y^+(s)$.

By (18) and the fact that *f* is linear on $I_j$ (and therefore on [*u*, *v*]), we have

68 $$\begin{aligned}&\int \limits _u^v \Big (\sqrt{2R^*_X(f(s))}-\sqrt{f'_X(s)}\Big )^2 ds\nonumber \\&\quad = 2\int \limits _u^v R^*_X(f(s)) ds + \int \limits _u^v f_X'(s) ds - 2\int \limits _u^v \sqrt{2R^*_X(f(s))f_X'(s)} ds\nonumber \\&\quad \le 2{\hat{R}}_X^-(I_j)(v-u) + 2\delta _{M,T}(j,n)(v-u) + f_X(v)-f_X(u) \nonumber \\&\qquad - 2\int \limits _u^v \sqrt{2R^*_X(f(s))\frac{f_X(v)-f_X(u)}{v-u}}ds. \end{aligned}$$

Applying (18) and using the elementary inequality $\sqrt{(a-b)\vee 0} \ge \sqrt{a} -\sqrt{b}$, valid for all $a,b\ge 0$, for any $s\in I_j$ we have

$$\begin{aligned} \sqrt{R^*_X(f(s))}&\ge \sqrt{\big ({\hat{R}}_X^+(I_j)-\delta _{M,T}(j,n)\big )\vee 0}\\&\ge \sqrt{{\hat{R}}_X^+(I_j)} - \sqrt{\delta _{M,T}(j,n)} \\&\ge \sqrt{{\hat{R}}_X^+([u,v])} - \sqrt{\delta _{M,T}(j,n)} \end{aligned}$$

so we have

69 $$\begin{aligned}&\int \limits _u^v \sqrt{2R^*_X(f(s))\frac{f_X(v)-f_X(u)}{v-u}}ds \nonumber \\&\ge \int \limits _u^v \Big (\sqrt{2{\hat{R}}_X^+([u,v])} - \sqrt{2\delta _{M,T}(j,n)}\Big )\sqrt{\frac{f_X(v)-f_X(u)}{v-u}}ds \nonumber \\&\ge \sqrt{2{\hat{R}}_X^+([u,v]) (f_X(v)-f_X(u))(v-u)}- \sqrt{ \frac{2\delta _{M,T}(j,n)}{n} \big (f_X({\textstyle {\frac{j+1}{n}}})-f_X({\textstyle {\frac{j}{n}}})\big )}. \end{aligned}$$

Using again that $\sqrt{(a-b)\vee 0} \ge \sqrt{a} -\sqrt{b}$ we have

$$\begin{aligned} \sqrt{f_X(v)-f_X(u)}&\ge \sqrt{(x^+(v) - 1/n^2 - (x^-(u)+1/n^2))\vee 0} \\  &\ge \sqrt{x^+(v)-x^-(u)} - \sqrt{2/n^2}, \end{aligned}$$

and since *f* is *M*-good, and therefore by (18) $R^+_X([u,v]) \le M^2+\delta _{M,T}(j,n)$, we deduce that

$$\begin{aligned}  &   \sqrt{2{\hat{R}}_X^+([u,v]) (f_X(v)-f_X(u))(v-u)} \\  &   \ge \sqrt{2\hat{R}_X^+([u,v]) (x^+(v)-x^-(u))(v-u)} - \frac{2 \sqrt{M^2+\delta _{M,T}(j,n)}}{n^{3/2}}. \end{aligned}$$

Substituting this into (69), and using that

$$\begin{aligned} \sqrt{M^2+\delta _{M,T}(j,n)} \le \sqrt{M^2} + \sqrt{\delta _{M,T}(j,n)} \le M + \delta _{M,T}(j,n) + 1 \end{aligned}$$

gives that

$$\begin{aligned}&\int \limits _u^v \sqrt{2R^*_X(f(s))\frac{f_X(v)-f_X(u)}{v-u}}ds \\&\quad \ge \sqrt{2{\hat{R}}_X^+([u,v])(v-u)(x^+(v)-x^-(u))} - \sqrt{ {\textstyle {\frac{2\delta _{M,T}(j,n)}{n}}} \big (f_X({\textstyle {\frac{j+1}{n}}})-f_X({\textstyle {\frac{j}{n}}})\big )} \\&\qquad \ - \frac{2(M+\delta _{M,T}(j,n)+1)}{n^{3/2}}. \end{aligned}$$

Substituting this bound into (68) and using that ${\hat{R}}_X^-(I_j)\le {\hat{R}}_X^-([u,v])$ and $v-u\le 1/n$, we obtain

$$\begin{aligned}&\int \limits _u^v \Big (\sqrt{2R^*_X(f(s))}-\sqrt{f'_X(s)}\Big )^2 ds\\&\quad \ \le 2{\hat{R}}_X^-([u,v])(v-u) + x^-(v) - x^+(u) - 2\sqrt{2\hat{R}_X^+([u,v])(v-u)(x^+(v)-x^-(u))}\\&\qquad \qquad \qquad + \frac{6\delta _{M,T}(j,n)}{n} + \frac{2}{n^2} + 2\sqrt{\frac{2\delta _{M,T}(j,n)}{n}\big (f_X({\textstyle {\frac{j+1}{n}}})-f_X({\textstyle {\frac{j}{n}}})\big )} + \frac{8M}{n^{3/2}}. \end{aligned}$$

It then remains to note that, following exactly the same argument as (65),

$$\begin{aligned}  &   {\mathcal {E}}_X^+\big ([u,v],\Lambda _{M,T}(f,n),T\big )\ge 2{\hat{R}}_X^-([u,v])(v-u) + x^-(v) - x^+(u) \\  &   - 2\sqrt{2{\hat{R}}_X^+([u,v])(v-u)(x^+(v)-x^-(u))} - 4/n^2. \end{aligned}$$

Combining error terms gives the result. $\square$

It is now a relatively simple task to apply Lemma  to complete the proof of Lemma 3.12.

#### Proof of Lemma 3.12

By symmetry it suffices to show that

$$\begin{aligned}  &   \sum _{j=\lfloor an\rfloor }^{\lceil bn\rceil -1} \mathcal E_X^+(I_j\cap [a,b],\Lambda _{M,T}(f,n),T) \\  &   \quad \ge \int \limits _a^b \Big (\sqrt{2R^*_X(f(s))} - \sqrt{f'_X(s)}\Big )^2 ds - O\Big (\frac{M^4}{n^{1/4}}+\frac{M^3 n}{T^{1/2}}\Big ). \end{aligned}$$

By Lemma ,

$$\begin{aligned}&\sum _{j=\lfloor an\rfloor }^{\lceil bn\rceil -1} \mathcal E_X^+(I_j\cap [a,b],\Lambda _{M,T}(f,n),T)\\&\hspace{10mm}\ge \sum _{j=\lfloor an\rfloor }^{\lceil bn\rceil -1} \int \limits _{I_j\cap [a,b]} \Big (\sqrt{2R^*_X(f(s))} - \sqrt{f'_X(s)}\Big )^2 ds\\&\hspace{10mm} - \sum _{j=\lfloor an\rfloor }^{\lceil bn\rceil -1}\Bigg (\frac{6\delta _{M,T}(j,n)}{n} + 2\sqrt{\frac{2\delta _{M,T}(j,n)}{n}\big (f_X({\textstyle {\frac{j+1}{n}}})-f_X({\textstyle {\frac{j}{n}}})\big )} + \frac{14M}{n^{3/2}}\Bigg ). \end{aligned}$$

Note that

$$\begin{aligned} \sum _{j=\lfloor an\rfloor }^{\lceil bn\rceil -1} \int \limits _{I_j\cap [a,b]} \Big (\sqrt{2R^*_X(f(s))} - \sqrt{f'_X(s)}\Big )^2 ds = \int \limits _a^b \Big (\sqrt{2R^*_X(f(s))} - \sqrt{f'_X(s)}\Big )^2 ds, \end{aligned}$$

and by (60)

$$\begin{aligned} \sum _{j=\lfloor an\rfloor }^{\lceil bn\rceil -1} \Big (\frac{6\delta _{M,T}(j,n)}{n} + \frac{14M}{n^{3/2}}\Big ) = O\Big (\frac{M^4}{n^{1/2}} + \frac{M^3n}{T}\Big ). \end{aligned}$$

Finally, by Cauchy–Schwarz,

$$\begin{aligned}  &   \sum _{j=\lfloor an\rfloor }^{\lceil bn\rceil -1} \sqrt{\frac{2\delta _{M,T}(j,n)}{n}\big (f_X({\textstyle {\frac{j+1}{n}}})-f_X({\textstyle {\frac{j}{n}}})\big )}\\  &   \le \bigg (\sum _{i=\lfloor an\rfloor }^{\lceil bn\rceil -1} \frac{2\delta _{M,T}(i,n)}{n} \sum _{j=\lfloor an\rfloor }^{\lceil bn\rceil -1} \big (f_X({\textstyle {\frac{j+1}{n}}})-f_X({\textstyle {\frac{j}{n}}})\big )\bigg )^{1/2}, \end{aligned}$$

and using (60) and the fact that $f\in G_M^2$, we see that

$$\begin{aligned} \sum _{j=\lfloor an\rfloor }^{\lceil bn\rceil -1} \sqrt{\frac{2\delta _{M,T}(j,n)}{n}\big (f_X({\textstyle {\frac{j+1}{n}}})-f_X({\textstyle {\frac{j}{n}}})\big )} = O\Big (\frac{M^{5/2}}{n^{1/4}}+\frac{M^2 n^{1/2}}{T^{1/2}}\Big ). \end{aligned}$$

Combining these estimates completes the proof.$\square$

## Elementary bounds on compound Poisson processes: proof of Lemmas 6.1 and 6.2

The proof of Lemma 6.1 is completely standard; it simply involves applying a Chernoff bound, with some case analysis depending on whether we are in the $X-$ or $X+$ case, or neither, and also whether we are in the $Y-$ or $Y+$ case, or neither.

### Proof of Lemma 6.1

For $z\in V(I,F)$, using (ii), (i) and (iii) in that order,

$$\begin{aligned}&\mathbb {Q}\big ({\mathcal {A}}_\xi (I,F,T) \,\big |\,\xi ^T(I^-)=z\big ) \\&\hspace{20mm}= Q^{I,F,T}_z\big ({\mathcal {A}}(I,F,T) \big )\\&\hspace{20mm}\le Q^{I,F,T}_z\big (X_-(I^+)\le x^+(I^+,F),\; Y_-(I^+)\le y^+(I^+,F) \big )\\&\hspace{20mm}= Q^{I,F,T}_z\big (X_-(I^+)\le x^+(I^+,F)\big )Q^{I,F,T}_z\big (Y_-(I^+)\le y^+(I^+,F) \big ). \end{aligned}$$

We will apply this bound when we are in the $X-$ and $Y-$ cases. Of course, we were not forced to concentrate on the two upper boundaries $x^+(I^+,F)$ and $y^+(I^+,F)$, and by considering the other permutations of boundaries we obtain upper bounds on the same quantity of the form

$$\begin{aligned}Q^{I,F,T}_z\big (X_+(I^+)\ge x^-(I^+,F)\big )Q^{I,F,T}_z\big (Y_+(I^+)\ge y^-(I^+,F) \big ),\\Q^{I,F,T}_z\big (X_+(I^+)\ge x^-(I^+,F)\big )Q^{I,F,T}_z\big (Y_-(I^+)\le y^+(I^+,F) \big )\end{aligned}$$

and

$$\begin{aligned}Q^{I,F,T}_z\big (X_-(I^+)\le x^+(I^+,F)\big )Q^{I,F,T}_z\big (Y_+(I^+)\ge y^-(I^+,F) \big )\end{aligned}$$

which we can apply in other cases as appropriate.

Now, for any $\lambda >0$, by Markov’s inequality,

$$\begin{aligned}&Q^{I,F,T}_z\big (X_-(I^+)\le x^+(I^+,F)\big ) \\&\hspace{20mm} = Q^{I,F,T}_z\big (e^{-\lambda X_-(I^+)} \ge e^{-\lambda x^+(I^+,F)}\big )\\&\hspace{20mm} \le Q^{I,F,T}_z\big [e^{-\lambda (X_-(I^+)-X_-(I^-))}\big ]e^{\lambda (x^+(I^+,F) - x^-(I^-,F))}\\&\hspace{20mm} = \exp \Big ( \! -2R^-_X(I,F,T)T|I|\frac{\lambda }{T+\lambda }+\lambda (x^+(I^+,F) - x^-(I^-,F))\Big ). \end{aligned}$$

In the $X-$ case we have $2R^-_X(I,F,T)|I| > x^+(I^+,F) - x^-(I^-,F)$, so we can choose the optimal value

$$\begin{aligned} \lambda = T\sqrt{\frac{2R^-_X(I,F,T)|I|}{x^+(I^+,F) - x^-(I^-,F)}} - T >0. \end{aligned}$$

Simplifying gives

$$\begin{aligned}  &   Q^{I,F,T}_z\big (X_-(I^+) \le x^+(I^+,F)\big ) \\  &   \le \exp \Big ( \! -T\Big (\sqrt{2R^-_X(I,F,T)|I|}-\sqrt{x^+(I^+,F) - x^-(I^-,F)}\Big )^2\Big ) \end{aligned}$$

which equals $\exp \big (-T{\mathcal {E}}^+_X(I,F,T)\big )$ in the $X-$ case. (The reader should not be surprised to note that this is where the Fenchel–Legendre transform of an appropriate compound Poisson process appears, in this case $\Lambda (2R_X^-(I,F,T)|I|, x^+(I^+,F) - x^-(I^-,F))$ where $\Lambda (r,x)$ was defined in Sect. 1.5.)

Similarly, in the $X+$ case, by using

$$\begin{aligned}  &   Q^{I,F,T}_z\big (X_+(I^+)\ge x^-(I^+,F)\big ) \\  &   \le Q^{I,F,T}_z[\exp \big (\mu (X_+(I^+)-X_+(I^-))\big ) - \mu (x^-(I^+,F) - x^+(I^-,F))] \end{aligned}$$

for $\mu >0$, we obtain

$$\begin{aligned}&Q^{I,F,T}_z\big (X_+(I^+)\ge x^-(I^+,F)\big ) \\&\hspace{20mm} \le \exp \Big (\! -T\Big (\sqrt{2R^+_X(I,F,T)|I|}-\sqrt{x^-(I^+,F) - x^+(I^-,F)}\Big )^2\Big )\\&\hspace{20mm} = \exp \big ( \! -T{\mathcal {E}}^+_X(I,F,T)\big ) \end{aligned}$$

and when we are in neither the $X-$ nor $X+$ case we can use a trivial upper bound of 1. By symmetry we obtain the same bounds in terms of *Y*. Applying these bounds in the appropriate cases completes the proof.

Before proving Lemma 6.2, the bulk of the work is done by the following intermediate result.

### Lemma B.1

Suppose that $\delta ,t,A>0$ and $R\ge 1/2$. Let $(X(s),s\ge 0)$ be a compound Poisson process of rate *RT* whose jumps are exponentially distributed with parameter *T*. Then for any *T*,

$$\begin{aligned} \mathbb {P}(|X(s)-As|<\delta \,\, \forall s\le t) \le \exp \Big (-tT(\sqrt{R} - \sqrt{A})^2 + \delta \big |1-\sqrt{R/A}\big | T\Big ), \end{aligned}$$

and for $$T>\frac{2A^{3/2}(4t+\delta )}{R^{1/2}\delta ^{2}(A\wedge 1)^2}$$,

$$\begin{aligned} \mathbb {P}(|X(s)-As|<\delta \,\, \forall s\le t)\ge \frac{1}{2}\exp \Big (-tT(\sqrt{R} - \sqrt{A})^2 - \delta (A\wedge 1) \big |1-\sqrt{R/A}\big | T\Big ). \end{aligned}$$

### Proof

For any $q<T$ and $s\ge 0$, we have

$$\begin{aligned} \mathbb {E}[e^{qX(s)}] = \exp \Big (\frac{Rqs}{1-q/T}\Big ). \end{aligned}$$

Fix $a= T(1-\sqrt{R/A})$; then elementary calculations show that

$$\begin{aligned} \frac{\mathbb {E}[X(s)e^{aX(s)}]}{\mathbb {E}[e^{aX(s)}]} = As. \end{aligned}$$

Let $(\sigma _s)_{s\ge 0}$ be the natural filtration of *X*, and define a new probability measure $\mu$ by setting

$$\begin{aligned} \frac{d\mu }{d\mathbb {P}}\Big |_{\sigma _s} = \frac{e^{aX(s)}}{\mathbb {E}[e^{aX(s)}]} = \exp \Big (aX(s)-\frac{Ras}{1-a/T}\Big ). \end{aligned}$$

Then, by the definition of $\mu$, for any $\delta '>0$ we have

$$\begin{aligned} \mathbb {P}(|X(s)-As|<\delta '\,\,\,\,\forall s\le t) = \mu \Big [\exp \Big (-aX(t)+\frac{Rat}{1-a/T}\Big )\mathbbm {1}_{\{|X(s)-As|<\delta '\,\,\,\,\forall s\le t\}}\Big ] \end{aligned}$$

and using the bound $|X(t)-At|<\delta '$ and simplifying we obtain

70 $$\begin{aligned}  &   \exp \Big (-tT(\sqrt{R} - \sqrt{A})^2 - |a|\delta ')\Big )\mu (|X(s)-As|<\delta '\,\,\,\,\forall s\le t)\nonumber \\  &   \le \mathbb {P}(|X(s)-As|<\delta '\,\,\,\,\forall s\le t)\nonumber \\  &   \le \exp \Big (-tT(\sqrt{R} - \sqrt{A})^2 + |a|\delta ')\Big )\mu (|X(s)-As|<\delta '\,\,\,\,\forall s\le t). \end{aligned}$$

The required upper bound follows immediately by taking $\delta '=\delta$. For the lower bound we take $\delta ' = \delta (A\wedge 1)$, and then it remains to bound $\mu (|X(s)-As|<\delta (A\wedge 1)\,\,\,\,\forall s\le t)$ from below.

One may easily check that $(X(s)-As,s\ge 0)$ is a martingale under $\mu$, and therefore by Jensen’s inequality, $(e^{\nu (X(s)-As)},s\ge 0)$ is a submartingale under $\mu$ for any $\nu <T-a$ (the upper bound on $\nu$ is required to ensure that the expectation is finite). By Doob’s submartingale inequality, for any $\nu \in (0,T-a)$, we have

71 $$\begin{aligned} \mu (\exists s\le t : X(s)-As\ge \delta (A\wedge 1))&= \mu \Big (\sup _{s\le t}e^{\nu (X(s)-As)} \ge e^{\nu \delta (A\wedge 1)}\Big ) \nonumber \\&\le \mu [e^{\nu (X(t)-At)}]e^{-\nu \delta (A\wedge 1)} \end{aligned}$$

and for any $\nu <0$ we have

72 $$\begin{aligned} \mu (\exists s\le t : X(s)-As\le -\delta (A\wedge 1))&= \mu \Big (\sup _{s\le t}e^{\nu (X(s)-As)} \ge e^{-\nu \delta (A\wedge 1)}\Big ) \nonumber \\&\le \mu [e^{\nu (X(t)-At)}]e^{\nu \delta (A\wedge 1)}. \end{aligned}$$

Now, for any $\nu <T-a$,

$$\begin{aligned} \mu [e^{\nu (X(t)-At)}]&= \mathbb {E}[e^{(a+\nu )X(t)}]e^{-Rat/(1-a/T)-A\nu t}\\&= \exp \Big (\frac{R(a+\nu )t}{1-(a+\nu )/T} - \frac{Rat}{1-a/T} - A\nu t\Big ), \end{aligned}$$

and simplifying we obtain

$$\begin{aligned} \mu [e^{\nu (X(t)-At)}] = \exp \Big (\frac{A\nu t}{1-\frac{\nu }{T}(A/R)^{1/2}}-A\nu t\Big ) = \exp \Big (\frac{A\nu ^2 t}{(R/A)^{1/2}T-\nu }\Big ). \end{aligned}$$

It is then easy to check that for $T>\frac{2A^{3/2}(4t+\delta )}{R^{1/2}\delta ^{2}(A\wedge 1)^2}$, each of the probabilities in (71) and (72) can be made smaller than $e^{-3/2}<1/4$ by choosing $\nu =\pm \frac{2}{\delta (A\wedge 1)}$. Thus we have

$$\begin{aligned} \mu (|X(s)-As|<\delta (A\wedge 1)\,\,\,\,\forall s\le t) \ge 1/2 \end{aligned}$$

for such *T*. Substituting this into the lower bound in (70), using $\delta '=\delta (A\wedge 1)$, gives the result. $\square$

Lemma 6.2 now requires us to deal with the position of our process at the endpoints of the intervals $I_j$.

### Proof of Lemma 6.2

Let $A_t(a) = A-a/t$. Note that if $|X(s)-A_t(a)s|<\delta /2$ for all $s\le t$, then $|a+X(s)-As|<\delta$ for all $s\le t$ and $|a+X(t)-At|<\delta /2$. Thus

$$\begin{aligned}  &   \mathbb {P}(|a+X(s)-As|<\delta \,\, \forall s\le t, \,\, |a+X(t)-At|<\delta /2) \\  &   \quad \ge \mathbb {P}(|X(s)-A_t(a)s|<\delta /2 \,\, \forall s\le t). \end{aligned}$$

Lemma B.1 tells us that the latter probability is at least

73 $$\begin{aligned} \frac{1}{2}\exp \bigg (-tT(\sqrt{R} - \sqrt{A_t(a)})^2 - \frac{\delta (A_t(a)\wedge 1)}{2} \Big |1-\Big (\frac{R}{A_t(a)}\Big )^{1/2}\Big | T\bigg ). \end{aligned}$$

Using the fact that $(1-x)^{1/2}\ge 1-x^{1/2}$ for $x\in [0,1]$, we have

$$\begin{aligned} t(\sqrt{R} - \sqrt{A_t(a)})^2&\le \Big (R + A+\frac{\delta }{2t}-2\sqrt{AR}\Big (1-\frac{\delta }{2tA}\Big )^{1/2}\Big )t\\&\le (\sqrt{R} - \sqrt{A})^2 t + \frac{\delta }{2} + \sqrt{2\delta Rt} \end{aligned}$$

and

$$\begin{aligned} \frac{\delta (A_t(a)\wedge 1)}{2}\Big |1-\Big (\frac{R}{A_t(a)}\Big )^{1/2}\Big | \le \frac{\delta }{2} \Big (1+\Big (\frac{R}{A_t(a)}\Big )^{1/2}\Big ) \le \frac{\delta }{2}(1+\sqrt{R}). \end{aligned}$$

Substituting these estimates into (73) gives

$$\begin{aligned} \frac{1}{2}\exp \bigg (-tT(\sqrt{R} - \sqrt{A})^2 - \delta \Big (1+\sqrt{R}\big (\sqrt{2t/\delta }+1/2\big )\Big ) T\bigg ), \end{aligned}$$

as required.$$\square $$

## Proofs of compactness and semicontinuity

### Compactness of $G_{M,T}^2$: proof of Lemma 7.1

The proof of Lemma 7.1, which says that for any $F\subset E^2$ we can cover $F\cap G_{M,T}^2$ in a nice way with small balls around piecewise linear functions, is straightforward. We directly construct piecewise linear approximations to an arbitrary function within $F\cap G_{M,T}^2$.

#### Proof of Lemma 7.1

Suppose that $T\ge (4Mn)^{3/2}$ and take $h\in F\cap G_{M,T}^2$. Then define a function $g\in {{\,\textrm{PL}\,}}_{n}^2$ by interpolating linearly between the values

$$\begin{aligned} g(j/n) = \lfloor n^2 h(j/n)\rfloor /n^2, \hspace{5mm} j=0,1,\ldots ,n. \end{aligned}$$

Then clearly

$$\begin{aligned} \Delta _n(g,h) < 1/n^2. \end{aligned}$$

We claim that $d(g,h)\le 1/n$. To see this, take $s\in [0,1]$, and then fix $j\in \{0,1,\ldots ,n-1\}$ such that $s\in [j/n,(j+1)/n]$. Then

$$\begin{aligned} g(s)\le g({\textstyle {\frac{j+1}{n}}}) \le h({\textstyle {\frac{j+1}{n}}}) \end{aligned}$$

and

$$\begin{aligned} g(s) \ge g({\textstyle {\frac{j}{n}}}) \ge h({\textstyle {\frac{j}{n}}})- 1/n^2 \end{aligned}$$

which, by the definition (2) of *d*, establishes the claim.

Next we claim that $g\in G_{4\,M}^2$. Since $h\in G_{M,T}^2$ we know that for any $j=1,2,\ldots ,n$,

$$\begin{aligned}\frac{j}{Mn} - 2T^{-2/3} \le h(j/n) \le M\Big (\frac{j}{n}+2T^{-2/3}\Big )\end{aligned}$$

and since $T\ge (4Mn)^{3/2}$ we obtain

$$\begin{aligned}\frac{j}{2Mn} \le \frac{j-1/2}{Mn} \le h(j/n) \le \frac{Mj+1/2}{n} \le \frac{2Mj}{n}.\end{aligned}$$

But $$h(j/n)-1/n^2\le g(j/n)\le h(j/n)$$ so

$$\begin{aligned}\frac{j}{2Mn} - \frac{1}{n^2} \le g(j/n) \le \frac{2Mj}{n}\end{aligned}$$

and since $n\ge 4\,M$, $j/(2Mn)-1/n^2 \ge j/(4Mn)$, which, given that *g* interpolates linearly between these values, proves the claim.

Since the functions *g* created in this way can take only finitely many values (namely integer multiples of $1/n^2$ with a maximum of at most 2*M*) at the times $0,1/n,2/n,\ldots ,1$, and interpolate linearly between these values, there are only finitely many possible such functions, and therefore the proof is complete.$\square$

### Partial lower semi-continuity of *K*: proof of Proposition 5.9

To complete the proof of the lower bound in Sect. 3, we need to prove a partial semi-continuity result about *K*, which was stated in Proposition 5.9. We begin with a useful lemma which states that given continuity, convergence under *d* implies convergence pointwise.

#### Lemma C.1

If $f\in E$ is continuous at *s* and $d(f_n,f)\rightarrow 0$, then $f_n(s)\rightarrow f(s)$. Moreover, if $d(f_n,f)\rightarrow 0$, then $f_n(1)\rightarrow f(1)$ (regardless of whether *f* is continuous at 1).

#### Proof

Fix $\varepsilon >0$ and $s\in [0,1]$ such that *f* is continuous at *s*. Then we can find $\delta >0$ such that $|f(u)-f(s)|<\varepsilon /2$ for any $u\in [s-\delta ,s+\delta ]\cap [0,1]$. Choose *N* such that $d(f_n,f)<(\varepsilon /2)\wedge \delta$ for all $n\ge N$. By the definition (2) of *d*, this means that

$$\begin{aligned}f((s-\delta )\vee 0) - \varepsilon /2 \le f_n(s) \le f((s+\delta )\wedge 1) + \varepsilon /2.\end{aligned}$$

Then we have

$$\begin{aligned}f(s)-\varepsilon \le f((s-\delta )\vee 0) - \varepsilon /2 \le f_n(s) \le f((s+\delta )\wedge 1) + \varepsilon /2 \le f(s)+\varepsilon \end{aligned}$$

and since $\varepsilon >0$ was arbitrary, we have shown that $f_n(s)\rightarrow f(s)$.

For the second part of the lemma, simply note that by the definition of *d*, if $d(f_n,f)<\varepsilon$ then $|f_n(1)-f(1)|<\varepsilon$. $\square$

We now show that when $f_n$ is the piecewise linear interpolation to *f*, the cross-terms that appear when multiplying out the quadratic terms in *K* satisfy a semicontinuity property.

#### Lemma C.2

Suppose that $0\le a<b\le 1$ and that $f\in G_M^2$ for some *M*. Let $f_n$ be the function in ${{\,\textrm{PL}\,}}_n$ constructed by setting $f_n(j/n)=f(j/n)$ for each $j=0,\ldots ,n$ and interpolating linearly. Then

$$\begin{aligned}\liminf _{n\rightarrow \infty } \int \limits _a^b \sqrt{R^*_X(f_n(s)) f_{n,X}'(s)} \,\text {d}s \ge \int \limits _a^b \sqrt{R^*_X(f(s))f_X'(s)}\,\text {d}s\end{aligned}$$

where we write $f_n(s) = (f_{n,X}(s),f_{n,Y}(s))$.

#### Proof

We carry out the proof when $a=0$ and $b=1$; the general case follows by including $\mathbbm {1}_{\{s\in [a,b]\}}$ throughout.

Note that

$$\begin{aligned}&\int \limits _0^1 \sqrt{R^*_X(f_n(s)) f_{n,X}'(s)} \, ds \\&\hspace{10mm}= \int \limits _0^1 \sum _{i=1}^n \mathbbm {1}_{\{s\in [\frac{i-1}{n},\frac{i}{n})\}} \sqrt{R^*_X(f_n(s))} \sqrt{n(f_{n,X}({\textstyle {\frac{i}{n}}})-f_{n,X}({\textstyle {\frac{i-1}{n}}}))}\, ds\\&\hspace{10mm}= \int \limits _0^1 \sum _{i=1}^n \mathbbm {1}_{\{s\in [\frac{i-1}{n},\frac{i}{n})\}} \sqrt{R^*_X(f_n(s))} \sqrt{n(f_X({\textstyle {\frac{i}{n}}})-f_X({\textstyle {\frac{i-1}{n}}}))}\, ds\\&\hspace{10mm}\ge \int \limits _0^1 \sum _{i=1}^n \mathbbm {1}_{\{s\in [\frac{i-1}{n},\frac{i}{n})\}}\inf _{u\in [\frac{i-1}{n},\frac{i}{n}]} \sqrt{R^*_X(f_n(u))}\sqrt{n(f_X({\textstyle {\frac{i}{n}}})-f_X({\textstyle {\frac{i-1}{n}}}))}\,ds. \end{aligned}$$

Since *f* is continuous almost everywhere, by Lemma C.1, $f_n(u)\rightarrow f(u)$ almost everywhere. Since *f* is *M*-good, and $R^*_X$ is continuous away from 0, $R^*_X(f_n(u))\rightarrow R^*_X(f(u))$ for almost every $u\in [0,1]$. Since *f* is differentiable almost everywhere, we deduce that the integrand above converges to $\sqrt{R^*_X(f(s))f_X'(s)}$ for almost every $s\in [0,1]$. It is also bounded above by

$$\begin{aligned} F_n(s) = \sum _{i=1}^n \mathbbm {1}_{\{s\in [\frac{i-1}{n},\frac{i}{n})\}} M \bigg (n\Big (f_X\Big (\frac{i}{n}\Big ) - f_X\Big (\frac{i-1}{n}\Big )\Big ) + 1\bigg ) \end{aligned}$$

which is integrable and whose integral equals $M(f_X(1)+1)$ for each *n*, which is also the integral of $\lim _{n\rightarrow \infty } F_n(s)$. Therefore, by the generalised dominated convergence theorem, the integral converges to

$$\begin{aligned}\int \limits _0^1 \sqrt{R^*_X(f(s))f_X'(s)}\, ds\end{aligned}$$

and the proof is complete. $\square$

It is then a simple task to prove Proposition 5.9, which shows that *K*(*f*, 0, *t*) can be bounded above by taking piecewise linear approximations to *f*.

#### Proof of Proposition 5.9

By (7), for any $f\in E^2$,

$$\begin{aligned} K(f,0,t)  &   = -\int \limits _0^t R^*(f(s))ds + 2\sqrt{2}\int \limits _0^t \sqrt{R^*_X(f(s))f'_X(s)}ds - f_X(t)\\  &   \quad + 2\sqrt{2}\int \limits _0^t \sqrt{R^*_Y(f(s))f'_Y(s)}ds - f_Y(t). \end{aligned}$$

It therefore suffices, by symmetry, to show that

$$\begin{aligned}  &   \limsup _{n\rightarrow \infty }\int \limits _0^t R^*(f_n(s))ds \le \int \limits _0^t R^*(f(s))ds,\\  &   \quad \liminf _{n\rightarrow \infty } \int \limits _0^t \sqrt{R^*_X(f_n(s))f'_{n,X}(s)}ds \ge \int \limits _0^t \sqrt{R^*_X(f(s))f'_X(s)}ds\end{aligned}$$

and

$$\begin{aligned}\limsup _{n\rightarrow \infty } f_{n,X}(t) \le f_X(t).\end{aligned}$$

The first of these statements follows from Lemma C.1 and the continuity and boundedness of $R^*$ away from 0, using the fact that *f*, and therefore $f_n$, is good. The second follows from Lemma C.2. For the third, we observe that since *f* is increasing and right-continuous,

$$\begin{aligned}f_{n,X}(t) \le f_X\big ({\textstyle {\frac{\lceil nt\rceil }{n}}}\big ) \rightarrow f_X(t),\end{aligned}$$

which completes the proof.$\square$

### Upper semi-continuity of *K*: proofs of Lemma 7.2 and Proposition 2.10

The following consequence of the Cauchy–Schwarz inequality is the key to proving Lemma 7.2.

#### Lemma C.3

Suppose that $0\le a<b\le 1$ and $f,f_n\in G_M^2$ for all *n*. If *f* is differentiable on [*a*, *b*], and $d(f_n,f)\rightarrow 0$, then

$$\begin{aligned}\limsup _{n\rightarrow \infty } \int \limits _a^b \sqrt{R^*_X(f_n(s)) f_{n,X}'(s)} \,\text {d}s \le \int \limits _a^b \sqrt{R^*_X(f(s))f_X'(s)}\,\text {d}s\end{aligned}$$

where we write $f_{n,X}$ for the *x*-component of $f_n$.

#### Proof

We carry out the proof when $a=0$ and $b=1$; the general case follows by including $\mathbbm {1}_{\{s\in [a,b]\}}$ throughout. By the Cauchy–Schwarz inequality, for any $m\in \mathbb {N}$,

$$\begin{aligned}&\int \limits _0^1 \sqrt{R^*_X(f_n(s)) f_{n,X}'(s)} ds = \sum _{i=1}^m \int \limits _{(i-1)/m}^{i/m} \sqrt{R^*_X(f_n(s)) f_{n,X}'(s)} ds\\&\quad \le \sum _{i=1}^m \bigg ( \int \limits _{(i-1)/m}^{i/m} R^*_X(f_n(s))ds \bigg )^{1/2}\bigg (\int \limits _{(i-1)/m}^{i/m} f'_{n,X}(s)ds \bigg )^{1/2}\\&\quad \le \sum _{i=1}^m \bigg (\int \limits _{(i-1)/m}^{i/m} R^*_X(f_n(s)) ds\bigg )^{1/2}\big (f_{n,X}\big ({\textstyle {\frac{i}{m}}}\big ) - f_{n,X}\big ({\textstyle {\frac{i-1}{m}}}\big )\big )^{1/2} \end{aligned}$$

where the last inequality is not an equality since we do not know whether $f_{n,X}$ is absolutely continuous.

Since *f* is continuous, by Lemma C.1 we know that $f_n(s)\rightarrow f(s)$ for every *s*. Thus, using that $f,f_n\in G_M^2$ and $R^*_X$ is continuous away from 0, by bounded convergence the right-hand side above converges to

$$\begin{aligned} \sum _{i=1}^m \bigg (\int \limits _{(i-1)/m}^{i/m} R^*_X(f(s)) ds\bigg )^{1/2}\Big (f_{X}\Big (\frac{i}{m}\Big ) - f_{X}\Big (\frac{i-1}{m}\Big )\Big )^{1/2} \end{aligned}$$

which is at most

74 $$\begin{aligned} \sum _{i=1}^m \frac{1}{m} \bigg (\sup _{u\in [\frac{i-1}{m},\frac{i}{m}]} R^*_X(f(u))\bigg )^{1/2} \bigg ( m\Big (f_X\Big (\frac{i}{m}\Big ) - f_X\Big (\frac{i-1}{m}\Big )\Big )\bigg )^{1/2}. \end{aligned}$$

We claim that (74) converges, as $m\rightarrow \infty$, to $\int _0^1 \sqrt{R^*_X(f(s))f'_X(s)} ds$. To prove this we can follow almost exactly the same argument as in the proof of Lemma C.2, writing (74) in the form

$$\begin{aligned} \int \limits _0^1 \sum _{i=1}^m \mathbbm {1}_{\{s\in [\frac{i-1}{m},\frac{i}{m})\}} \bigg (\sup _{u\in [\frac{i-1}{m},\frac{i}{m}]} R^*_X(f(u))\bigg )^{1/2} \bigg ( m\Big (f_X\Big (\frac{i}{m}\Big ) - f_X\Big (\frac{i-1}{m}\Big )\Big )\bigg )^{1/2} ds \end{aligned}$$

and applying the generalised dominated convergence theorem since the integrand evaluated at *s* converges as $m\rightarrow \infty$ to $R^*_X(f(s))f'_X(s)$ for almost every $s\in [0,1]$, and can be bounded above by

$$\begin{aligned} F_m(s) = \sum _{i=1}^m \mathbbm {1}_{\{s\in [\frac{i-1}{m},\frac{i}{m})\}} M \bigg (m\Big (f_X\Big (\frac{i}{m}\Big ) - f_X\Big (\frac{i-1}{m}\Big )\Big ) + 1\bigg ). \end{aligned}$$

This completes the proof. $\square$

The next step is to extend the previous lemma to functions that are not necessarily continuous.

#### Lemma C.4

Suppose that $0\le a<b\le 1$ and $f,f_n\in G_M^2$ for all *n*. If $d(f_n,f)\rightarrow 0$, then

$$\begin{aligned} \limsup _{n\rightarrow \infty } \int \limits _a^b \sqrt{R^*_X(f_n(s)) f_{n,X}'(s)} \,\text {d}s \le \int \limits _a^b \sqrt{R^*_X(f(s))f_X'(s)}\,\text {d}s \end{aligned}$$

where we write $f_{n,X}$ for the *x*-component of $f_n$.

#### Proof

Fix $\varepsilon \in (0,6M)$. Let $S\subset (0,1)$ be the set of points (in (0, 1)) at which *f* is not differentiable. Since *f* is increasing, *S* has zero Lebesgue measure, and can therefore be covered by a finite collection $(s_1^-,s_1^+), \ldots , (s_N^-, s_N^+)$ of open intervals whose total length is at most $\varepsilon ^2/M^3$. Let $S' = \bigcup _{i=1}^N (s_i^-,s_i^+)$. Then by Lemma C.3, since $[a,b]\setminus S'$ is a finite union of closed intervals on which *f* is absolutely continuous, we have

$$\begin{aligned} \limsup _{n\rightarrow \infty } \int \limits _{[a,b]\setminus S'} \sqrt{R^*_X(f_n(s)) f_{n,X}'(s)} ds&\le \int \limits _{[a,b]\setminus S'} \sqrt{R^*_X(f(s))f_X'(s)} ds \\&\le \int \limits _a^b \sqrt{R^*_X(f(s))f_X'(s)} ds. \end{aligned}$$

It therefore suffices to show that

75 $$\begin{aligned} \limsup _{n\rightarrow \infty } \int \limits _{[a,b]\cap S'} \sqrt{R^*_X(f_n(s)) f_{n,X}'(s)} {\text {ds}} \le \varepsilon . \end{aligned}$$

However, since $f_n\in G_M^2$, we have

$$\begin{aligned} \int \limits _{[a,b]\cap S'} \sqrt{R^*_X(f_n(s)) f_{n,X}'(s)} ds \le \int \limits _{[a,b]\cap S'} M\sqrt{f_{n,X}'(s)} ds, \end{aligned}$$

and by Jensen’s inequality, this is at most

$$\begin{aligned} M\sqrt{\big |[a,b]\cap S'\big |} \bigg (\int \limits _{[a,b]\cap S'} f_{n,X}'(s) ds\bigg )^{1/2} \le M\sqrt{|S'|} (f_{n,X}(b)-f_{n,X}(a))^{1/2} \end{aligned}$$

where $|S'|$ denotes the Lebesgue measure of $S'$. Since $f_n\in G_M^2$, this is at most $M^{3/2}\sqrt{|S'|}$, which is smaller than $\varepsilon$ by construction. Thus (75) holds and the proof is complete. $\square$

The proof of Lemma 7.2 is now a simple consequence of the results above.

#### Proof of Lemma 7.2

We use the alternative form of *K* mentioned in (7), i.e.

76 $$\begin{aligned} K(f,0,\theta )= &   -\int \limits _0^\theta R^*(f(s)) ds + 2\sqrt{2} \int \limits _0^\theta \sqrt{R^*_X(f(s))f'_X(s)} ds \nonumber \\  &   + 2\sqrt{2}\int \limits _0^\theta \sqrt{R^*_Y(f(s))f'_Y(s)} ds - f_X(\theta ) - f_Y(\theta ). \end{aligned}$$

Since either *f* is continuous at $\theta$, or $\theta =1$, by Lemma C.1 we have

$$\begin{aligned}f_{n,X}(\theta ) + f_{n,Y}(\theta ) \rightarrow f_X(\theta ) + f_Y(\theta ).\end{aligned}$$

Since *f* is continuous almost everywhere, by Lemma C.1 and the continuity of $R^*$ away from 0 (using the fact that $f_n,f\in G_M^2$), we have

$$\begin{aligned}\int \limits _0^\theta R^*(f_n(s)) ds \rightarrow \int \limits _0^\theta R^*(f(s)) ds.\end{aligned}$$

The result then follows from Lemma C.4 and the symmetry between the *X* and *Y* components.

Proposition 2.10 follows easily from Lemma 7.2.

#### Proof of Proposition 2.10

By Lemma 7.1 we know that $G_{M,T}^2$ is totally bounded, and since $G_M^2\subset G_{M,T}^2$ and is closed, and $(E^2,d)$ is complete, we deduce that $G_M^2$ is compact under *d*.

We now prove the statement about a closed set *F*. For each $n\in \mathbb {N}$, take $f_n\in B_d(F,1/n)\cap G_M^2$ such that

$$\begin{aligned} K(f_n,0,1)\ge \sup _{f\in B_d(F,1/n)\cap G_M^2} K(f,0,1) - 1/n. \end{aligned}$$

Since $G_M$ is compact there exists a subsequence $(f_{n_j})_{j\ge 1}$ such that $d(f_{n_j},f_\infty )\rightarrow 0$ as $j\rightarrow \infty$ for some $f_\infty \in G_M^2$. Since $d(f_{n_j},f_\infty )\rightarrow 0$, and *F* is closed, we also have $f_\infty \in F$. By Lemma 7.2

$$\begin{aligned} \limsup _{j\rightarrow \infty } K(f_{n_j},0,1)\le K(f_\infty ,0,1). \end{aligned}$$

Then by our choice of $f_n$,

$$\begin{aligned} \limsup _{j\rightarrow \infty } \hspace{-1mm} \sup _{f\in B_d(F,1/n_j)\cap G_M^2} \hspace{-2mm} K(f,0,1)&\le \limsup _{j\rightarrow \infty } (K(f_{n_j},0,1) + 1/n_j)\\&\le K(f_\infty ,0,1) \le \sup _{f\in F \cap G_M^2} \hspace{-2mm} K(f,0,1) \end{aligned}$$

which completes the proof of the statement about the closed set *F*. The final part, about a function *f* that is continuous at $\theta$, is almost identical, with $F=\{f\}$ and using $K(\cdot ,0,\theta )$ in place of $K(\cdot ,0,1)$ throughout.
